# A new donor for charge-transfer systems: synthesis and properties of 2,4,7,9-tetramethyl-1,6-dithiapyrene (TMDTP) and structure of (TMDTP)_3_(PF_6_)_2_·2THF and TMDTP–TCNQ†

**DOI:** 10.1039/d1ra00587a

**Published:** 2021-04-20

**Authors:** Jesper Bendix, Klaus Bechgaard, Jørn Bolstad Christensen

**Affiliations:** Department of Chemistry, University of Copenhagen Universitetsparken 5 DK-2100 Copenhagen Denmark; Department of Chemistry, University of Copenhagen Thorvaldsensvej 40 DK-1871 Frederiksberg Denmark jbc@chem.ku.dk

## Abstract

The heterocyclic donor molecule 2,4,7,9-tetramethyl-1,6-dithiapyrene (TMDTP) has been synthesized in five steps. Oxidation of TMDTP is facile (*E*_1_^1/2^ = 0.27 V and *E*_2_^1/2^ = 0.79 V *vs.* SCE). The charge-transfer complex, TMDTP–TCNQ, has been prepared and the salt, (TMDTP)_3_(PF_6_)_2_·2THF, obtained by electrocrystallization. The structure of TMDTP, TMDTP–TCNQ and (TMDTP)_3_(PF_6_)_2_·2THF has been characterized by X-ray crystallography and computationally.

## Abstract 2

The heterocyclic donor molecule 2,4,7,9-tetramethyl-1,6-dithiapyrene (TMDTP) has been synthesized in five steps. The charge-transfer complex, TMDTP–TCNQ, has been prepared and the salt, (TMDTP)_3_(PF6)_2_·2THF, obtained by electrocrystallization.

## Graphical abstract

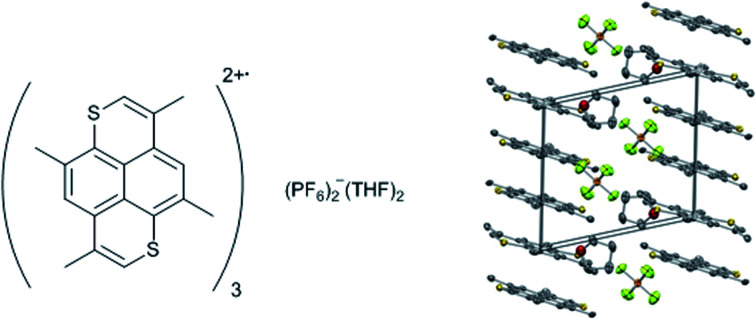

## Introduction

Polycyclic aromatic compounds are interesting in materials science for applications in organic electronics. Some of the earliest known organic semiconductors were radical cation salts of pyrene and perylene^1^ formed by reaction with bromine. The high oxidation potentials of most polycyclic aromatic compounds make them less attractive for applications due to the high reactivity of the corresponding radical cations. However, they can still be highly useful in devices like OLEDs and FETs.^2,3^ Recently, radical cation salts have also gained interest because some of them have a high thermoelectric effect making them highly interesting for small generators of electricity.^4^

**Fig. 1:**
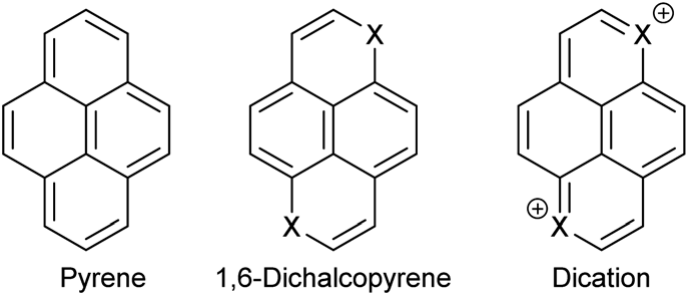
Pyrene (16 π system), a 1,6-dichalcopyrene (18 π system) and the corresponding dichalcopyrene dication (16 π system).

Having large conjugated π-systems in conducting organic materials is advantageous because the transport of charge takes place through the stacked molecules and having a large contact surface should lead to smaller on-site electron to electron repulsion and a higher transfer integral in terms of the Hubbard-model.^5^ Furthermore, the hydrogen atoms on the periphery of a polycyclic aromatic compound can be substituted with groups that can be used to engineer the packing in the solid state and to influence the redox potentials. Substitution of CH-groups in a polycyclic aromatic compound like pyrene with chalcogen atoms lead to systems, where the dications are isoelectronic with pyrene itself (Fig. 1) and the corresponding oxidation potentials are therefore expected to be lower leading to more stable materials.

The effect of substituting CH-groups with chalcogens has been a topic of interest for a long time. The discovery of the first organic metal TTF-TCNQ in 1973 led to an interest in radical cation salts and the discovery of first organic superconductors based on tetramethyltetraselenafulvalene (TMTSF) (TMTSF)_2_PF_6_ (superconducting at 0.9 K and 12 kbar)^6^] and (TMTSF)_2_ClO_4_ (superconducting at 1.3 K and atmospheric pressure).^7^ The corresponding sulphur analogue (tetramethyltetrathiafulvalene) forms a series of isostructural radical cation salts, but none becomes superconducting. In fact, the Bechgaard-salts have a rich phase diagram, where the solid-state properties depend on the anion, temperature, pressure, magnetic and electric fields.^8^ Replacement of sulphur and selenium with the other group 16 elements tellurium and oxygen was a holy grail for many years, and although the first tetratellurofulvalene was synthesized in 1982, it has turned out to be very difficult to obtain any crystalline molecular solids allowing for comparison of properties. Tetraoxafulvalene itself is still unknown and the closest derivatives are the dibenzo- and dinaphtotetraoxafulvalenes,^9^ dibenzotrioxathio-,^10^the unsymmetrical dibenzodithiafulvalene^11^ and dibenzodiselenadioxafulvalene.^12^ A crystalline 1 : 1 charge transfer complex between *trans*-dibenzodithiadioxafulvalene and TCNQ has been reported as being an insulator but no crystal structure was described.^11^

**Scheme 1:**
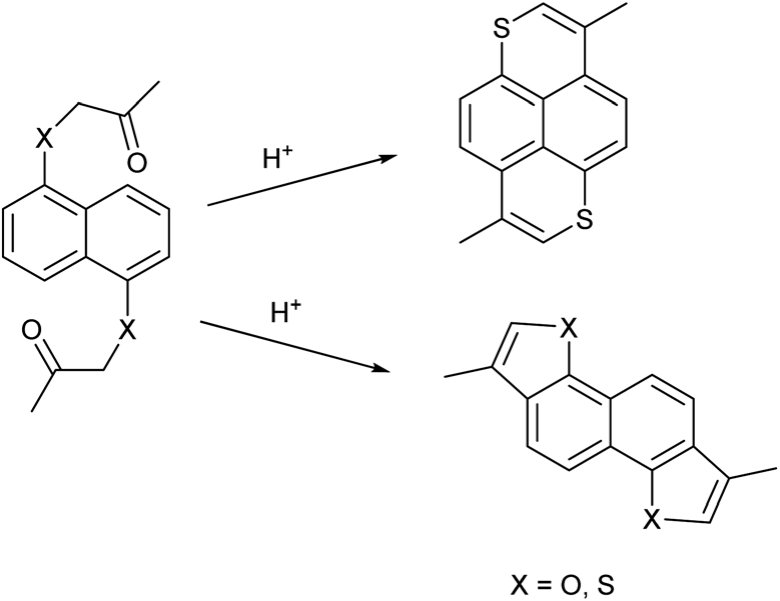
The different regioselectivities in acid catalyzed cyclizations of 1,1′-(naphthalene-1,5-diylbis(oxy))bis(propan-2-one) and 1,1′-(naphthalene-1,5-diylbis(sulfanediyl))bis(propan-2-one).

The groups of Bechgaard^13–15^ and Nakasuji^16–29^ have reported conducting molecular solids based on 1,6-dithiapyrene (1), which was originally synthesized by Tilak and coworkers.^30,31^ This led to the question if it would be possible to use the pyrene π-frame as a test tube for investigating the effect of the heteroatom on the conducting materials properties. Since it is not possible to synthesize 1,6-dioxapyrenes by the same methodology^32^ (Scheme 1), different methodologies have been developed based either on 2,6-dialkyl-1,5-naphthalenediols as starting materials^33,34^ or on using 1,4,5,8-tetrasubstituted naphthalenes as intermediates^35–38^ but both strategies have their limitations.

**Fig. 2:**
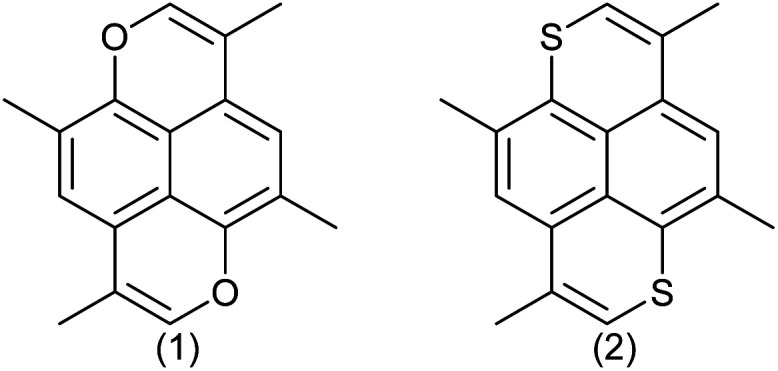
Tetramethyldioxapyrene (TMDOP) (1) and tetramethyldithiapyrene (TMDTP) (2).

So far only a few 1,6-dioxapyrenes have given well-defined charge transfer complexes or radical cations salts, where it has been possible to get a crystal structure. The complex between 2,7-dimethyl-4,9-diethyl-1,6-dioxapyrene and TCNQ is a mixed stack and therefore insulating.^39,40^ Conversely, 2,4,7,9-tetramethyl-1,6-dioxapyrene (TMDOP) was found to form a series of isostructural salts of stoichiometry (TMDOP)_2_X,^33^ where X^−^ is BF_4_^−^, PF_6_^−^ and AsF_6_^−^ with homogeneous donor stacking (Fig. 2).

The crystal structure of these compounds reveal the donor molecules packing in a centrosymmetric arrangement with regular stacking in the *z*-direction, but unfortunately with the TMDOP-molecules rotated in the *x*–*y* plane minimizing the molecular overlap in the stacks^40,41^ and the salts are semiconducting.^42^ The discovery of the (TMDOP)_2_X series led us to synthesize the corresponding sulphur analogue 2,4,7,9-tetramethyl-1,6-dithiapyrene (TMDTP) hoping that it would be possible to obtain an isostructural radical cation salt in order to compare the influence of the heteroatom on the properties. A previous communication^42^ reported metallic conductivity of the salt (TMDTP)_2_AsF_6_ down to 75 K, where charge localization accompanied a metal to insulator transition. The crystals were unfortunately too disordered to obtain a crystal structure.^42^ The present work reports on the synthesis and properties of TMDTP, the structure of a 1 : 1 TCNQ-complex (8) and the new radical cation salt (TMDTP)_2_AsF_6_ (9) as well as a comparison of the physical properties with the corresponding oxygen analogue TMDOP.

## Results and discussion

### Synthesis

**Scheme 2:**
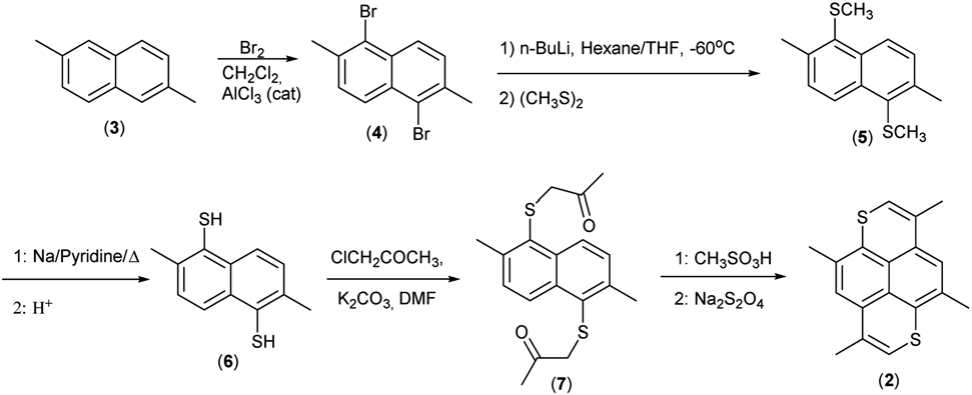
Synthesis of 2.

The synthesis is based on our previous work on 2,6-disubstituted-1,6-dioxapyrenes,^33,34^ where the common intermediates are 2,6-disubstituted-1,5-naphthalenediols. Here the analogous intermediate would be 2,6-dimethyl-1,5-napthalenedithiol (6), which is convenient for synthesis of other derivatives for a more thorough comparison between 1,6-dithia- and 1,6-dioxapyrenes. 3,5,8,10-Tetramethyl-1,6-dithiapyrene (2) was synthesized as shown in Scheme 2 from a mixture of 2,6-dimethylnaphthalene and 2,7-dimethylnaphthalene (easily obtainable by crystallization of commercial dimethylnaphthalene/ethylnaphtalene mixture). Reaction with bromine and a catalytic amount of AlCl_3_ in CH_2_Cl_2_ gave pure 1,5-dibromo-2,6-dimethylnaphthalene (4) crystallizing from the reaction mixture. Transmetallation with *n*-BuLi in THF at −78 °C gave 1,5-dilithio-2,6-dimethylnaphthalene, which was trapped with dimethyldisulfide to give the sulphide (5). Reductive cleavage with sodium in pyridine gave the dithiol (6), which was alkylated with chloropropanone in DMF with K_2_CO_3_ as base. The ketosulfide (7) was cyclized to yield TMDTP (2) with CH_3_SO_3_H in CH_2_Cl_2_.

Crystals of the TCNQ-complex (8) were grown by diffusion in a H-tube using acetonitrile as the solvent. The stoichiometry was determined by elemental analysis and confirmed by X-ray crystallography.

Crystals of the (TMDTP)_3_(PF_6_)_2_·2THF (9) were grown by electrocrystallization of a solution of TMDTP in THF with Bu_4_NPF_6_ as electrolyte.

### Comparison between TMDOP and TMDTP

The oxidation potentials for the two compounds were measured by cyclic voltammetry and are shown in Table 1.

**Table 1:** Electrochemical data^a^

Compound	*E*_1_^1/2^, V	*E*_2_^1/2^, V	Δ*E*, V
TMDOP^33^	0.32	1.00	0.68
TMDTP	0.27	0.79	0.52

a Potentials were measured at a scan rate of 100 mV s^−1^ using Pt *versus* SCE in 0.1 M Bu_4_NPF_6_/CH_2_Cl_2_.

The first oxidation potential of TMDTP is slightly lower than that for TMDOP, but the second oxidation potential is significantly lower for TMDTP than for TMDOP and the difference Δ*E* suggests that the on-site electron repulsion (*U* in the Hubbard model) is lower for TMDP than for TMDOP.

### Structural characterization

**Fig. 3:**
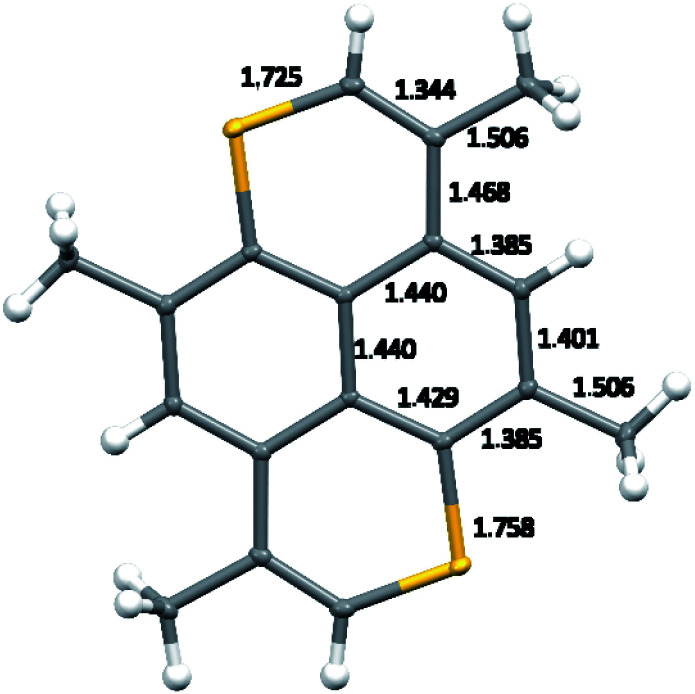
Molecular structure of TMDTP including bond lengths in the asymmetric unit. Thermal ellipsoid probabilities are drawn at 50%.

Crystal structures of TMDTP, TMDTP–TCNQ (8), and (TMDTP)_3_(PF_6_)_2_·2THF (9) were determined by single crystal X-ray diffraction at 100 K. TMDTP crystallizes in the triclinic group *P*1̄ with TMDTP at inversion centers. The normal to the TMDTP planes makes an angle of *ca.* 21.8° with the crystallographic *a*-axis and a TMDTP interplane distance at 3.515 Å, accordingly shorter than the unit cell *a*-axis (3.962 Å). The molecular structure including bond length metrics is shown in Fig. 3.

**Fig. 4:**
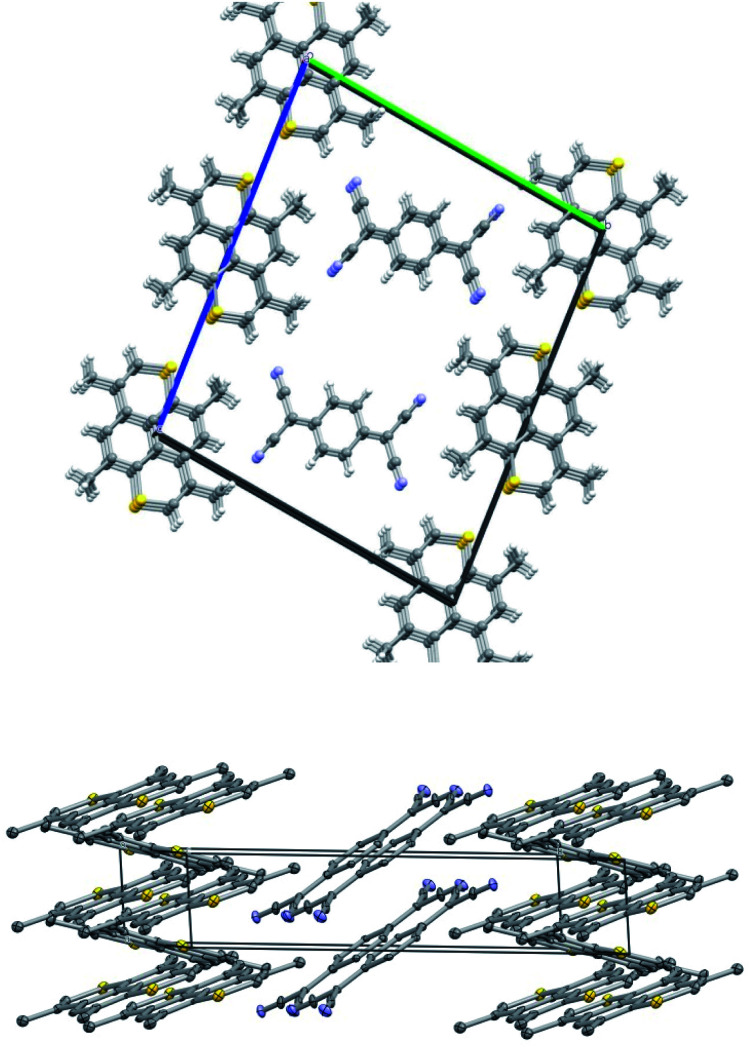
Crystal packing of TMDTP·TCNQ. Top view approx. along *a*-axis. Bottom view approx. along *c*-axis. Thermal ellipsoid probabilities are drawn at 50%. The asymmetric unit comprises half a molecule of TMDTP from each of the two differently inclined stacks and a full TCNQ unit. Hydrogens are omitted for clarity. Color coding: carbon: grey; sulfur: yellow nitrogen: blue.

Crystals of TMDTP–TCNQ (8) also belong to *P*1̄ with TMDTP at inversion centers. In addition TCNQ units are at general positions. The packing is shown in Fig. 4 and features a stack of TCNQ and two different stacks of TMDTP. One stack (a) is close to aligned with the TCNQ stack: ∠TCNQ–TMDTP_a_ = 13.94° whereas the second TMDTP stack (b) is significantly inclined with respect to the TCNQ stack: ∠TCNQ–TMDTP_b_ = 50.92°. The angle between the two TMDTP stacks amounts to 36.98°. The interlayer distances in the three stacks are TCNQ: 3.132 Å; TMDTP_a_: 3.538 Å; TMDTP_b_: 3.531 Å. From the metrics of the TCNQ moiety, an analysis of the extent of charge transfer can be made based on the modeling by Coppens *et.al*.^43^ For TCNQ–TMDTP the distance based model of ref. 45, based on the full dataset yield *q* = −0.69. This is close to the calculated charge transfer, *q* = −0.70(3), based on the same model for monomethylmorpholinium (TCNQ)_2_, but significantly larger the charge transfer for tetramethyltetraselenofulvalene (red form, *q* = −0.27(6)), testifying to the donor capabilities of TMDTP.

**Fig. 5:**
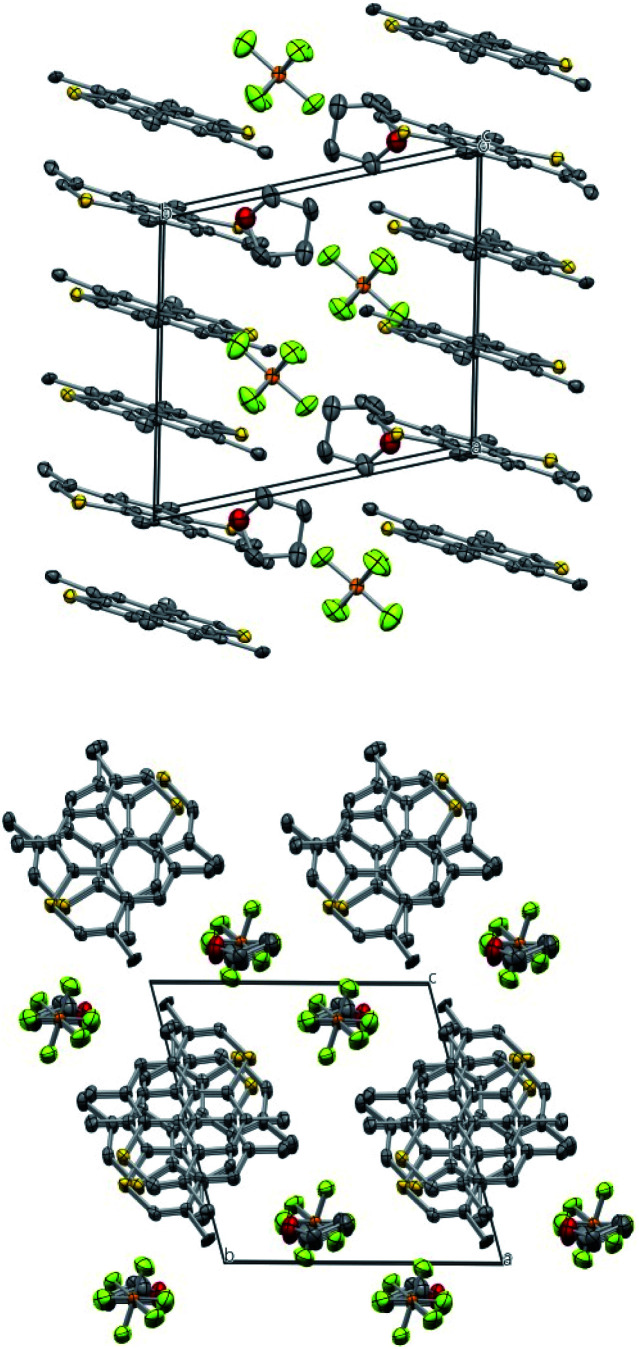
Crystal packing of (TMDTP)_3_(PF_6_)_2_·2THF. Top view approx. along *c*-axis. Bottom view approx. along *a*-axis. Thermal ellipsoid probabilities are drawn at 40%. The asymmetric unit comprises 1.5 molecule of TMDTP, one PF_6_^−^ ion and one THF molecule. Hydrogens are omitted for clarity. Color coding: carbon: grey; phosphorous: orange; oxygen: red; fluorine: lime.

A needle-shaped crystal of TMDTP electrocrystallized with PF_6_^−^ as counter ion in a 3 : 2 stoichiometry was investigated by single crystal X-ray diffraction. The compound crystallizes in space group *P*1̄ with 1/3's of TMDTP cations at inversion centers and all other moieties at general positions. The packing consists of TMDTP cation stacks, with interlayer spacing of 3.608 Å running along the crystallographic *a*-direction (*cf.*Fig. 5 top panel). Separating the TMDTP stacks are columns of alternating THF molecules and PF_6_^−^ anions (Fig. 5 bottom panel). By symmetry the closest spacings between the TMDTP stacks equals the crystallographic *b* = 11.925(4) Å and *c* = 12.194(4) Å dimensions.

Along the TMDTP stacks the molecular orientation alternate between layers, but neighbouring sulfur atoms remain in quite close proximity (3.841 Å/3.912 Å). Both the stoichiometry and the metrics of the TMDTPs indicate that the two moieties with fractional coordinates *a* = 1/3 and *a* = 2/3 are oxidized with average C–S bond lengths 1.710 Å while moieties with *a* = 0 are neutral (average C–S bond length = 1.747 Å). The difference upon oxidation matches quite closely the computed average change of −0.030 Å upon oxidation (*vide infra*).

## Discussion

**Fig. 6:**
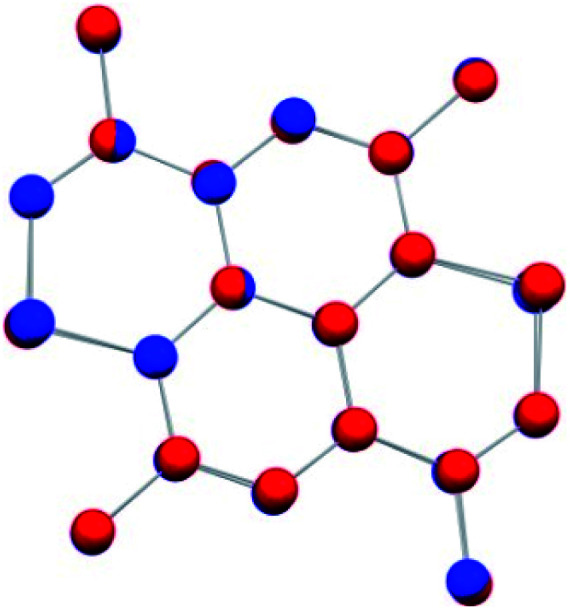
Overlay of TMDTP moieties from TCNQ–TMDTP (blue) and (TMDTP)_3_(PF_6_)_2_·2THF (red). The rms deviation on atom positions is 0.016 Å.

The degree of charge transfer in TCNQ–TMDTP inferred by analysis of the TCNQ geometry (*vide supra*) according to Coppens and coworkers,^43^ is further corroborated by comparison of the structures of the TMDTP entities between the TCNQ–TMDTP and the (TMDTP)_3_(PF_6_)_2_·2THF structures. The stoichiometry of the latter compound together with the model derived charge of *q* = +0.69 for TMDTP in TCNQ–TMDTP suggest very similar average oxidation states of TMDTP in the two structures. Indeed, a structure overlay of TMDTP from both compounds reveal almost perfectly superimposable geometries (*cf.*Fig. 6) with only very minor differences (<0.02 Å) in bond lengths and planarity (<0.14 Å) mainly around the sulfur atoms.

**Fig. 7:**
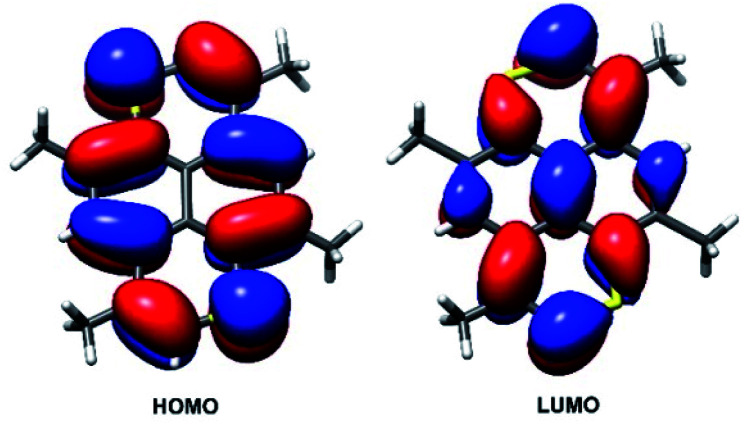
DFT computed frontier orbitals for neutral TMDTP. Restricted computation employing the B3LYP functional and a TZVPP basis on all atoms. Contours are draw at (±0.027).

The changes in geometric and electronic structure associated with the oxidation of TMDTP was evaluated computationally using DFT. Frontier orbitals for the neutral molecule are shown in Fig. 7. As for the parent hydrocarbon,^44^ the computed HOMO is located at the periphery of the molecule with approximately equal amplitude at carbon atoms and sulphur atoms. Conversely, the LUMO extends over also the central carbon atoms in the pyrene framework.

**Fig. 8:**
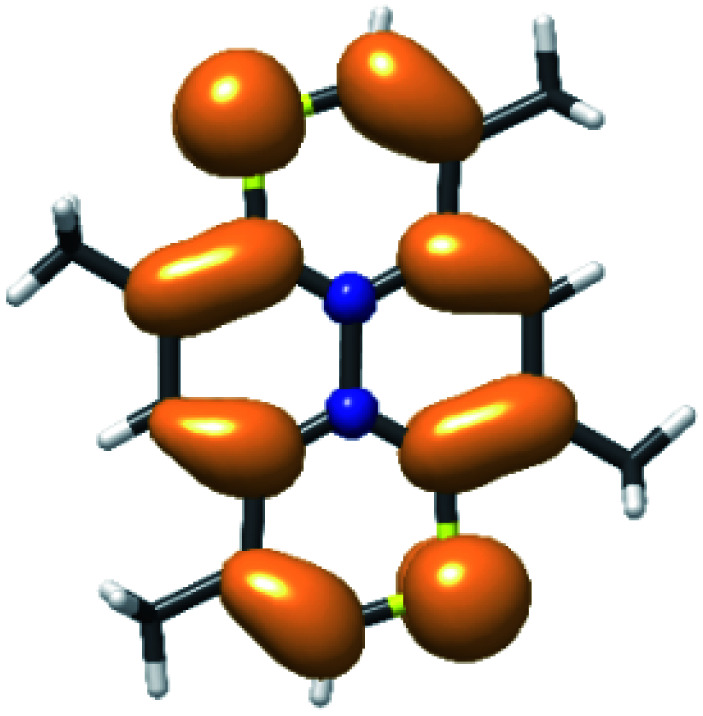
Spin density distribution from unrestricted DFT (ochre = majority; blue = minority). Contours are draw at (±0.001).

Upon oxidation the resulting SOMO resembles the HOMO of the neutral molecule as witnessed by the spin density distribution (Fig. 8).

**Fig. 9:**
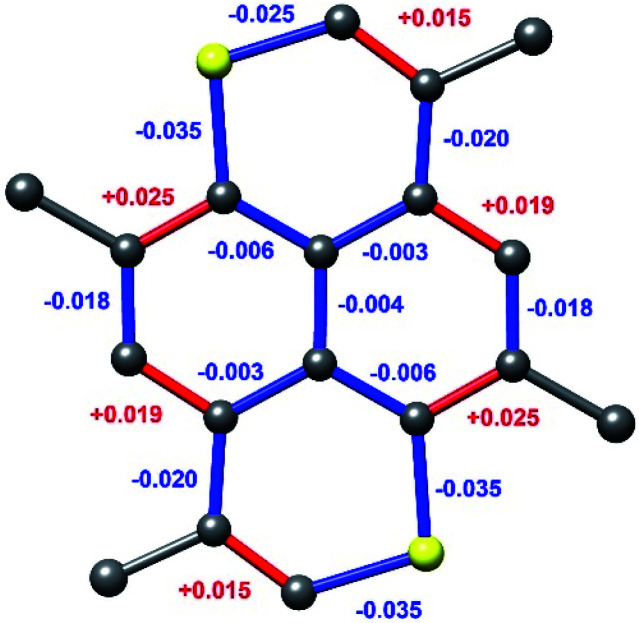
Changes in bond lengths between geometry optimized structures of TMDTP/TMDTP^+^. Differences are changes upon oxidation.

Geometry optimized bond length differences of the TMDTP and its cation are depicted in Fig. 9. The computed changes in geometry reflect the peripheral and very delocalized nature of the MO involved in the redox process. Accordingly, the charge distribution is also very even with the highest computed positive atomic charges being as low as +0.14 (on hydrogens) and only +0.11 on the sulphur atoms, which harbours the highest spin density (+0.18).

## Experimental

### TMDTP (2)

1,1′-((2,6-Dimethylnaphthalene-1,5-diyl)bis(sulfanediyl))-bis(propan-2-one) (7) (1.4 g; 4.2 mmol) was dissolved in CH_2_Cl_2_ (10 mL). After addition of CH_3_SO_3_H (4 mL), the reaction mixture was stirred overnight and poured into a cold solution of NaS_2_O_4_ (10 g; 57 mmol) in 2 M NaOH (100 mL). The crude product was extracted with CH_2_Cl_2_ (500 mL), dried over Na_2_SO_4_ and filtered. The solvent was removed and the product purified by column chromatography on silica gel 60 (0.040–0.063 mm) with toluene as eluent. Yield: 0.28 g (19%) after crystallization from toluene. Mp 206–208 °C

^1^H-NMR (500 MHz, CS_2_, CDCl_3_-lock tube): *δ*: 6.51 (s, 2H); 5.85 (s, 2H); 2.05 (s, 6H); 1.99 (s, 6H).

^13^C-NMR (125 MHz, CS_2_, CDCl_3_-lock tube): *δ*: 132.05; 129.10; 128.99; 128.66; 127.33; 122.76; 117.64; 22.64; 20.25.

EI-MS: *m*/*z*: 296.07 (100.0%), 297.07 (19.5%), 298.07 (9.0%), 298.08 (1.8%), 297.07 (1.6%)

Elemental analysis: calcd for C_18_H_16_S_2_: C, 72.93%; H, 5.44%; S, 21.63%.

Found: C, 73.14%; H, 5.50%; S: 21.66%.

Cyclic voltammetry (0.1 M Bu_4_N^+^PF_6_^−^ in CH_2_Cl_2_, rate: 100 mV s^−1^, Pt *vs.* SCE): *E*_1_^1/2^: 0.27 V; *E*_2_^1/2^: 0.79 V.

### 2,6-/2,7-Dimethylnapthalene mixture (3)

Dimethylnapthalene, mixture of isomers and ethylnaphthalenes (2.5 L) was mixed with EtOH (2.5 L) and cooled to −20 °C for 24 h. The crystalline material was isolated by filtration and air dried to give 476 g of crude material, which was recrystallized from EtOH (3.5 L), cooling to −20 °C, filtered and air dried to give 235 g of 2,6-/2,7-dimethylnaphthalene eutectic mixture. The mixture consists of 58% 2,6-dimethylnaphthalene and 42% 2,7-dimethylnaphthalene as determined by ^1^H-NMR (see ESI†).

### 2,6-Dimethyl-1,5-dibromonapthalene (4)

Bromine (6.6 mL; 20.5 g; 0.26 mol) dissolved in CH_2_Cl_2_ (50 mL) was added over 25 minutes to a stirred solution of (3) (10.0 g; 64 mmol) in CH_2_Cl_2_ (50 mL) containing a catalytic amount of AlCl_3_. Stirring overnight followed by removal of the solvent *in vacuo*. The residue was dissolved in ligroin (bp 100–140 °C, 100 mL), treated with activated carbon and cooled to +5 °C overnight. Yield: 6.7 g (57% based on 2,6-dimethylnaphhalene). Mp 151–153 °C (151–153 °C (ref. 45)).

^1^H-NMR (500 MHz, CDCl_3_): *δ* 8.09 (d, *J* = 5 Hz, 2H); 7.31 (d, *J* = 5 Hz, 2H); 2.52 (s, 6H).

^13^C-NMR (126 MHz, CDCl_3_): *δ* 135.88; 131.94; 129.78; 126.35; 123.95; 24.01.

GC-MS: 314 (M^+^).

### 1,5-Dimethylmercapto-2,6-dimethylnaphthalene (5)

1,5-Dibromo-2,6-dimethylnaphthalene (4) (5.1 g; 16 mmol) was dissolved in dry THF (80 mL) and cooled to −60 °C. A solution of *n*-BuLi in hexane (2.5 M; 13 mL; 32.5 mmol) was added over 10 minutes with stirring. After further 20 min at −60 °C, dimethyldisulfide (3.5 mL; 39 mmol) was added dropwise (exothermic reaction). The cooling bath was removed and when the reaction mixture reached room temperature, it was poured into water (200 mL), extracted with ether, dried over MgSO_4_, filtered and concentrated *in vacuo*. The residual oil was crystallized from absolute ethanol to give pure 5. Yield: 2.5 g (62%). Mp 101–103 °C

^1^H-NMR (500 MHz, CDCl_3_) *δ*: 8.56 (d, *J* = 10 Hz, 2H); 7.40 (d, *J* = 10 Hz, 2H); 2.69 (s, 6H); 2.21 (s, 6H).

^13^C-NMR (126 MHz, CDCl_3_) *δ*: 140.25; 134.48; 132.43; 129.55; 127.02; 21.82; 19.21.

GC-MS: 248.2 (M^+^).

Elemental analysis:

Calcd for C_14_H_16_S_2_: C, 67.69%; H, 6.49%.

Found: C, 66.30%; H, 6.31%.

### 2,6-Dimethylnaphthalene-1,5-dithiol (6)

1,5-Dimethylmercapto-2,6-dimethylnaphthalene (5) (5.0 g; 20 mmol) was dissolved in pyridine (50 mL) under a N_2_-atmosphere. Sodium cut in small pieces (1.9 g; 83 mmol) was added with stirring. The reaction mixture was heated to +100 °C overnight. After cooling to room temperature, the residues of sodium were carefully destroyed with abs. EtOH and the reaction mixture was poured into 2 M H_2_SO_4_ (500 mL). The product was extracted with diethyl ether, dried over Na_2_SO_4_, filtered, concentrated *in vacuo* followed by crystallization from toluene (cooling to −20 °C) to give 1.85 g (42%). Pale yellow material. Mp: 136–138 °C

Elemental analysis:

Calcd for C_12_H_12_S_2_: C, 65.41; H, 5.49; S, 29.10.

Found: C, 65.70; H, 5.47; S, 28.65.

^1^H-NMR (500 MHz, CDCl_3_): *δ*: 8.015 (d, *J* = 5 Hz, 2H); 7.31 (d, *J* = 5 Hz, 2H); 3.32 (s, 2H); 2.51 (s, 6H).

^13^C-NMR (126 MHz, CDCl_3_): *δ*: 134.42; 132.03; 129.20; 127.26; 123.08; 22.42.

ESI-MS: 220 (M^+^).

### 1,1′-((2,6-Dimethylnaphthalene-1,5-diyl)bis(sulfanediyl))bis(propan-2-one) (7)

2,6-Dimethylnaphthalene-1,5-dithiol (6) (1.1 g; 5 mmol) was dissolved in a stirred, degassed mixture of K_2_CO_3_ (2 g; 15 mmol) and DMF (20 mL). 1-Chloro-2-propanone (1.5 mL; 1.7 g; 18 mmol) was added. The reaction mixture was stirred for 2 hours at room temperature, poured into water (200 mL) and extracted with diethyl ether. Drying over Na_2_SO_4_, filtration and removal of the solvent followed by column chromatography on Silicagel 60 (0.040–0.063 mm) with ethyl acetate/heptane (1 : 1). After crystallization from ethyl acetate, pale yellow crystals 2.0 g (66%). Mp 139–141 °C.

^1^H-NMR (500 MHz, CDCl_3_) *δ*: 8.50 (d, *J* = 10 Hz, 2H); 7.40 (d, *J* = 10 Hz, 2H); 3.43 (s, 4H); 2.67 (s, 6H); 2.09 (s, 6H).

^13^C-NMR (126 MHz, CDCl_3_) *δ*: 202.90; 141.47; 134.55; 129.88; 129.36; 127.39; 45.93; 28.74; 22.07.

EI-MS: *m*/*z*: 332.09 (100.0%), 333.09 (19.5%), 334.09 (9.0%), 334.10 (1.8%), 335.09 (1.8%), 333.09 (1.6%)

Elemental analysis: calcd For C_18_H_20_O_2_S_2_: C, 65.03; H, 6.06. Found: C, 65.05; H, 6.20.

### TMDTP–TCNQ (8)

Was grown by diffusion in a H-cell from saturated solutions of TMDTP (2) and TCNQ.

### (TMDTP)_3_(PF_6_)_2_·2THF (9)

A solution of TMDTP (50 mg; 0.17 mmol) in dry THF (50 mL) containing Bu_4_NPF_6_ (0.5 g; 1,3 mmol) was electrocrystallized at room temperature with Pt-electrodes at a constant current of 2 μA for 7 days.

### Crystallography

#### The X-ray crystallographic studies

The X-ray crystallographic studies were carried out on single crystals, which were coated with mineral oil, mounted on kapton loops, and transferred to the nitrogen cold stream of the diffractometer. The single-crystal X-ray diffraction studies were performed at 100(2) K on a Bruker D8 VENTURE diffractometer equipped with a Mo Kα high-brilliance IμS radiation source (*λ* = 0.71073 Å), a multilayer X-ray mirror and a PHOTON 100 CMOS detector, and an Oxford Cryosystems low temperature device. The instrument was controlled with the APEX3 software package using SAINT.^46^ Final cell constants were obtained from least squares fits of several thousand strong reflections. Intensity data were corrected for absorption using intensities of redundant reflections with the program SADABS.^47^ The structures were solved in Olex2 using SHELXT and refined using SHELXL.^48^ The crystals of (TMDTP)_3_(PF_6_)_2_·2THF were all poorly diffracting and the reported resolution of 0.95 Å was the best achievable. Crystallographic details are listed in Table 2. CCDC entries 2051961, 2051922, and 2051918 contain the crystallographic data reported herein.†

**Table 2:** Crystallographic data

Compound	TMTDP	TMDTP·TCNQ	(TMDTP)_3_(PF_6_)_2_·2THF
CCDC entry	2051961	2051922	2051918
Empirical formula	C_18_H_16_S_2_	C_18_H_16_S·C_12_H_4_N_4_	(C_18_H_16_S_2_)_3_·(PF_6_)_2_·(C_4_H_8_O)_2_
Formula weight	296.42	500.62	1323.43
*T*/K	100(2)	100(2)	100(2)
Crystal system	Triclinic	Triclinic	Triclinic
Space group	*P*1̄	*P*1̄	*P*1̄
*a*/Å	3.9618(4)	3.7098(3)	10.825(4)
*b*/Å	9.2110(8)	17.1976(18)	11.925(4)
*c*/Å	9.8478(8)	20.181(2)	12.194(4)
*α*/°	105.537(3)	82.379(4)	71.857(10)
*β*/°	90.439(3)	89.939(3)	77.095(10)
*γ*/°	96.653(3)	86.046(4)	72.995(10)
*V*/Å^3^	343.62(5)	1273.1(2)	1415.2(9)
*Z*	1	2	1
*ρ*_calc_/g cm^−3^	1.432	1.306	1.553
*μ*/mm^−1^	0.373	0.24	0.385
2*θ* range/°	4.3–50.5	4.8–56.4	4.7–43.7
Reflections collected	14 877	69 129	13 127
Independent reflections	2736 [*R*_int_ = 0.050]	5812 [*R*_int_ = 0.071]	3278 [*R*_int_ = 0.084]
Data/restraints/parameters	2736/0/93	5812/0/330	3278/138/385
Goodness-of-fit on *F*^2^	1.078	1.130	1.209
Final *R* indexes [*I* ≥ 2*σ*(*I*)]	*R*_1_ = 0.033, w*R*_2_ = 0.0838	*R*_1_ = 0.096, w*R*_2_ = 0.245	*R*_1_ = 0.139, w*R*_2_ = 0.382
Largest diff. Peak/hole/e Å^−3^	0.55/–0.31	1.34/−0.58	1.03/−0.66

### Computations

DFT calculations were carried out with the ORCA 4.2.1 program Tight convergence criteria were chosen for all calculations (Keywords TightSCF and TightOpt). The B3LYP functional was employed in conjunction with the def2-TZVPP basis set for both geometry optimizations and single point calculations. Orbital and spin density visualizations were done using USCF Chimera ver. 1.13.1.^49^

## Conclusions

A synthesis of 2,4,7,9-tetramethyl-1,6-dithiapyrene (TMDTP) has been developed in order to compare the properties with the corresponding 2,4,7,9-tetramethyl-1,6-dioxapyrene (TMDOP), which is known to form a series of isostructural radical cations salts of the type (TMDOP)_2_X. Replacement of oxygen with sulphur leads to a more promiscuous donor molecule, which forms radical cation salts including crystal solvent molecules that tend to be disordered. The new salt (TMDTP)_3_(PF_6_)_2_·2THF and a 1 : 1 TCNQ-complex were prepared and their structures determined. Structural and computational characterization suggests a closely similar average oxidation state of ∼+2/3 for the TMDTP moieties. The TCNQ-complex consists of segregated stacks in contrast to the known TCNQ-complexes with 1,6-dioxapyrenes. The electrochemical data on TMDTP compared to TMDOP suggests that substitution of two CH-groups in pyrene with sulphur instead of oxygen leads to a better donor-molecule and could lead to new interesting materials.

## Conflicts of interest

There are no conflicts to declare.

## Supplementary Material

RA-011-D1RA00587A-s001

RA-011-D1RA00587A-s002
