# Supplementary material for: A new donor for charge-transfer systems: synthesis and properties of 2,4,7,9-tetramethyl-1,6-dithiapyrene (TMDTP) and structure of (TMDTP)3(PF6)2·2THF and TMDTP–TCNQ
Source: RSC Adv. 2021 Apr 20;11(24):14607–14. doi: 10.1039/d1ra00587a (PMC8697854; doi:10.1039/d1ra00587a)
Supplement: RA-011-D1RA00587A-s001 [file RA-011-D1RA00587A-s001.pdf]

Electronic Supplementary Material (ESI) for RSC Advances.
This journal is © The Royal Society of Chemistry 2021

Supplementary material for Bendix, Bechgaard and Christensen: Synthesis and Properties of 2,4,7,9-Tetramethyl-1,6-dithiapyrene (TMDTP) and Structure of $(TMDTP)_3(PF_6)_2$·2THF and TMDTP-TCNQ.

Table of content:

S1-S18: Determination of composition of the mixture of 2,6- and 2,7-dimethylnaphthalene.

S19-S24: NMR of compound **4**.

S25-S29: NMR of compound **5**.

S30-S35: NMR of compound **6**.

S36-S41: NMR of compound **7**.

S42-S47: NMR of compound **3**.

Full ^{1}H-NMR spectrum of 2,6-Dimethylnapthtthalene in $CDCl_3$

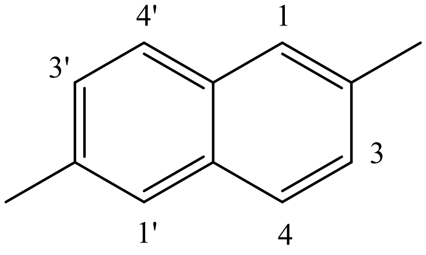

2,6-DMN

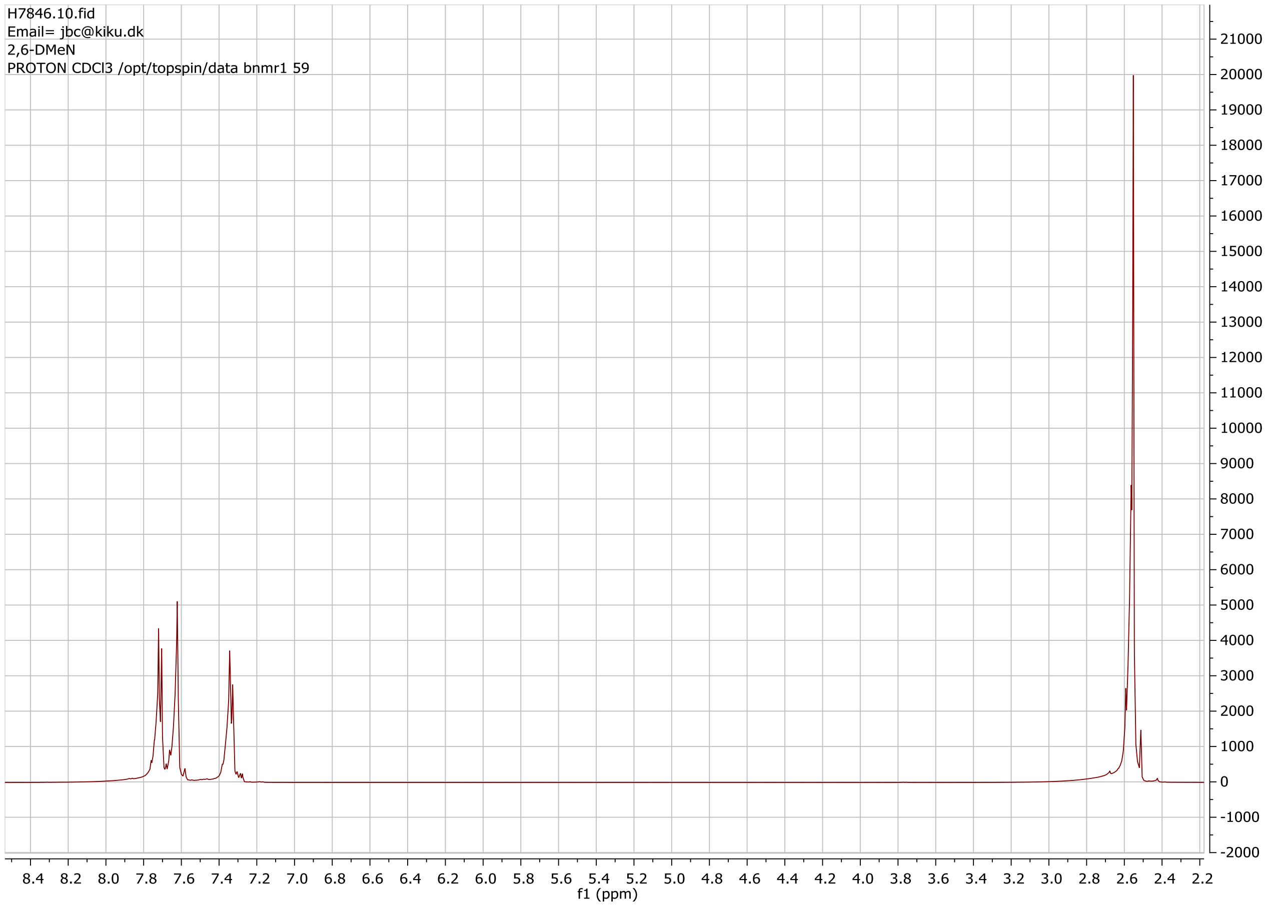

Full ^{1}H-NMR spectrum of 2,7-Dimethylnapththalene (2,7-DMN) in $CDCl_3$

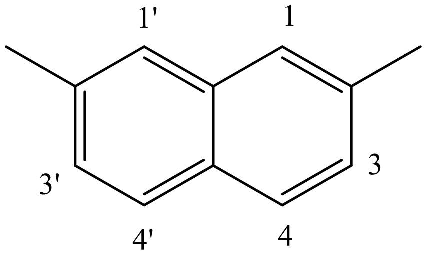

2,7-DMN

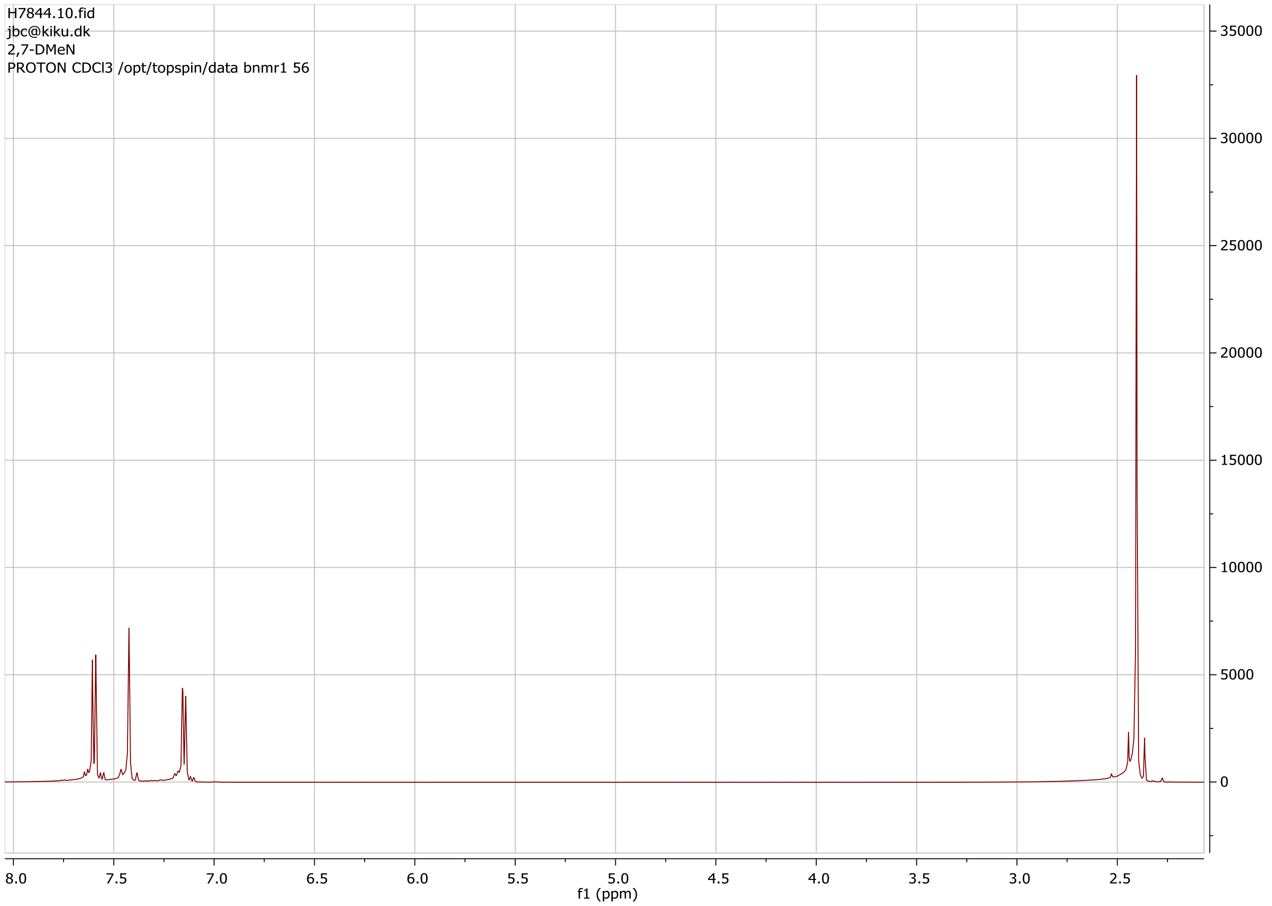
H7844.10.fid
jbc@kiku.dk
2,7-DMeN
PROTON CDCl3 /opt/topspin/data bnmr1 56
35000
30000
25000
20000
15000
10000
5000
0
8.0
7.5
7.0
6.5
6.0
5.5
5.0
4.5
4.0
3.5
3.0
2.5
f1 (ppm)

2,6-DMN and 2,7-DMN interacts in $CDCl_3$ as seen from the ^{1}H-NMR spectra:

Mixture of 2,6- and 2,7-DMN

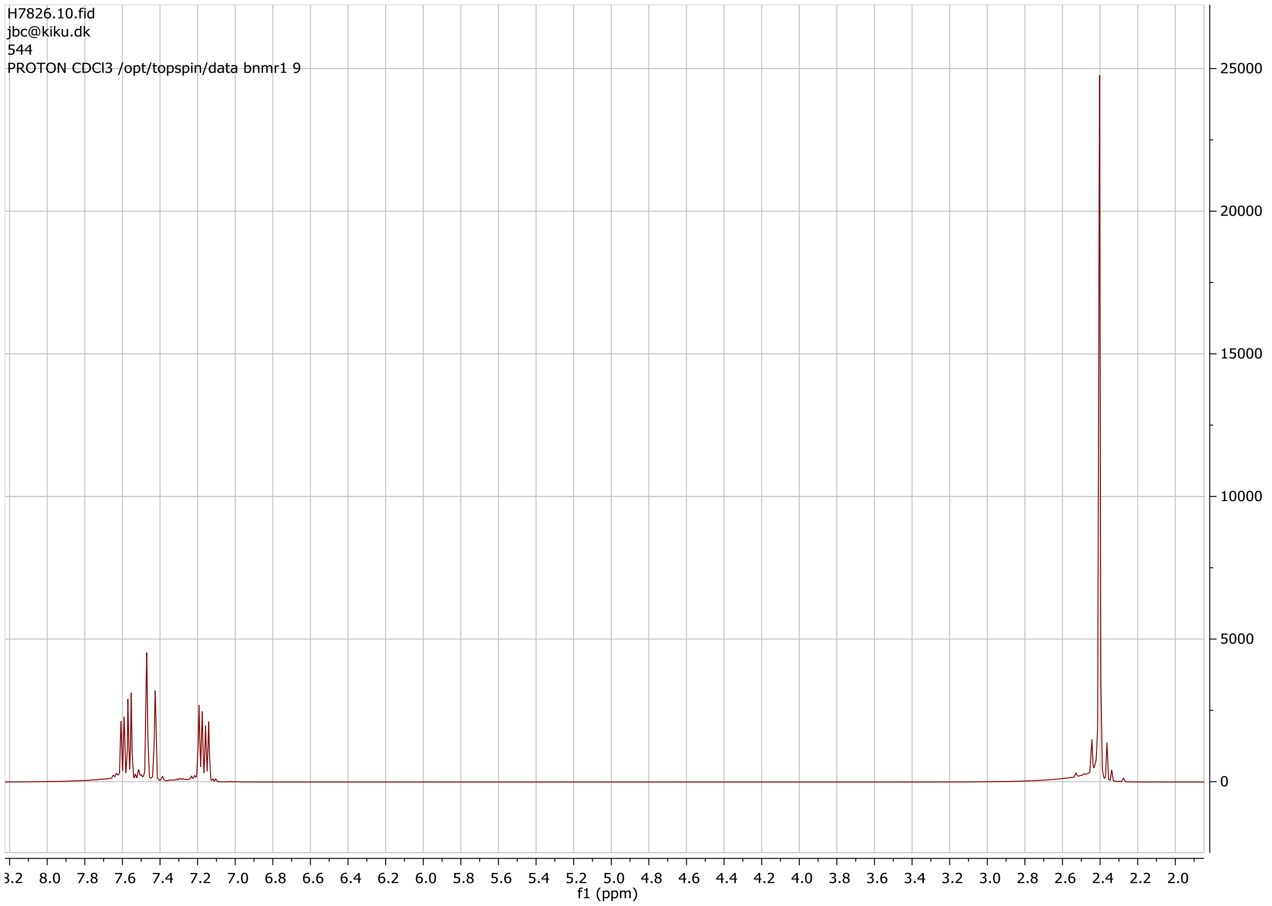

All 3 ^{1}H-spectra expanded in the aromatic area and stacked:

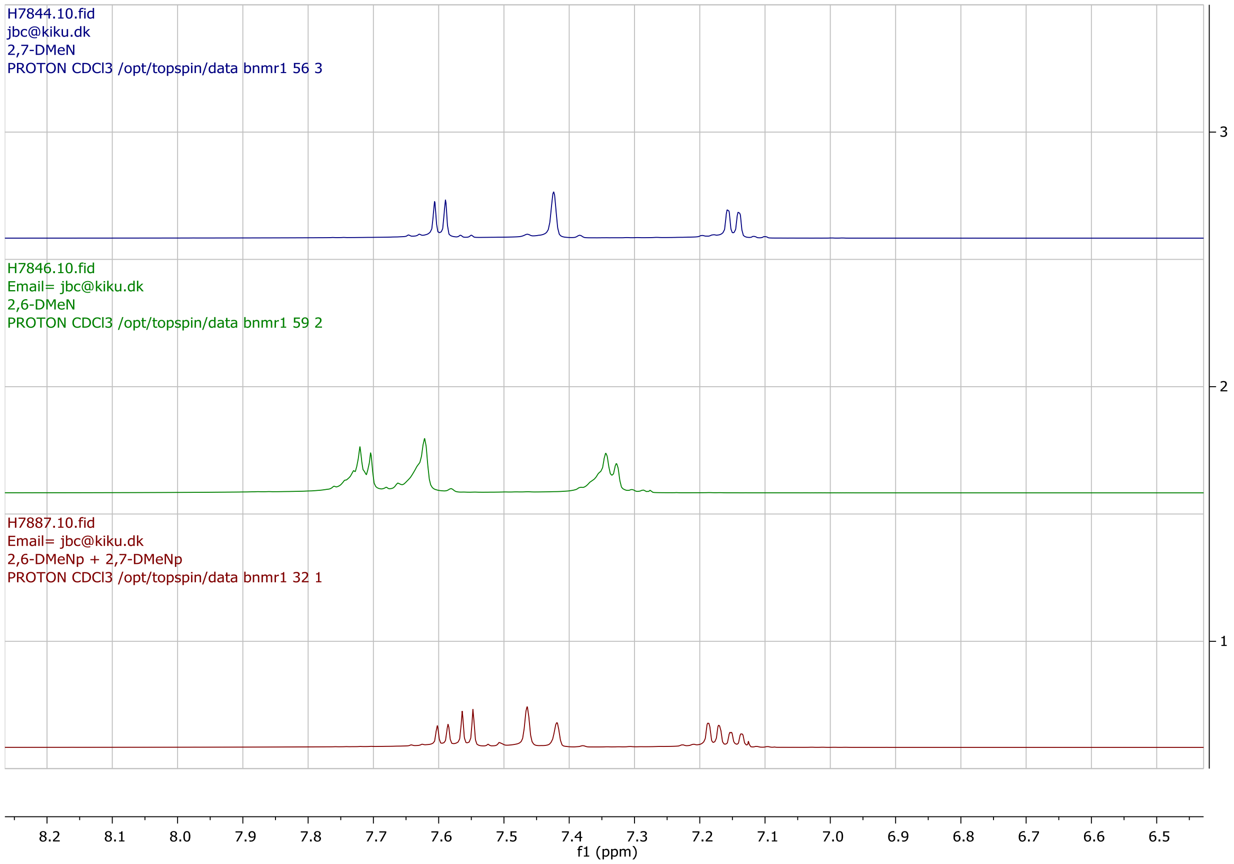

Legend: 2,7-DMN is blue, 2,6-DMN is green and the mixture of 2,6- and 2,7-DMN is red (formed by mixing the solutions of 2,6- and 2,7-DMN).

There is some interaction taking place between the 2,6- and the 2,7-DMN. The carbon-spectra are however unchanged:

All 3 ^{13}C-spectra expanded in the aromatic region and stacked:

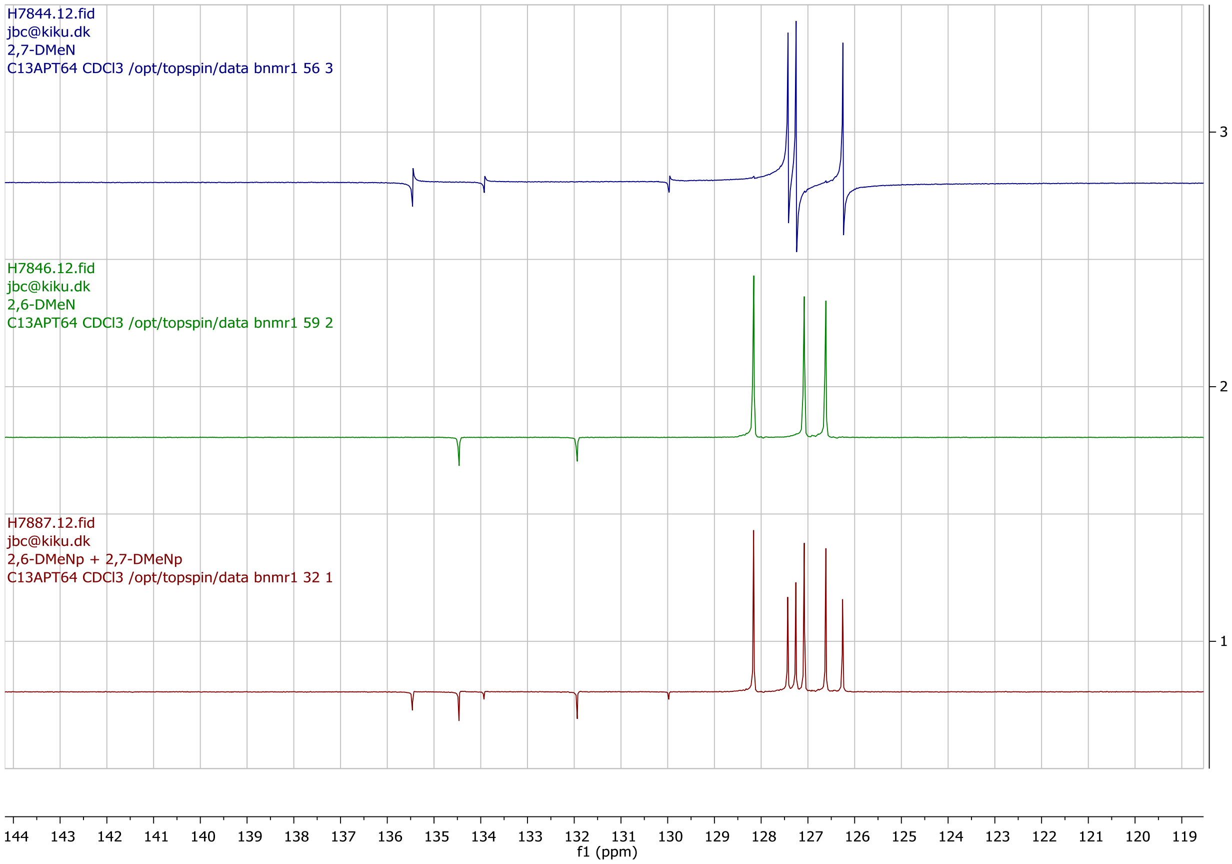

Legend: 2,7-DMN is blue, 2,6-DMN is green and the mixture of 2,6- and 2,7-DMN is red. None of the carbon-signals are shiftet upon mixing the two isomers.

Assigning the protons in 2,6-DMN by 1H-COSY in $CDCl_3$:

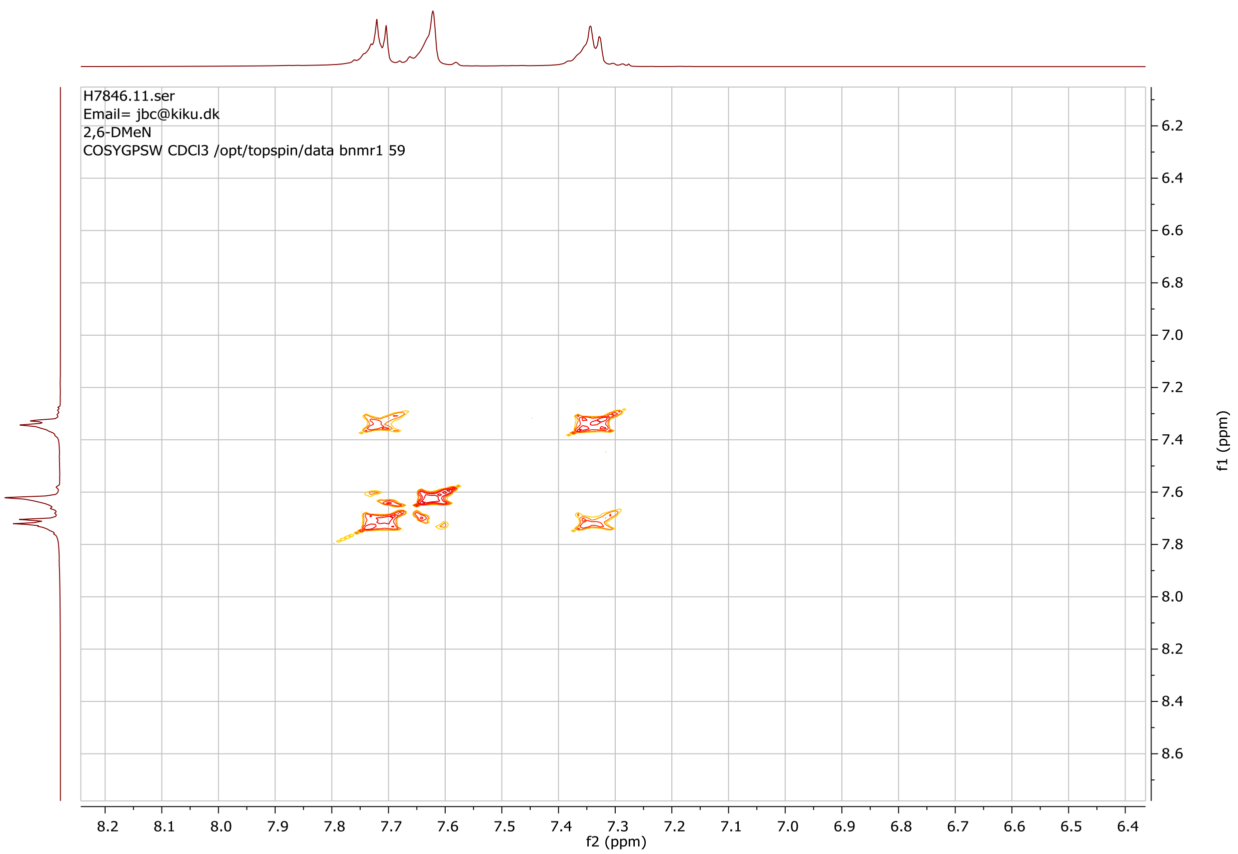

H 1 & H1’: 7.33 ppm (s), but small meta-coupling to H3, H3’

H4, H4’: 7,71 ppm (d, J = 10 Hz)

H3, H3’: 7,34 ppm (d, J = 10 Hz)

HSQC-spectrum of 2,6-DMN in $CDCl_3$ was used to find assign C1/C1’ to H1/H1’:

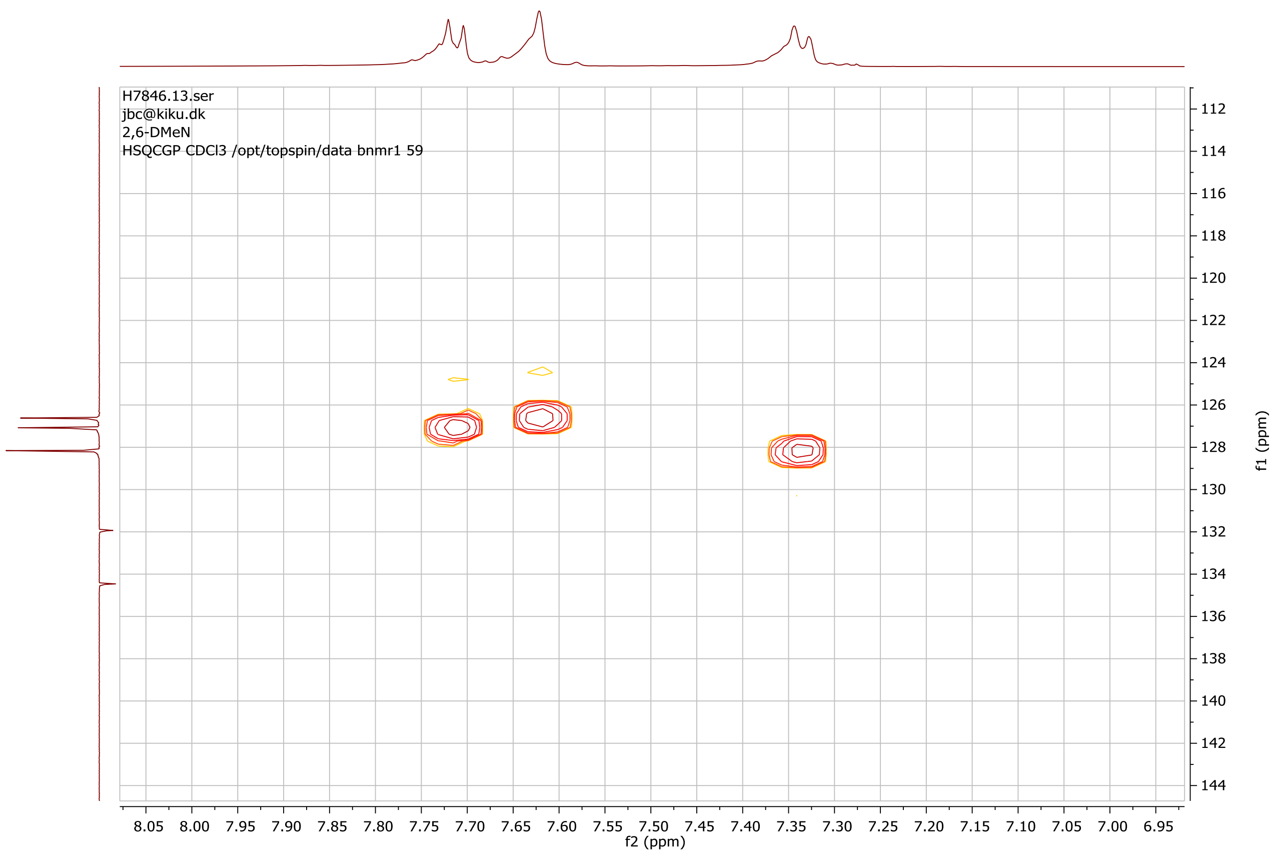

Shows that the carbon at 126.65 ppm is bound to proton 1 and 1’.

This allows identification of H1/H1’ in the mixture of 2,6- and 2,7-DMN:

Assigning the protons in 2,7-DMN by 1H-COSY in $CDCl_3$:

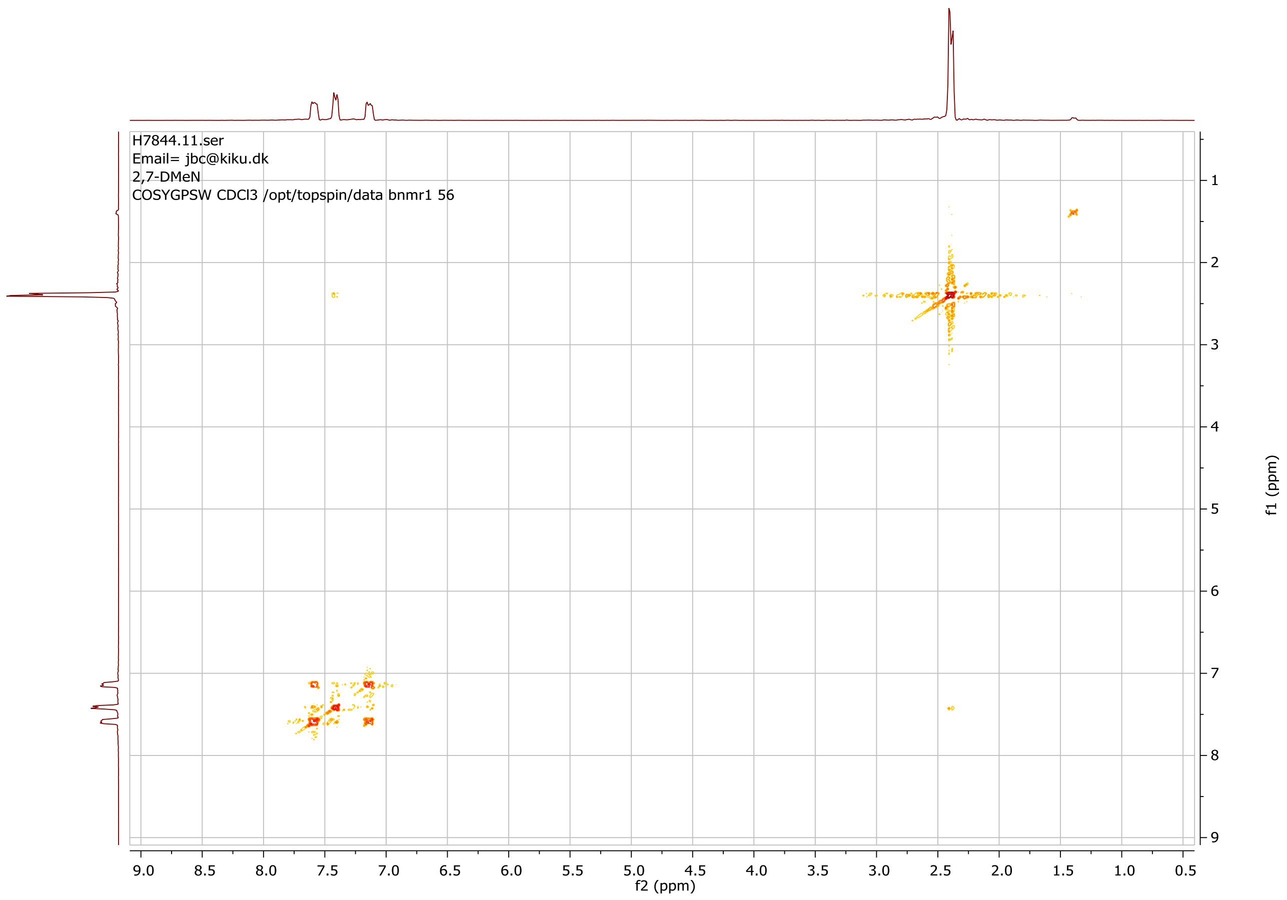

H 1 & H1’: 7.42 ppm (s)

H4, H4’: 7,60 ppm (d, J = 10 Hz)

H3, H3’: 7,34 ppm (d, J = 10 Hz)

HSQC-spectrum of 2,7-DMN in $CDCl_3$ was used to find assign C1/C1' to H1/H1':

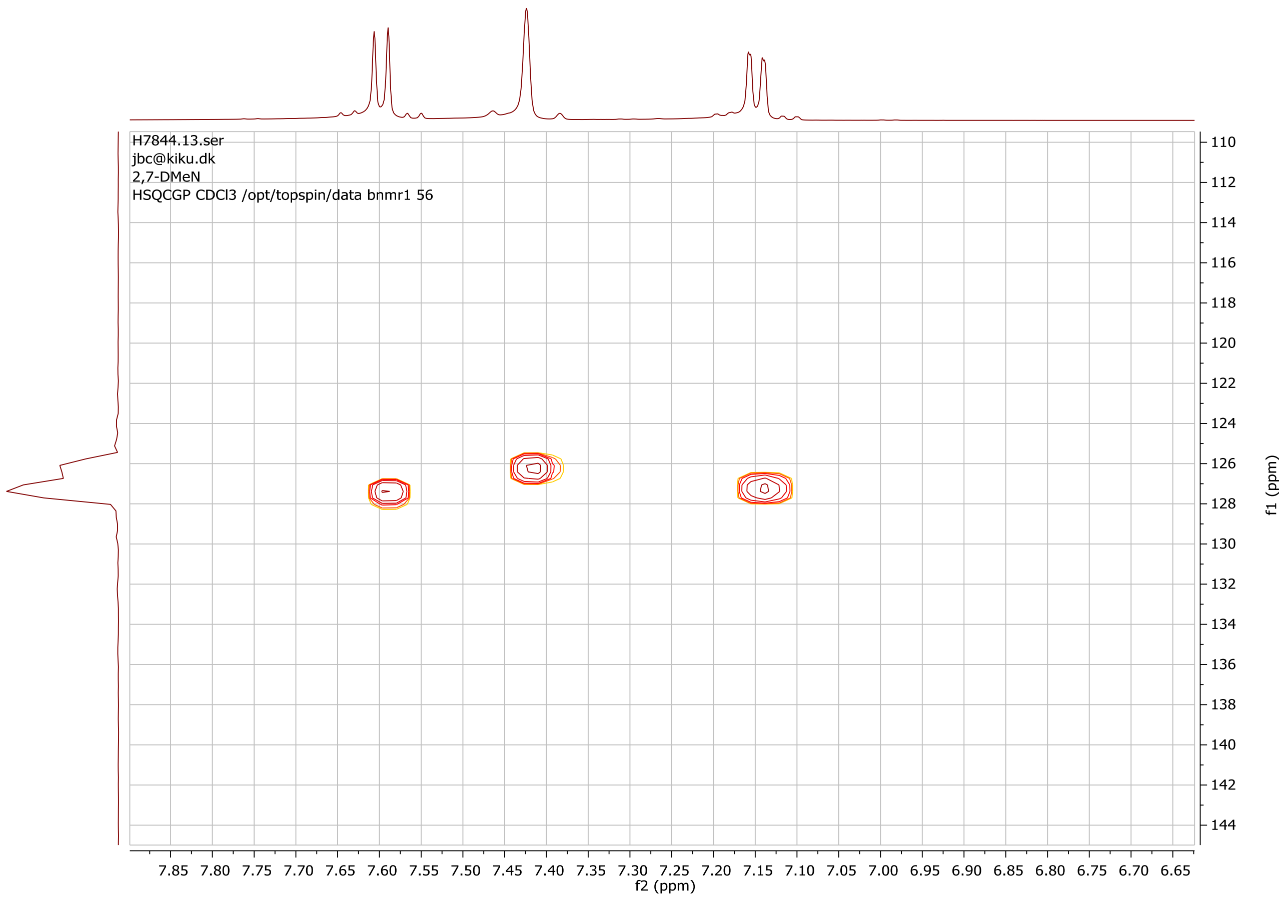

The NMR-spectra in $CDCl_3$ of the eutectic mixture isolated by crystallization looks:

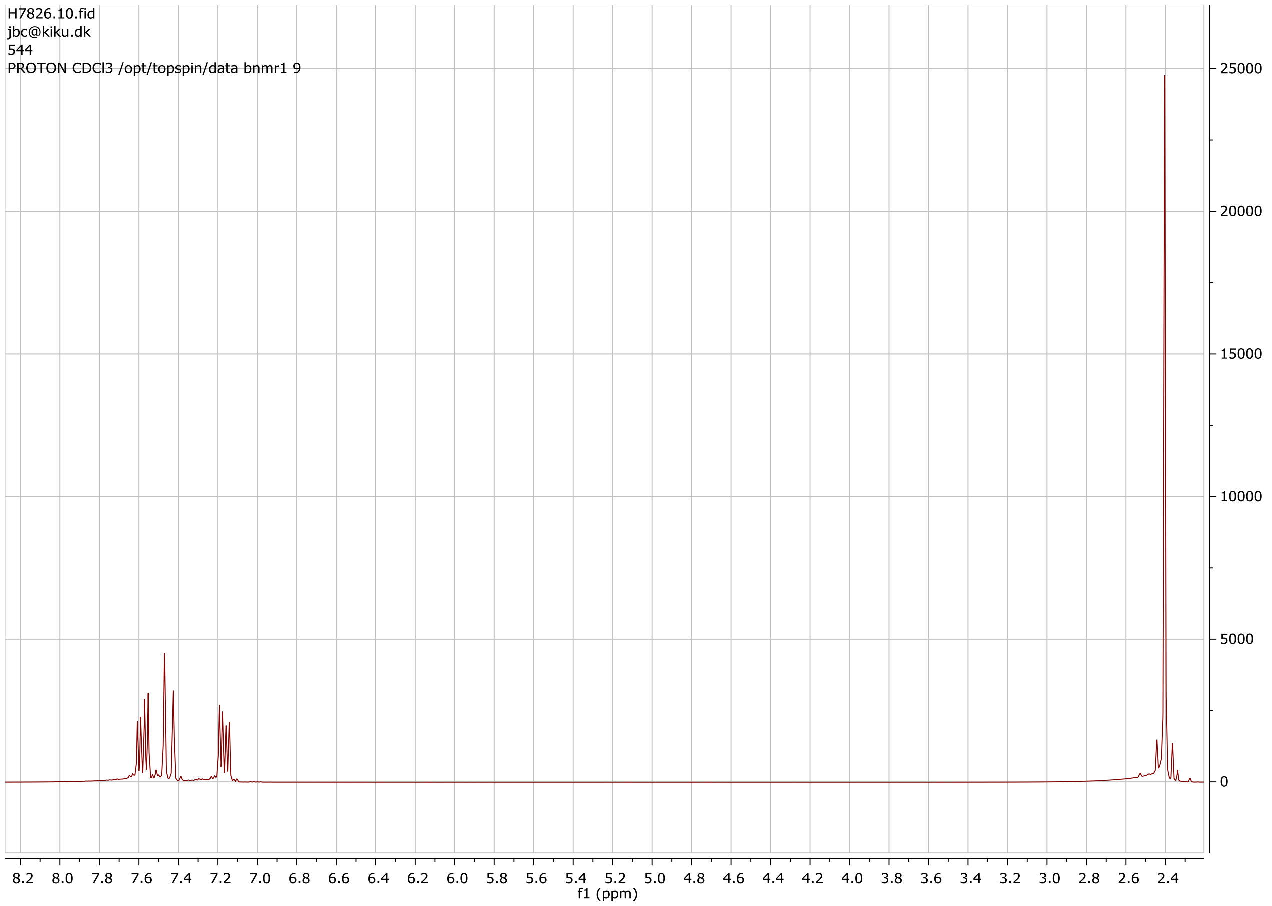

Expanded:

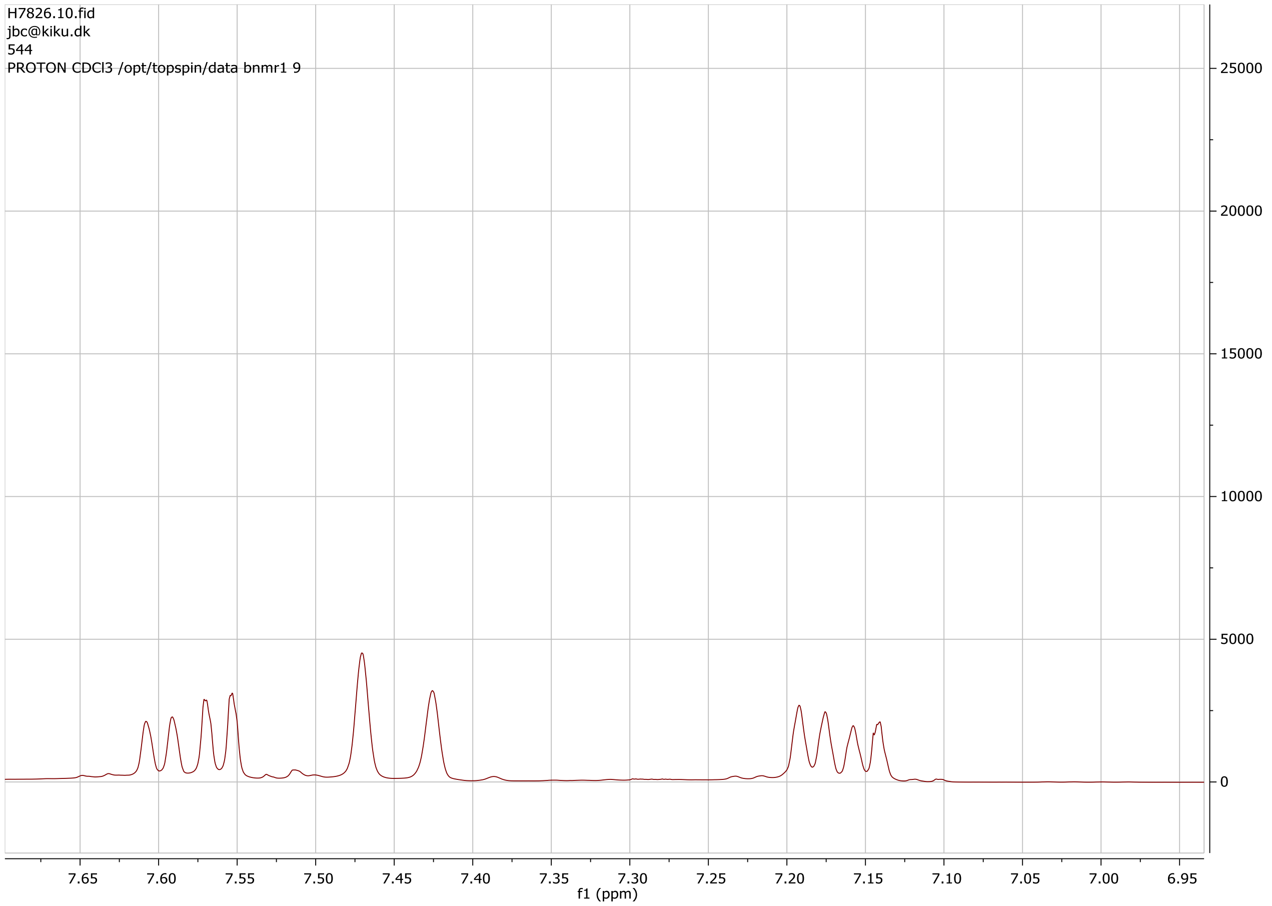

H7826.10.fid
jbc@kiku.dk
544
PROTON CDCl3 /opt/topspin/data bnmr1 9
25000
20000
15000
10000
5000
0
7.65 7.60 7.55 7.50 7.45 7.40 7.35 7.30 7.25 7.20 7.15 7.10 7.05 7.00 6.95
f1 (ppm)

^{1}H-COSY:

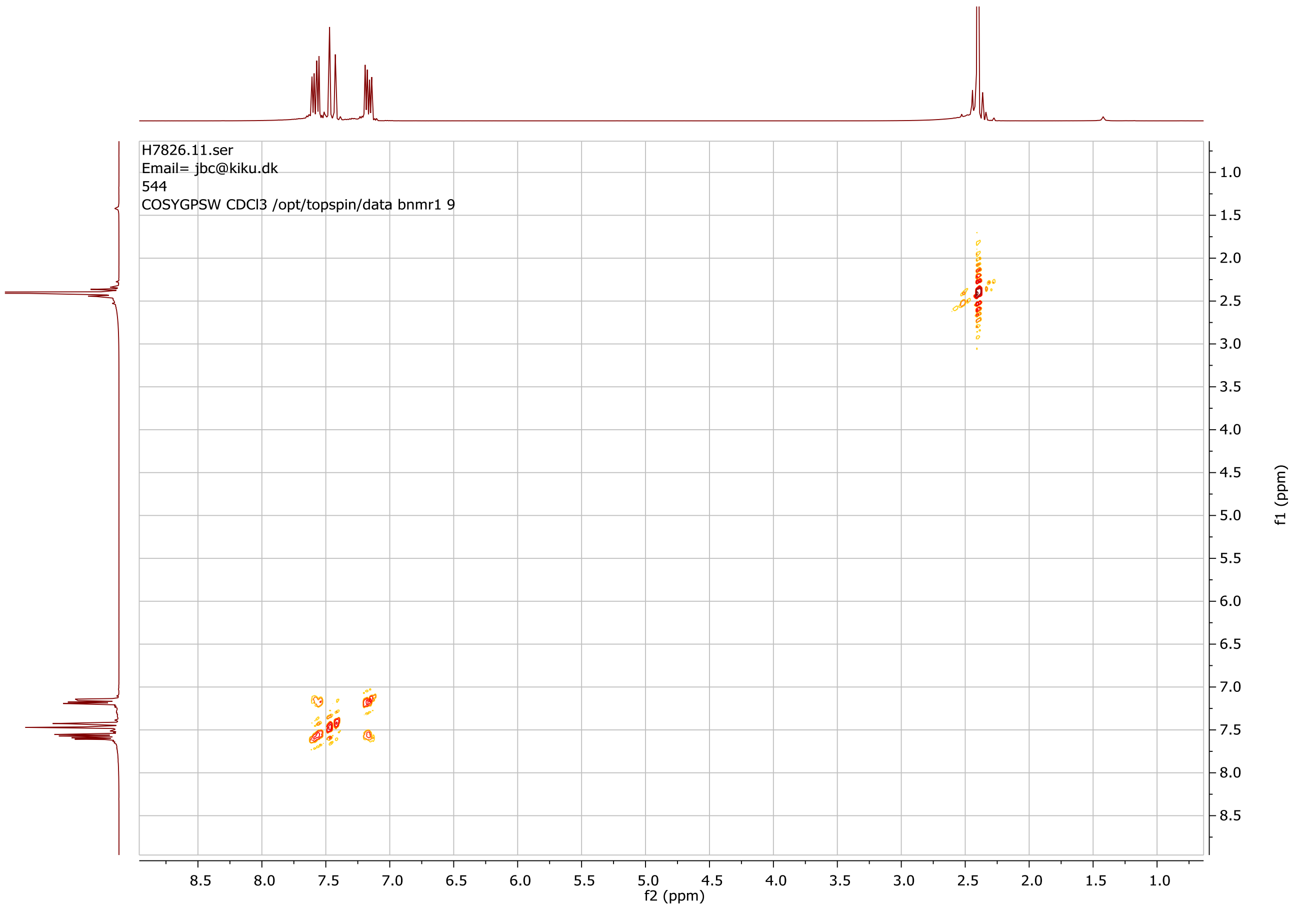

H7826.11.ser
Email= jbc@kiku.dk
544
COSYGPSW CDCl3 /opt/topspin/data bnmr1 9
8.5
8.0
7.5
7.0
6.5
6.0
5.5
5.0
4.5
4.0
3.5
3.0
2.5
2.0
1.5
1.0
f2 (ppm)
f1 (ppm)

^{13}C-NMR:

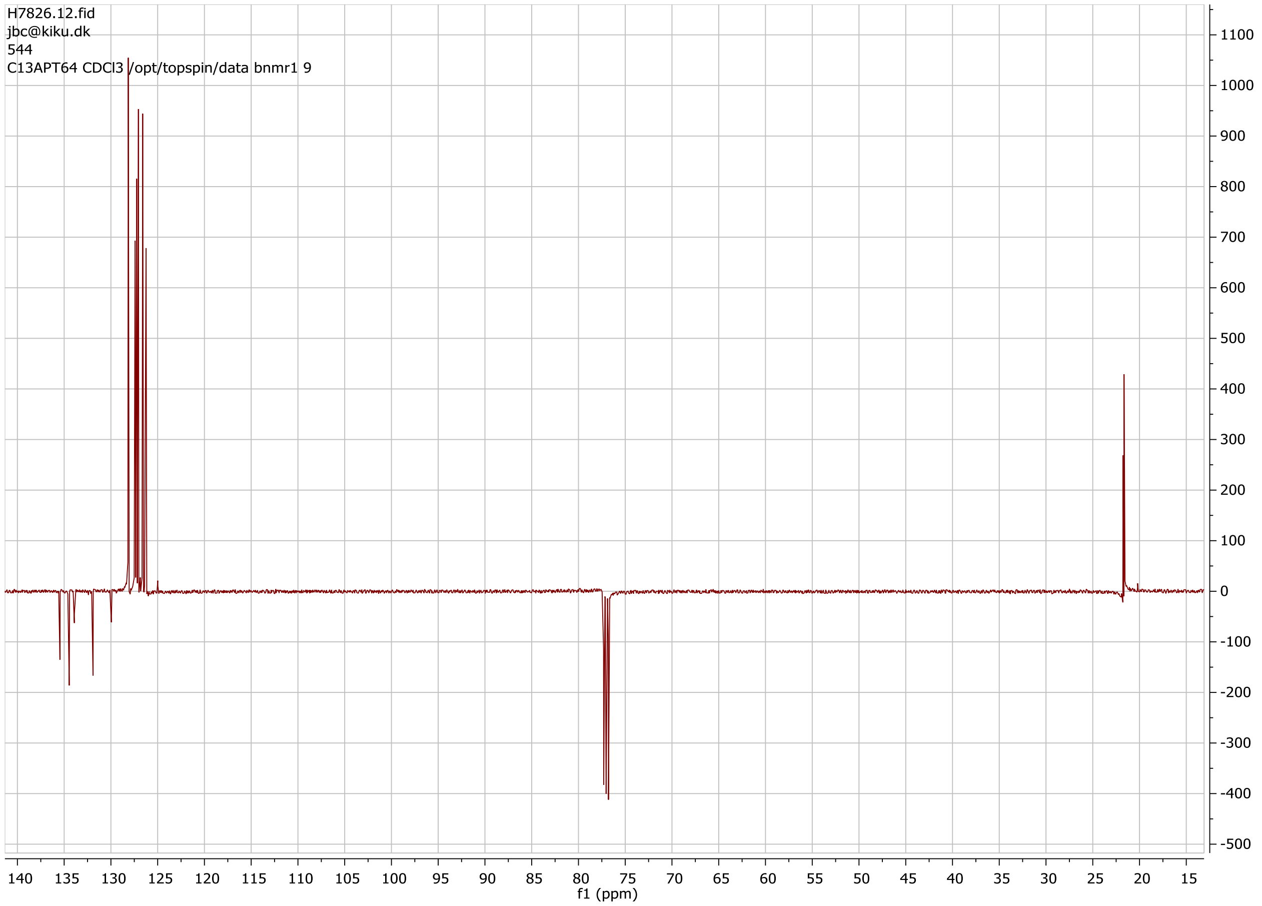
H7826.12.fid
jbc@kiku.dk
544
C13APT64 CDCl3 /opt/topspin/data bnmr1 9
f1 (ppm)

HSQC-NMR:

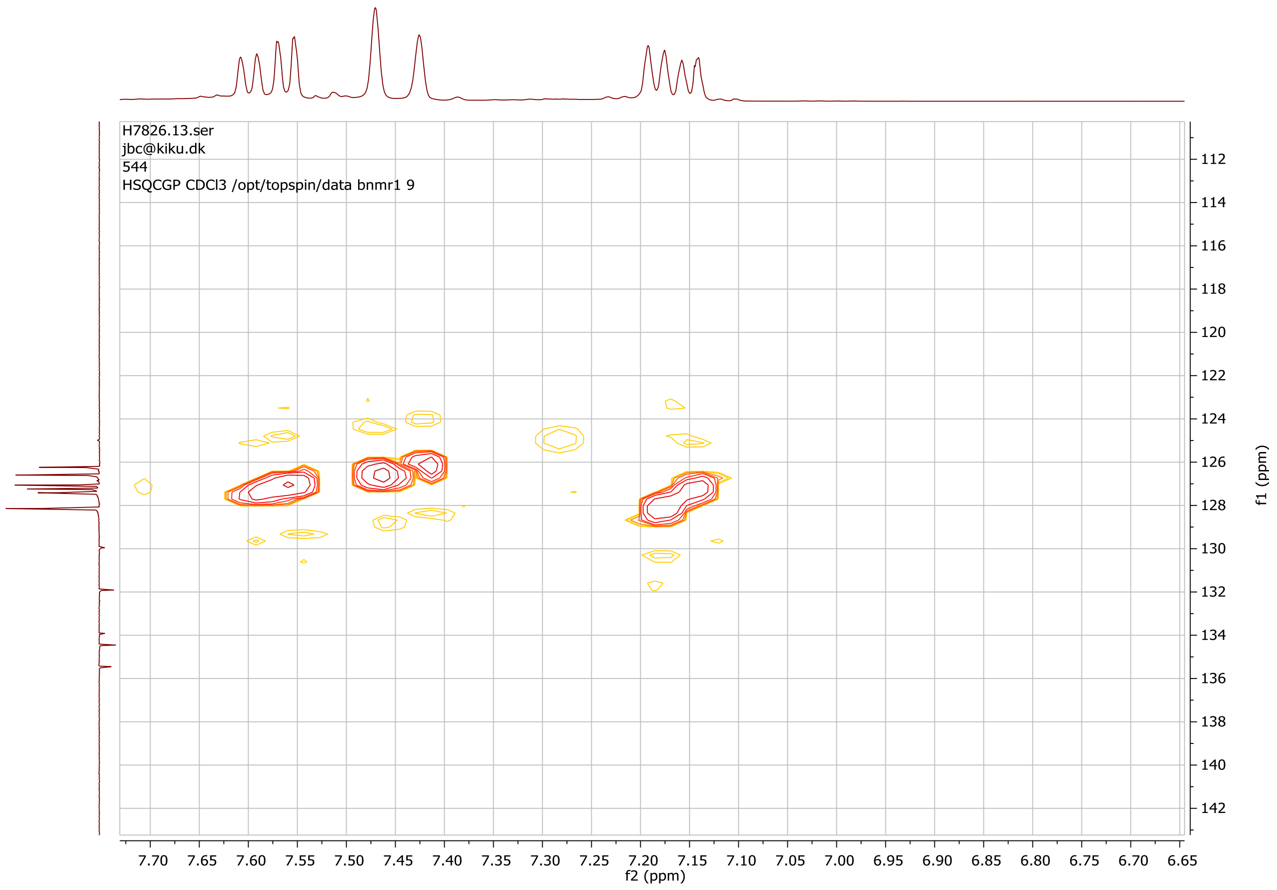
H7826.13.ser
jbc@kiku.dk
544
HSQCGP CDCl3 /opt/topspin/data bnmr1 9
f2 (ppm)
f1 (ppm)

HSQC-spectrum of the mixture of 2,6- and 2,7-DMN in $CDCl_3$. Finding H1/H1' in 2,6-DMN:

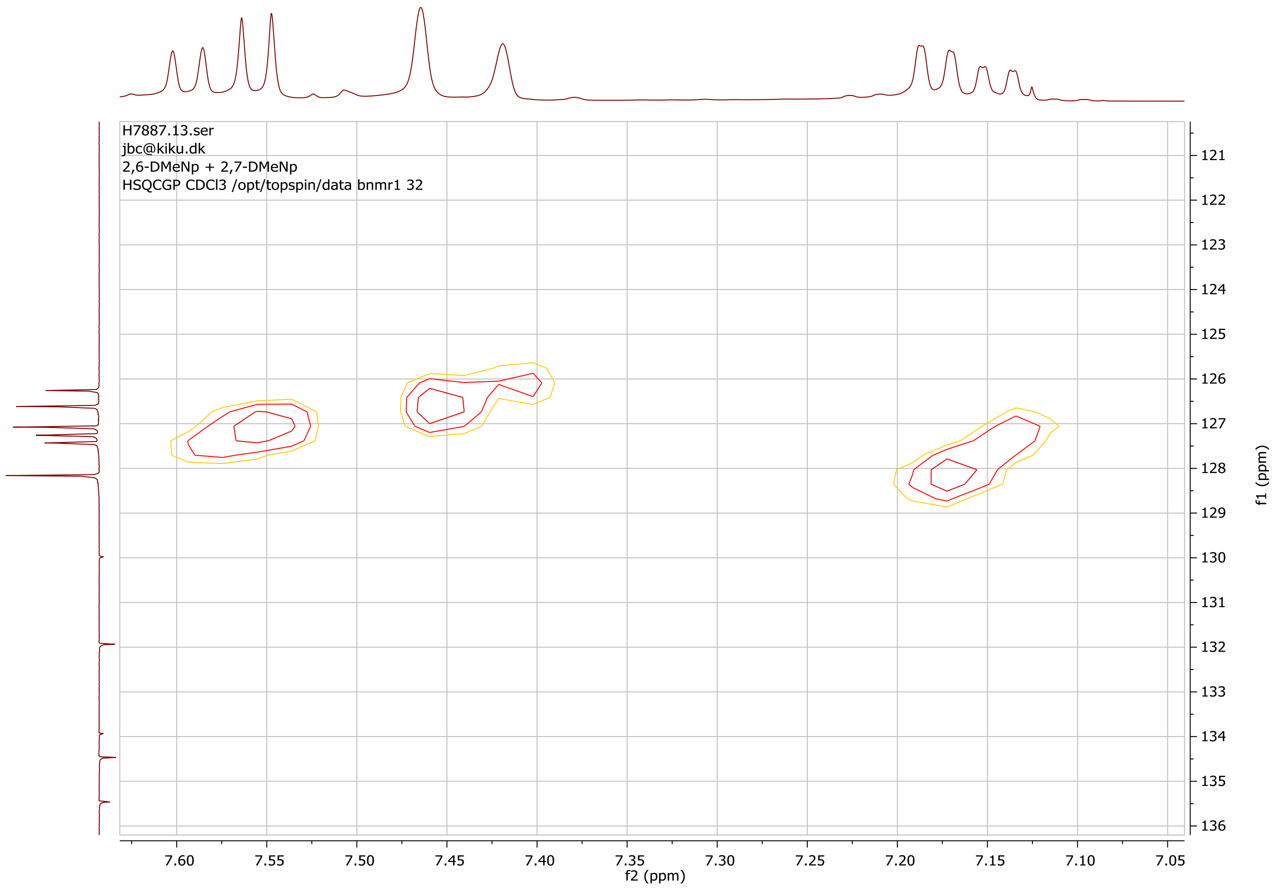

The peak at 7.46 ppm corresponds to H1/H1' in 2,6-DMN and the peak at 7,42 ppm corresponds to H1/H1' in 2,7-DMN.

Determining the composition:

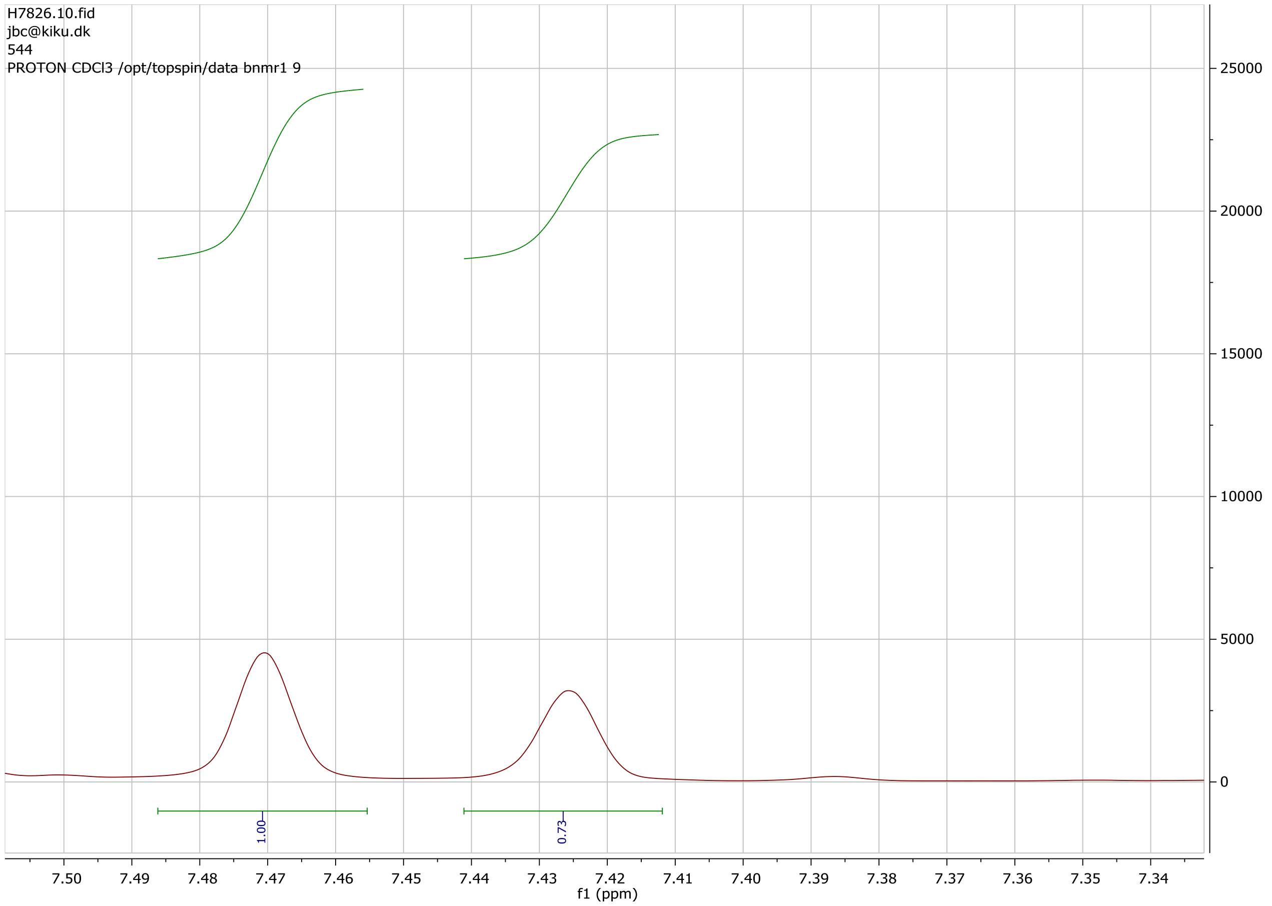

Gives a 1:0.73 ratio between 2,6- and 2,7-DMN corresponding to 58 % 2,6-DMN in the mixture.

NMR-spectra of compound **4 in $CDCl_3$**

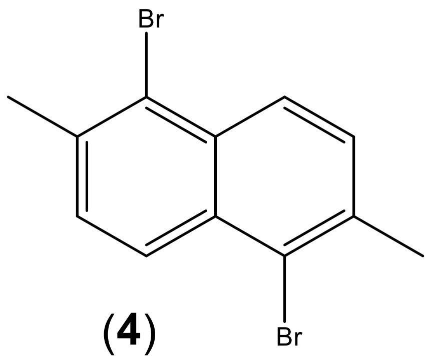

(4)

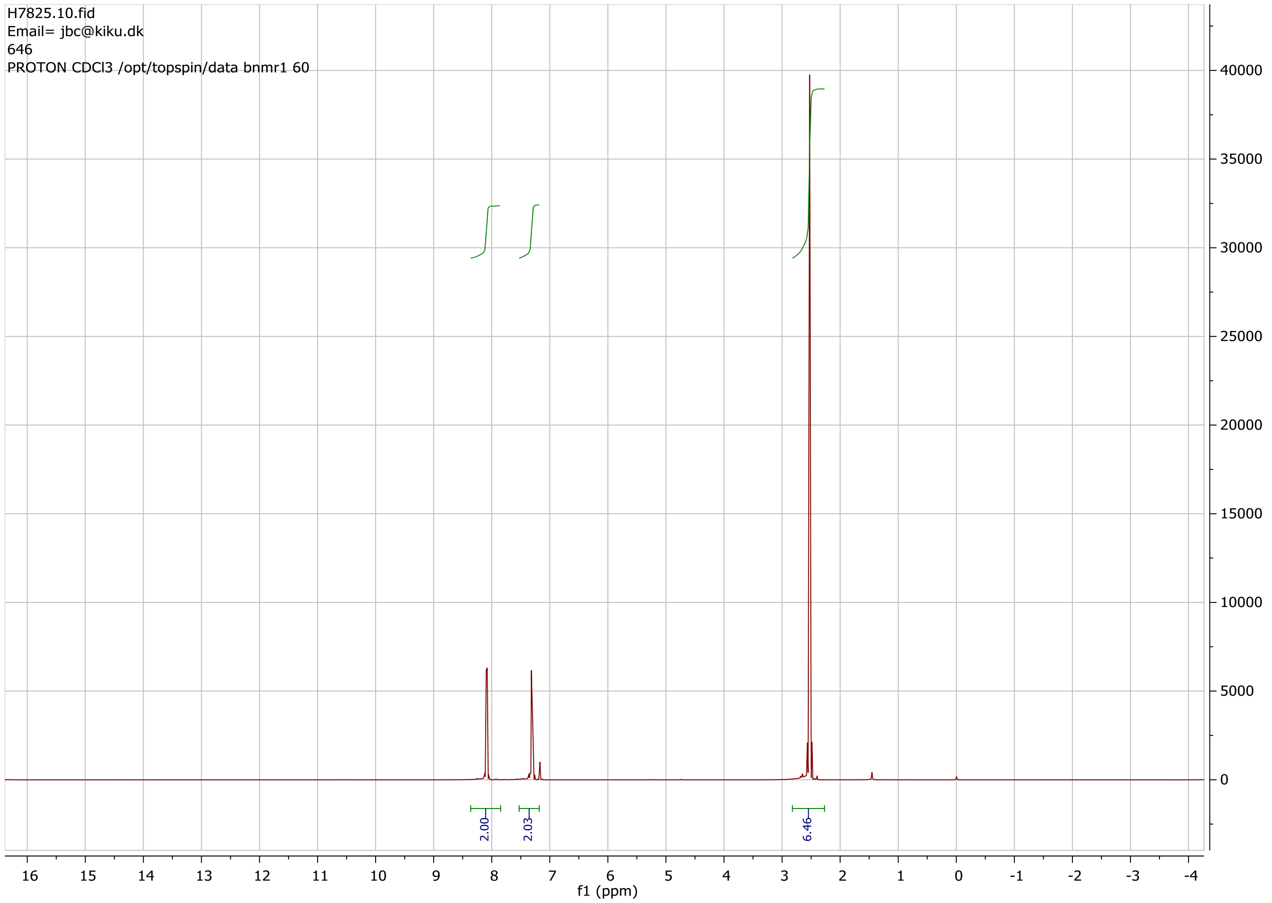

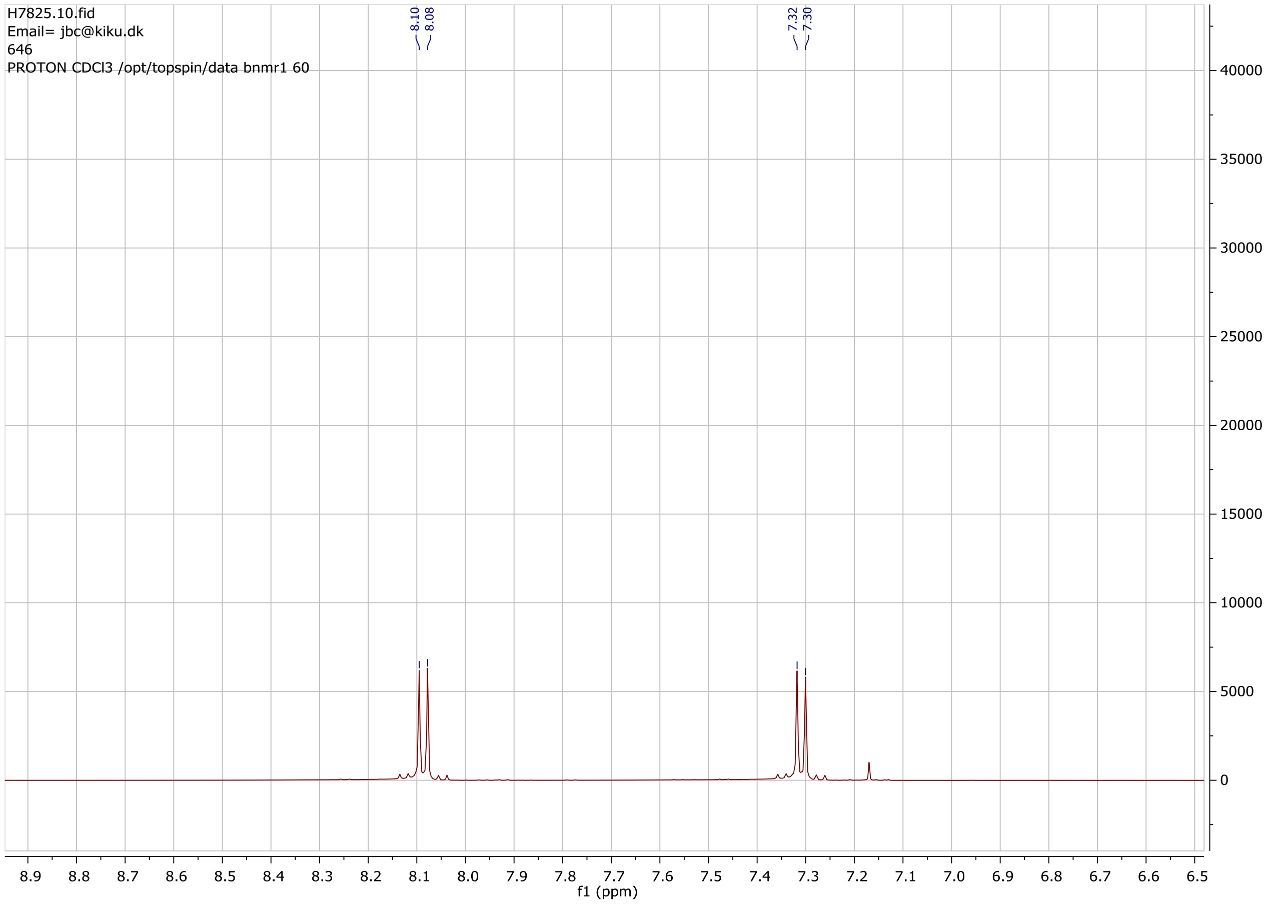
H7825.10.fid
Email= jbc@kiku.dk
646
PROTON CDCl3 /opt/topspin/data bnmr1 60
8.10
8.08
7.32
7.30
40000
35000
30000
25000
20000
15000
10000
5000
0
8.9 8.8 8.7 8.6 8.5 8.4 8.3 8.2 8.1 8.0 7.9 7.8 7.7 7.6 7.5 7.4 7.3 7.2 7.1 7.0 6.9 6.8 6.7 6.6 6.5
f1 (ppm)

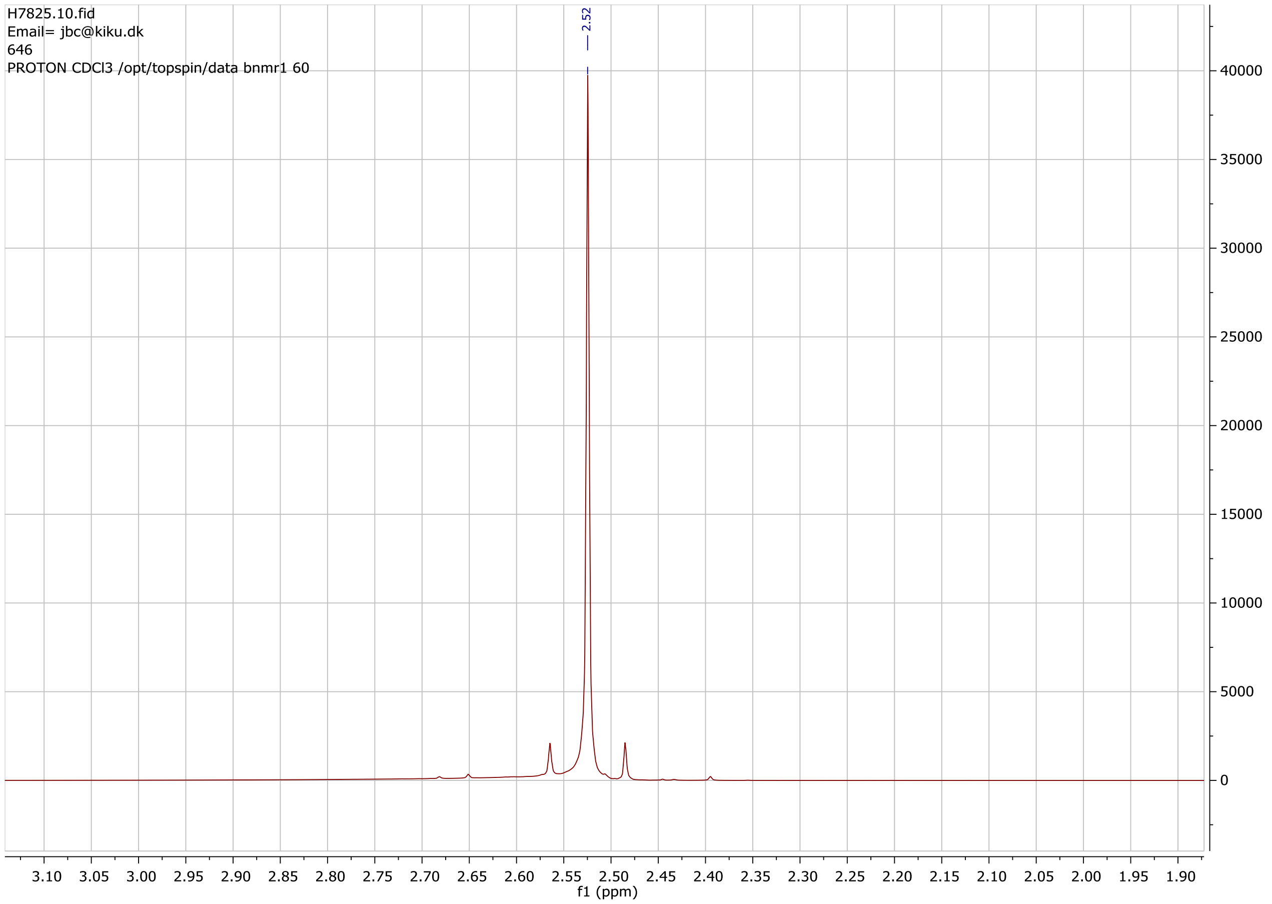
H7825.10.fid
Email= jbc@kiku.dk
646
PROTON CDCl3 /opt/topspin/data bnmr1 60
2.52
40000
35000
30000
25000
20000
15000
10000
5000
0
3.10 3.05 3.00 2.95 2.90 2.85 2.80 2.75 2.70 2.65 2.60 2.55 2.50 2.45 2.40 2.35 2.30 2.25 2.20 2.15 2.10 2.05 2.00 1.95 1.90
f1 (ppm)

COSY

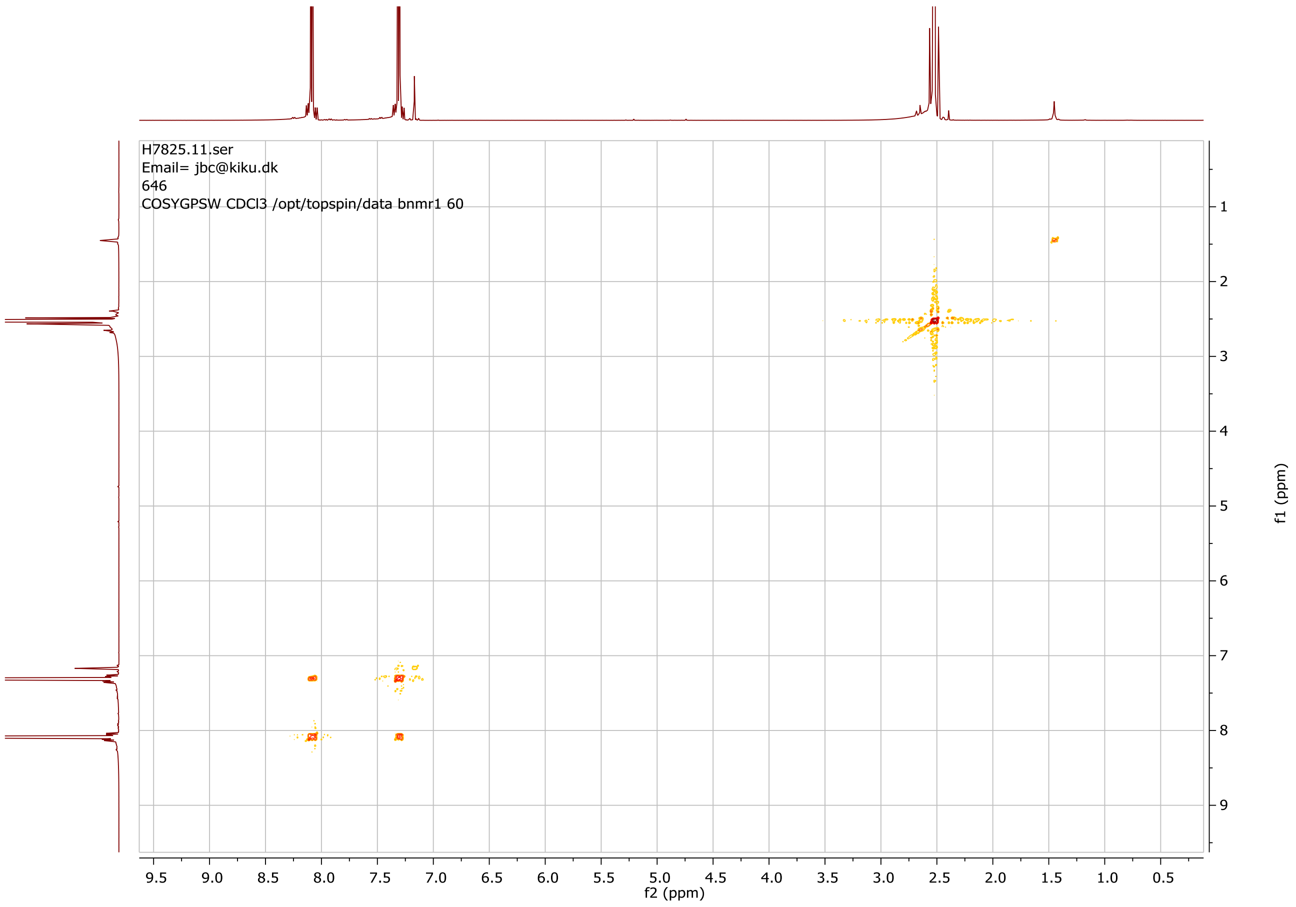

H7825.11.ser
Email= jbc@kiku.dk
646
COSYGPSW CDCl3 /opt/topspin/data bnmr1 60
9.5 9.0 8.5 8.0 7.5 7.0 6.5 6.0 5.5 5.0 4.5 4.0 3.5 3.0 2.5 2.0 1.5 1.0 0.5
f2 (ppm)
1 2 3 4 5 6 7 8 9
f1 (ppm)

APC-^{13}C

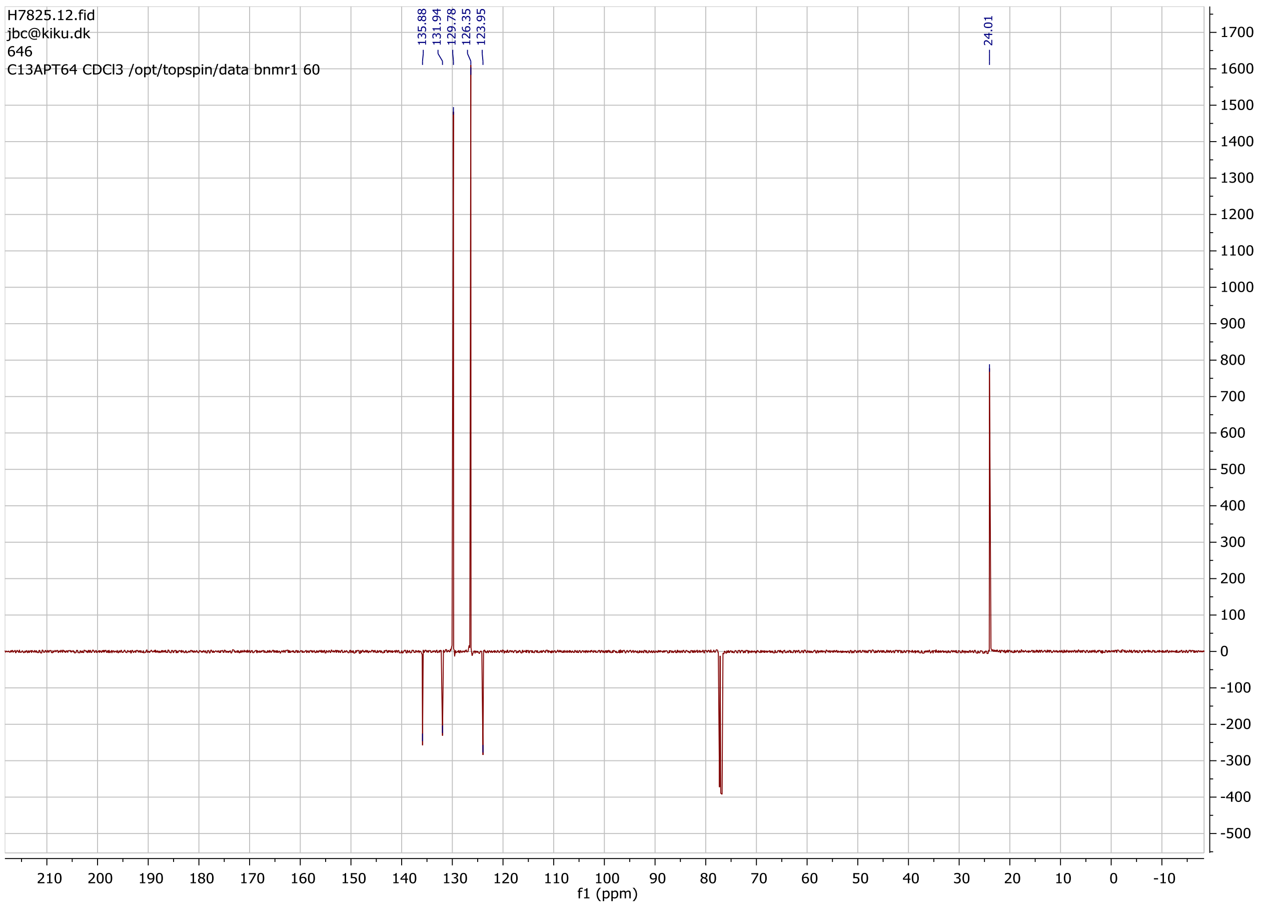
H7825.12.fid
jbc@kiku.dk
646
C13APT64 CDCl3 /opt/topspin/data bnmr1 60
135.88
131.94
129.78
126.35
123.95
24.01
f1 (ppm)

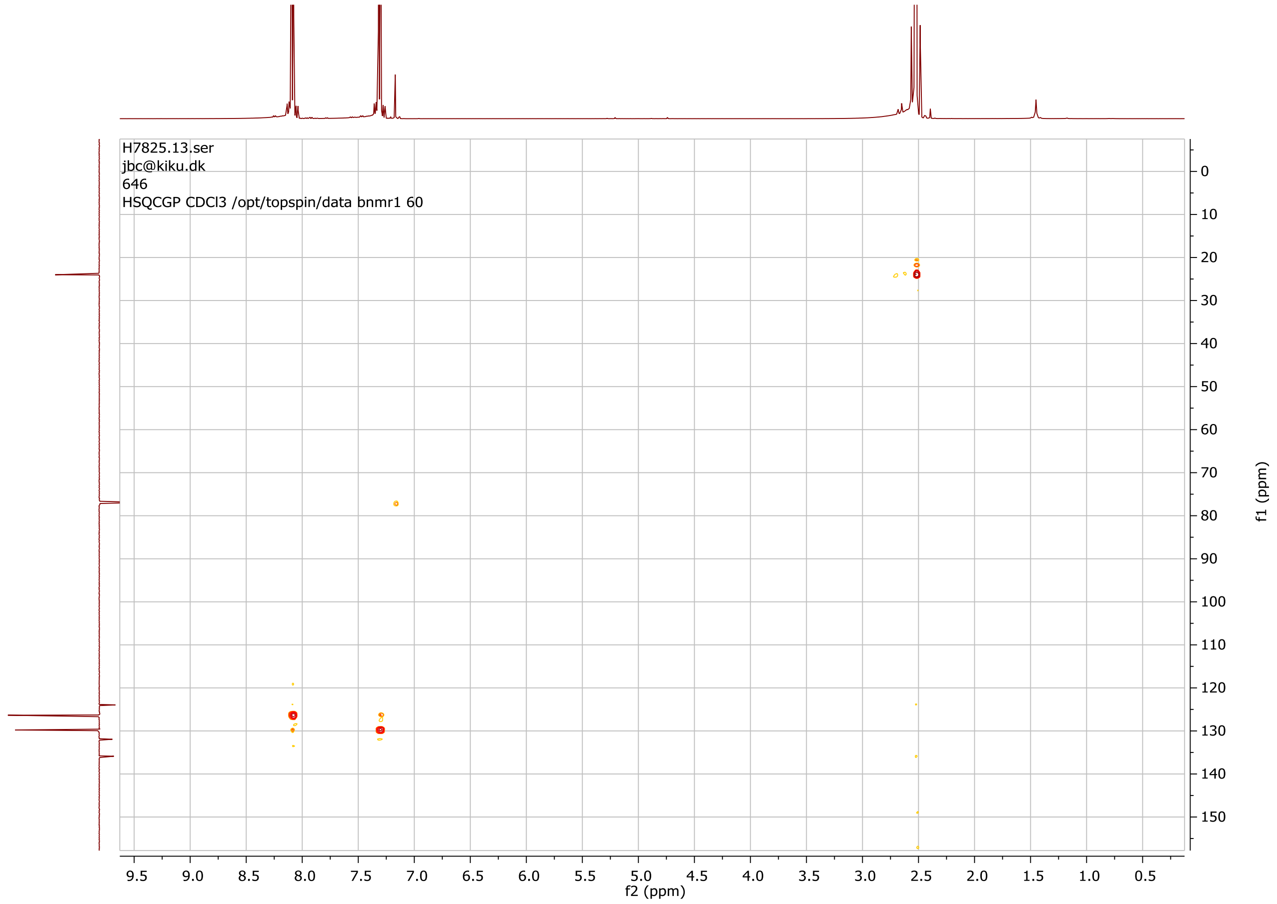
H7825.13.ser
jbc@kiku.dk
646
HSQCGP CDCl3 /opt/topspin/data bnmr1 60
f2 (ppm)
f1 (ppm)

HSQC-NMR

NMR-spectra of compound **5 in $CDCl_3$**

^{1}H-NMR

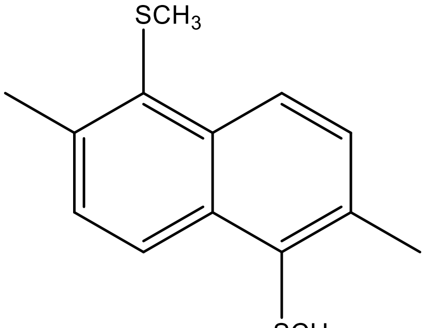

(**5**)

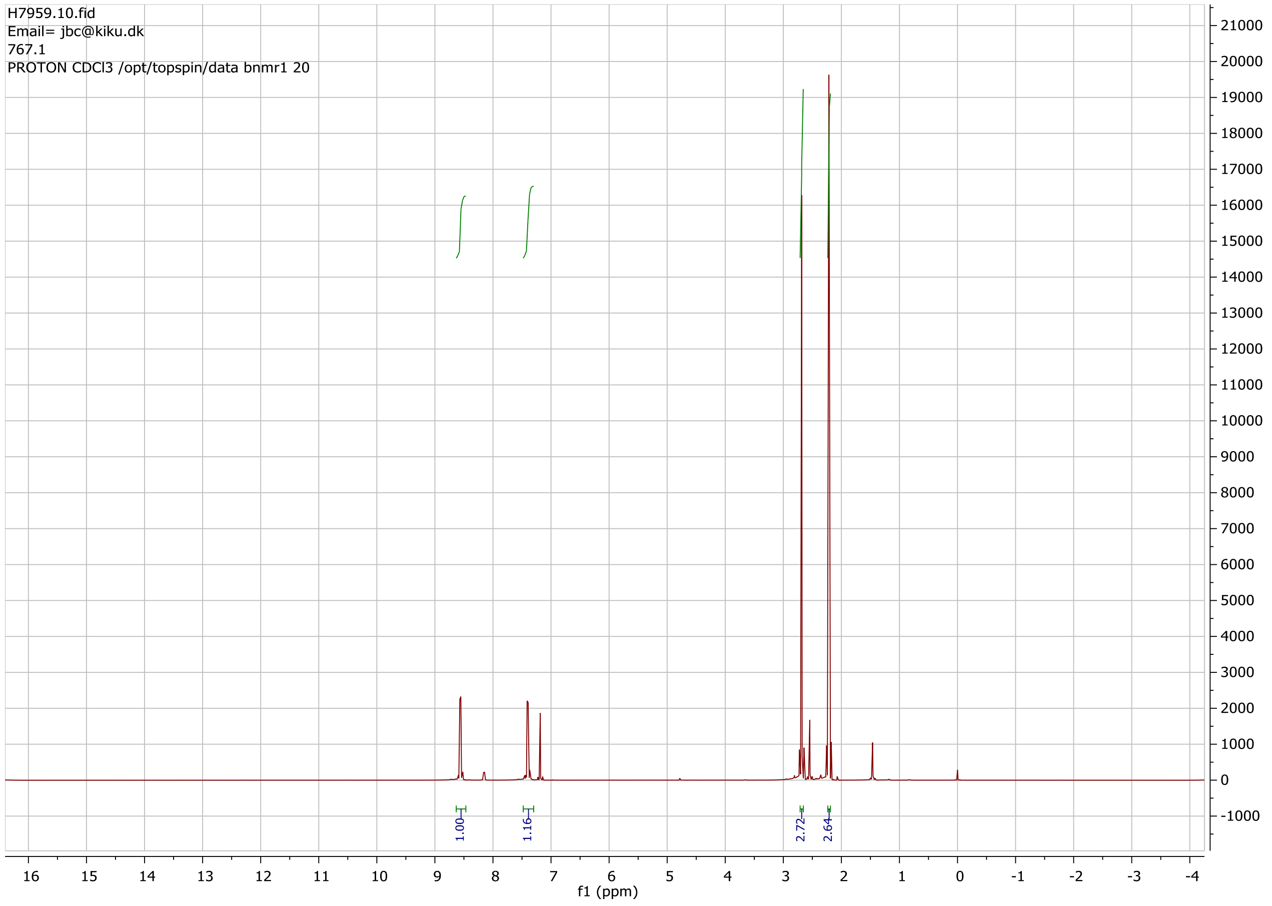

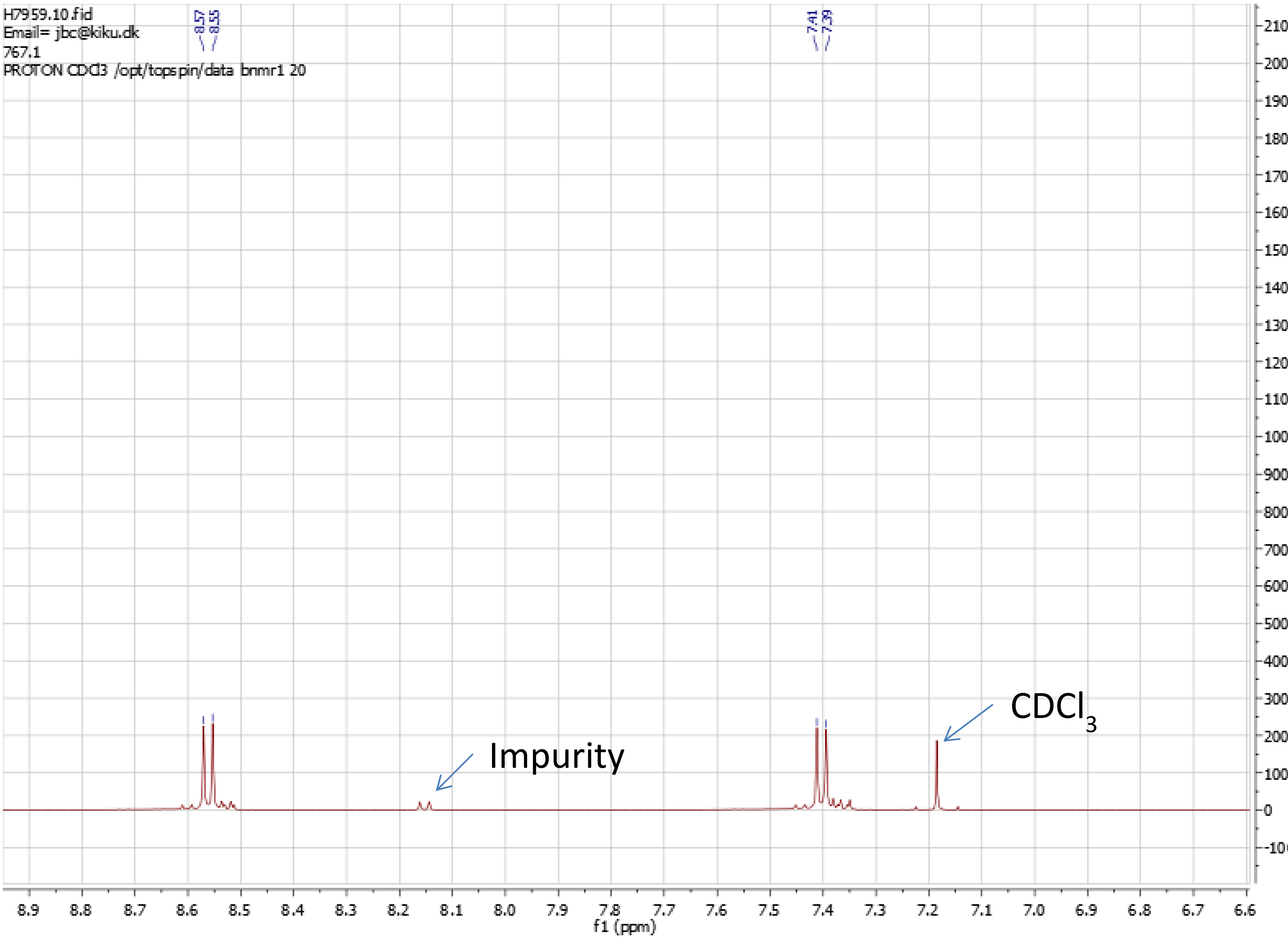

H7959.10.fid
Email= jbc@kiku.dk
767.1
PROTON CDCl3 /opt/topspin/data bnmr1 20
8.57
8.55
7.41
7.39
Impurity
CDCl3
f1 (ppm)

COSY-NMR

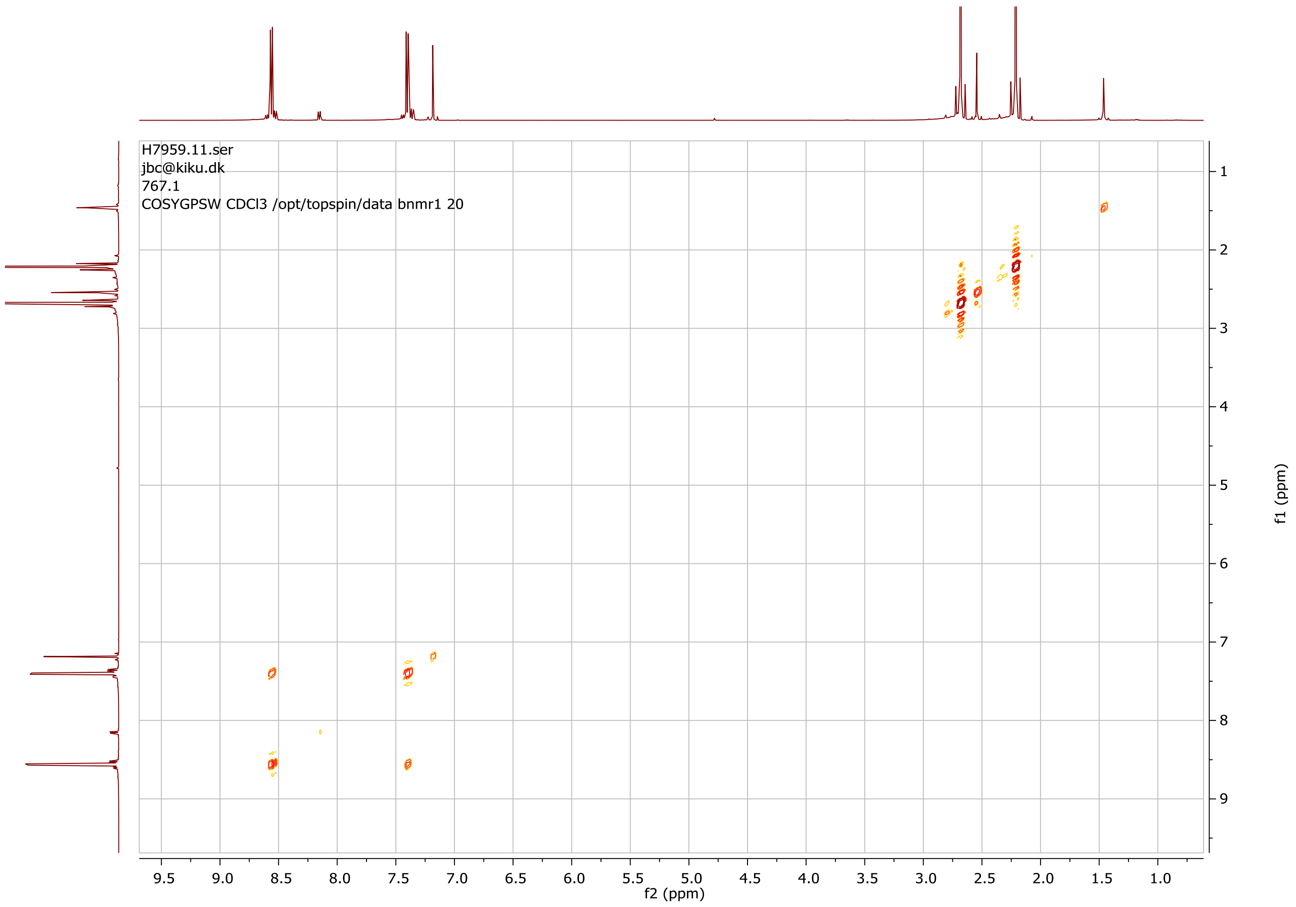
H7959.11.ser
jbc@kiku.dk
767.1
COSYGPSW CDCl3 /opt/topspin/data bnmr1 20
f2 (ppm)
f1 (ppm)

APT-^{13}C-NMR

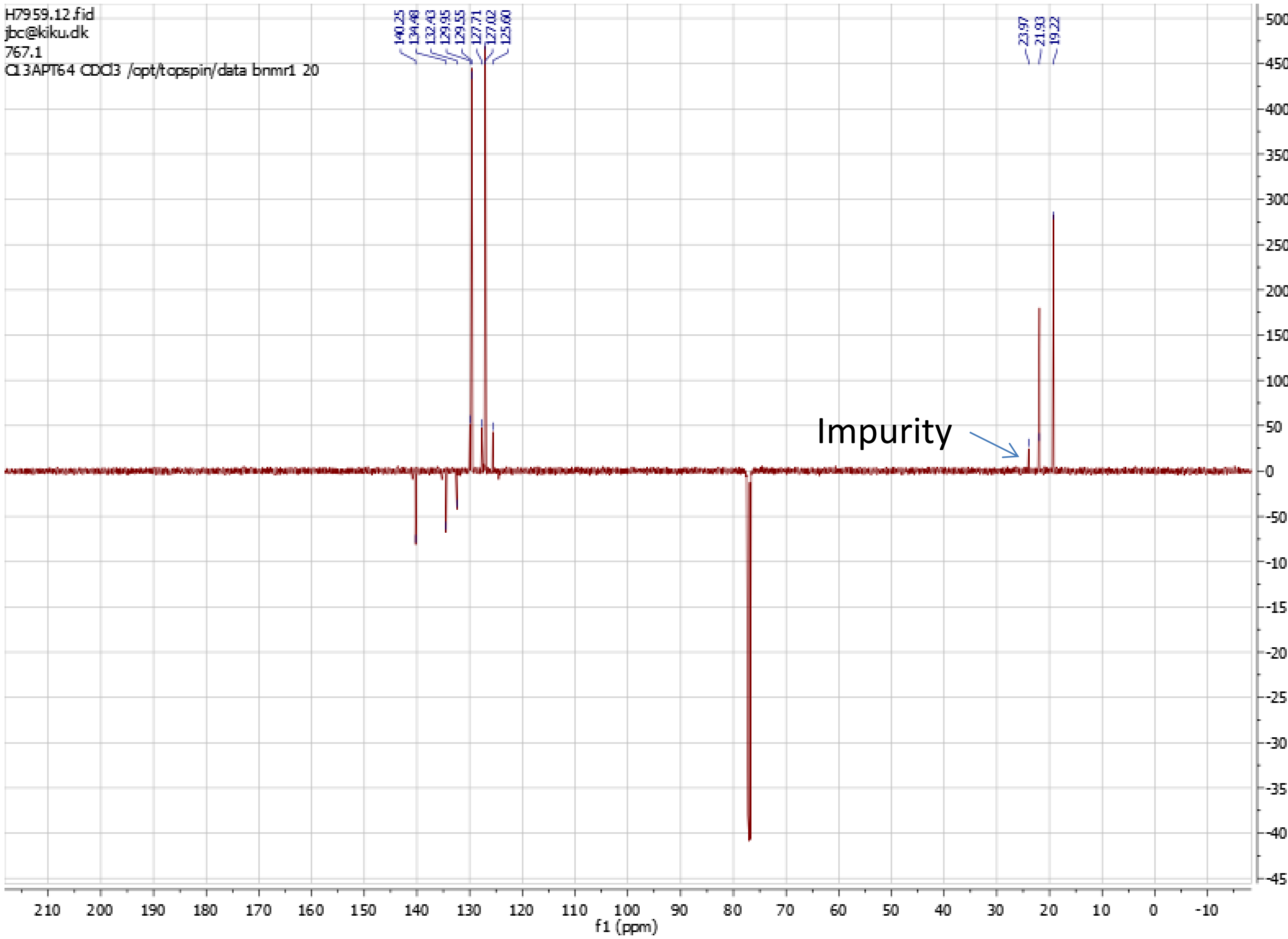
H7959.12.fid
jbc@kiku.dk
767.1
C13APT64 CDCl3 /opt/topspin/data bnmr1 20
140.25
134.48
132.43
129.95
129.55
127.71
127.02
125.60
23.97
21.93
19.22
Impurity
f1 (ppm)

HSQC-NMR

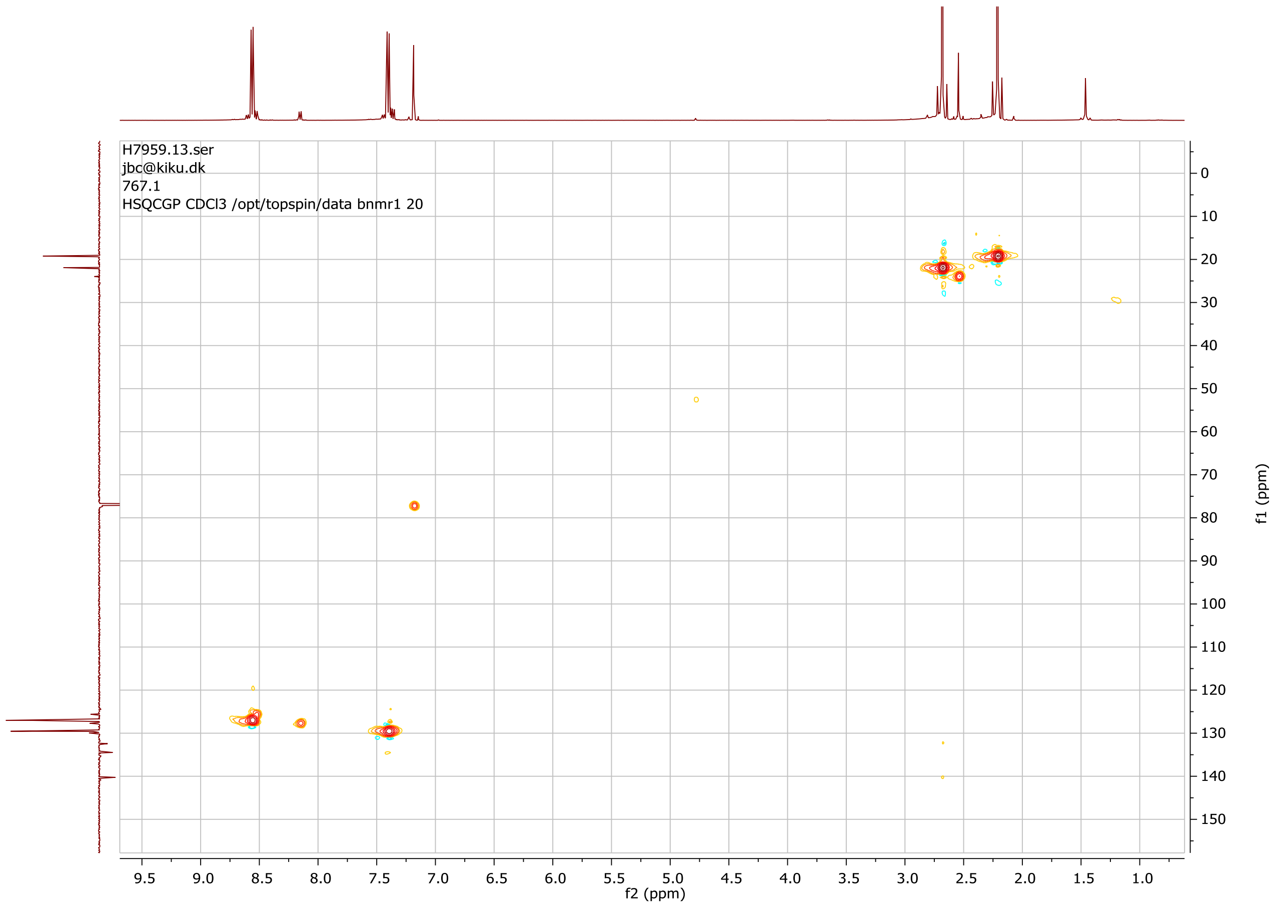

H7959.13.ser
jbc@kiku.dk
767.1
HSQCGP CDCl3 /opt/topspin/data bnmr1 20
9.5
9.0
8.5
8.0
7.5
7.0
6.5
6.0
5.5
5.0
4.5
4.0
3.5
3.0
2.5
2.0
1.5
1.0
f2 (ppm)
0
10
20
30
40
50
60
70
80
90
100
110
120
130
140
150
f1 (ppm)

NMR-spectra of compound **6** in $CDCl_3$

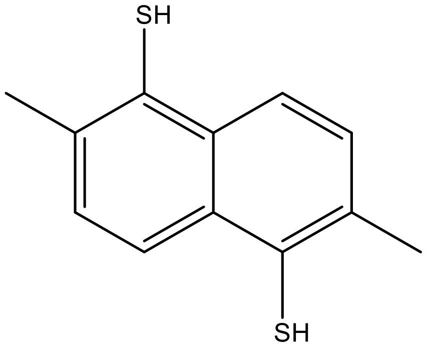

(**6**)

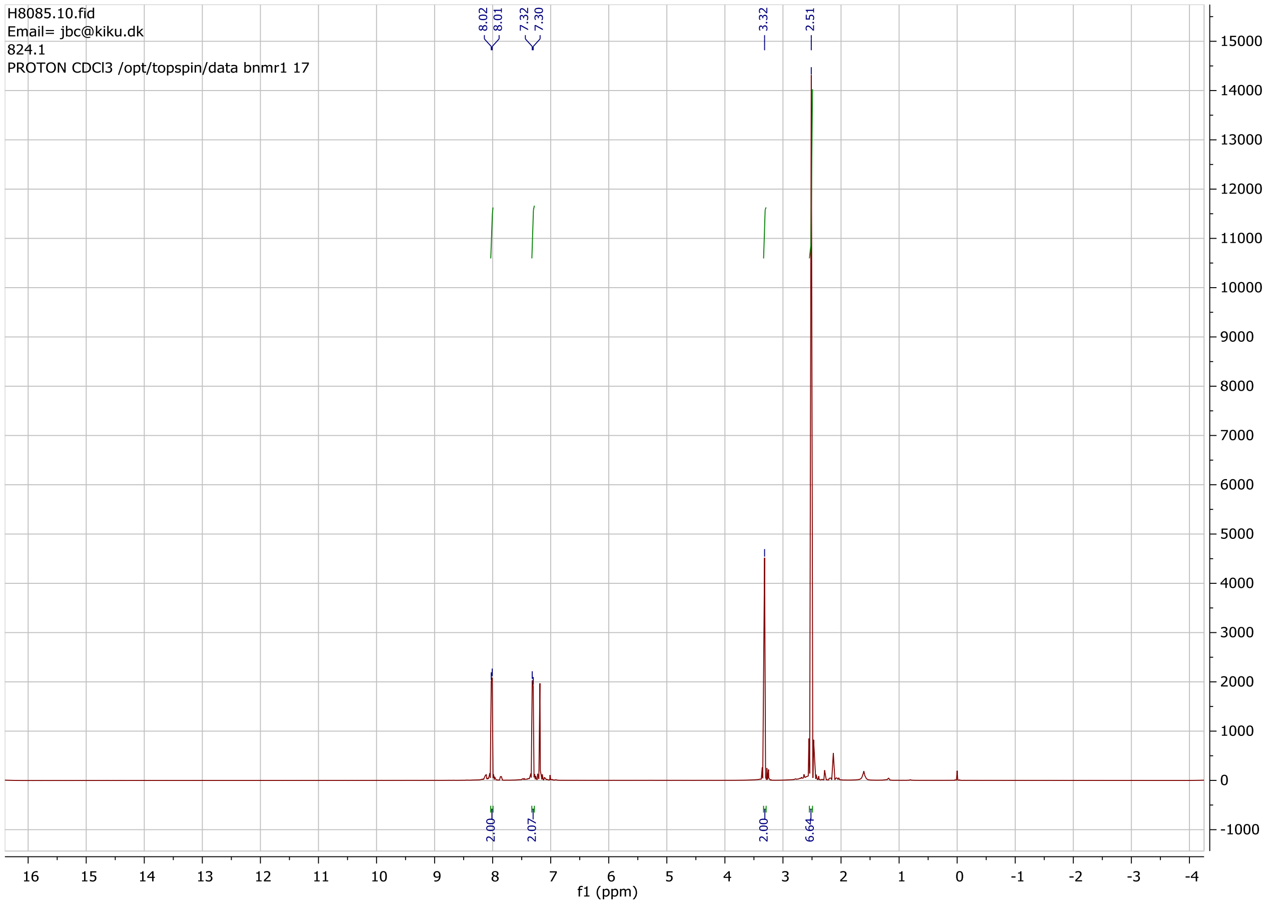

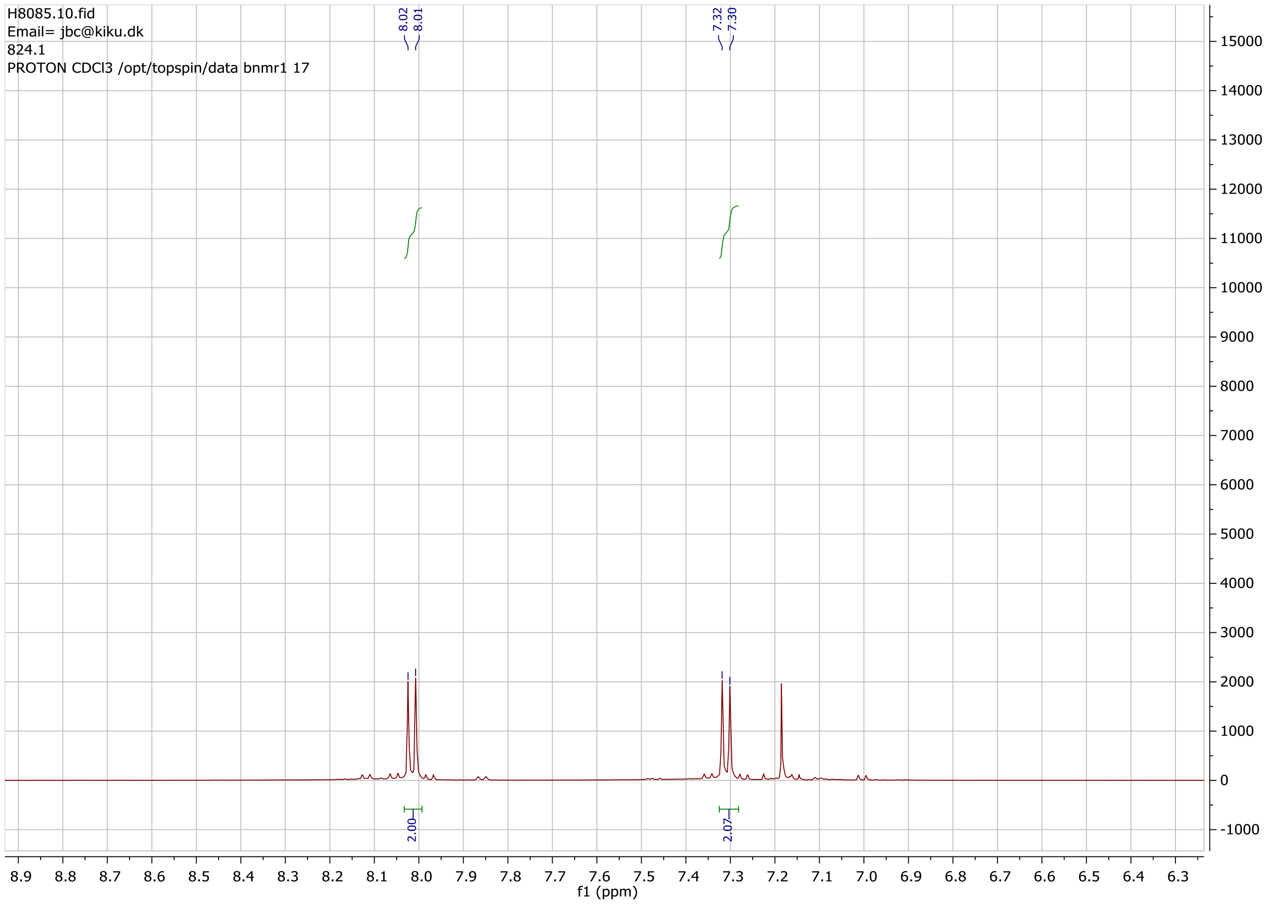
H8085.10.fid
Email= jbc@kiku.dk
824.1
PROTON CDCl3 /opt/topspin/data bnmr1 17
8.02
8.01
7.32
7.30
2.00
2.07
f1 (ppm)

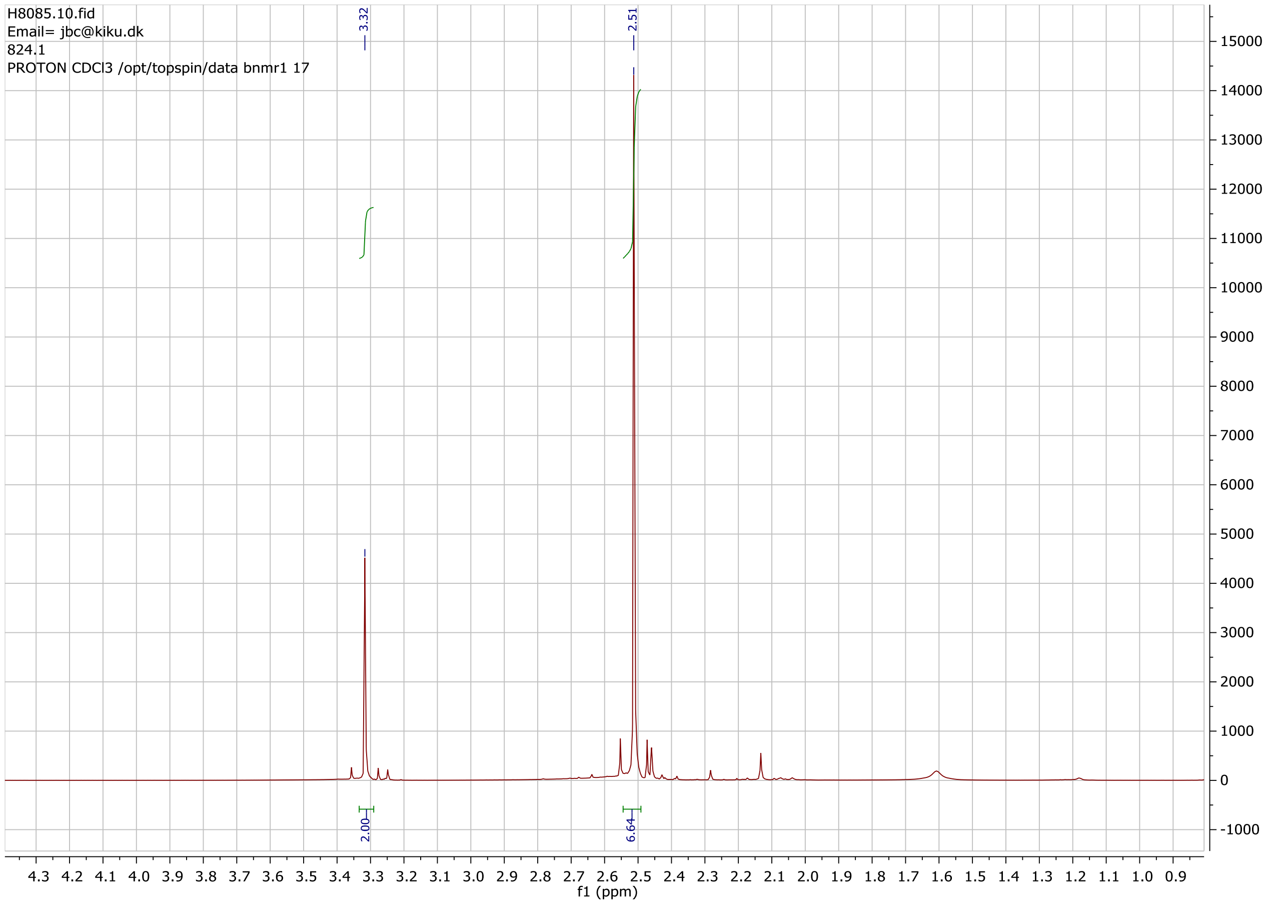
H8085.10.fid
Email= jbc@kiku.dk
824.1
PROTON CDCl3 /opt/topspin/data bnmr1 17
3.32
2.51
2.00
6.64
f1 (ppm)

COSY-NMR

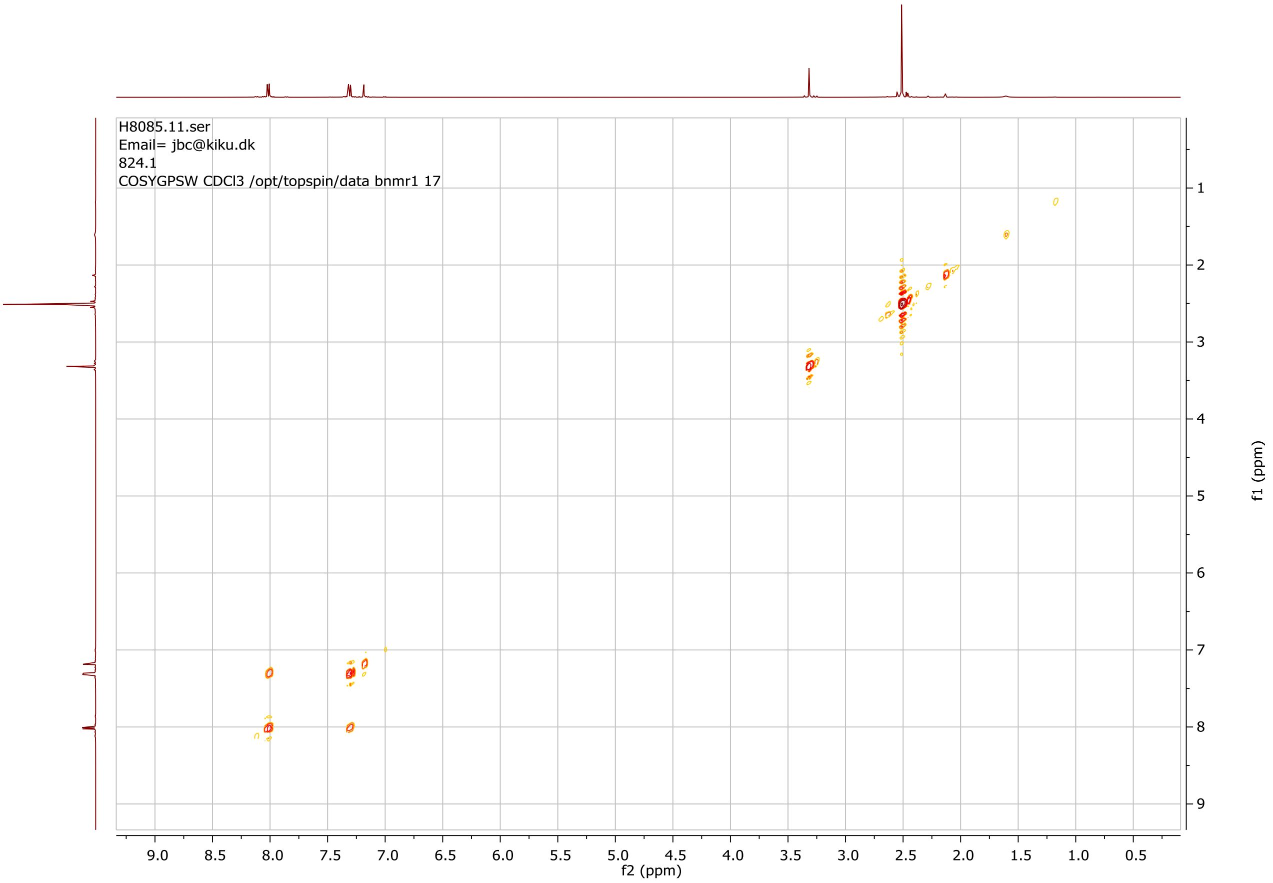
H8085.11.ser
Email= jbc@kiku.dk
824.1
COSYGPSW CDCl3 /opt/topspin/data bnmr1 17
9.0 8.5 8.0 7.5 7.0 6.5 6.0 5.5 5.0 4.5 4.0 3.5 3.0 2.5 2.0 1.5 1.0 0.5
f2 (ppm)
1 2 3 4 5 6 7 8 9
f1 (ppm)

APT-^{13}C-NMR

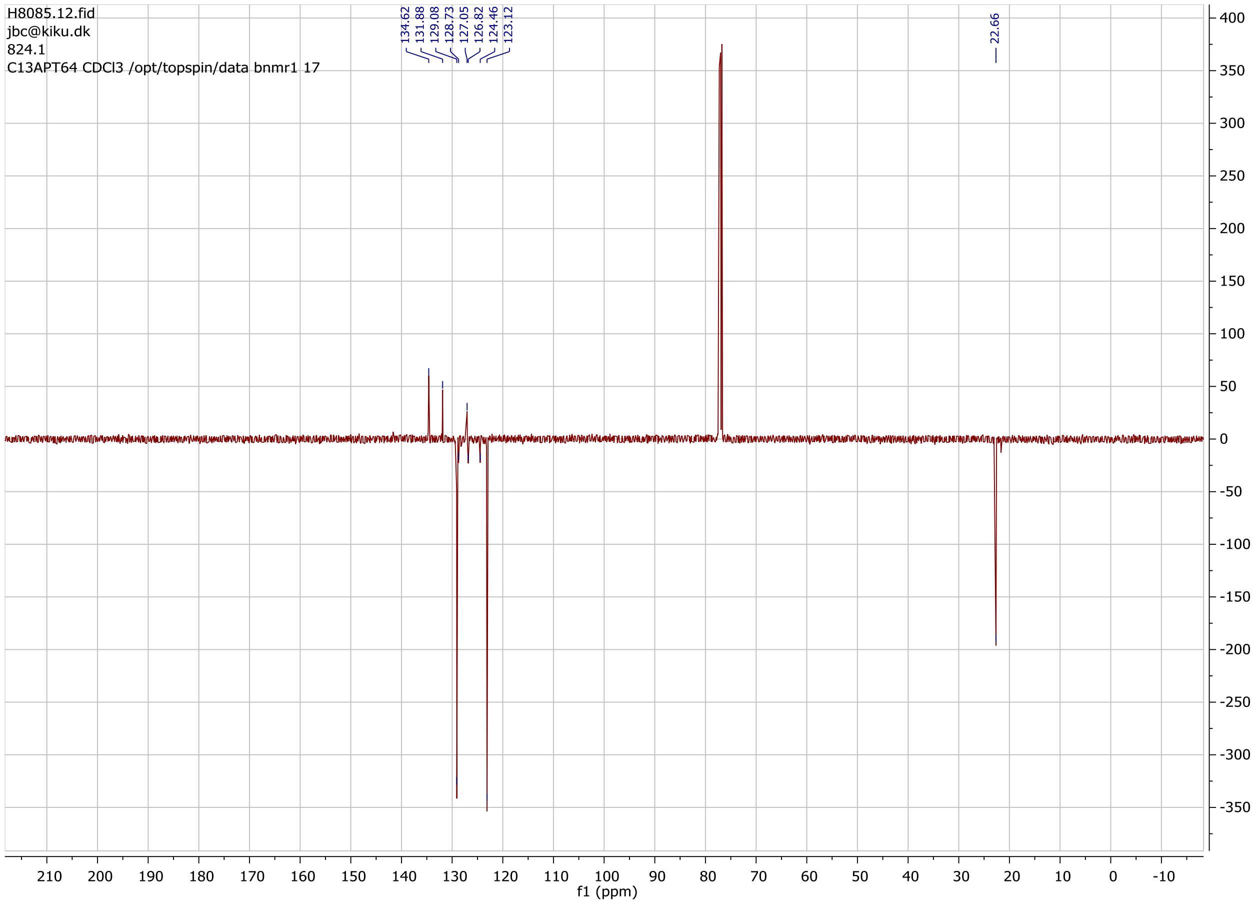

H8085.12.fid
jbc@kiku.dk
824.1
C13APT64 CDCl3 /opt/topspin/data bnmr1 17
134.62
131.88
129.08
128.73
127.05
126.82
124.46
123.12
22.66
f1 (ppm)

HSQC-NMR

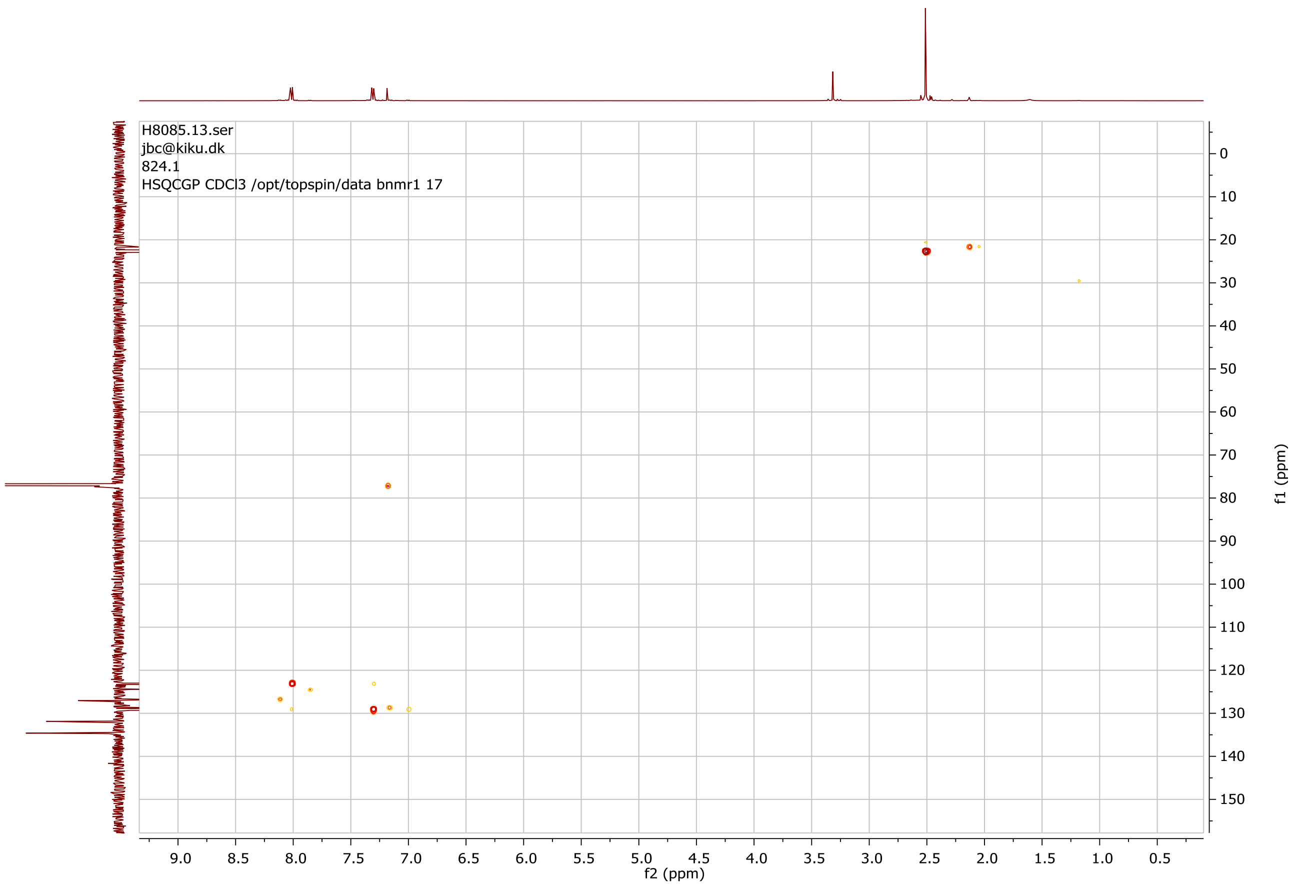

H8085.13.ser
jbc@kiku.dk
824.1
HSQCGP CDCl3 /opt/topspin/data bnmr1 17
f2 (ppm)
f1 (ppm)

NMR of compound **7** in $CDCl_3$

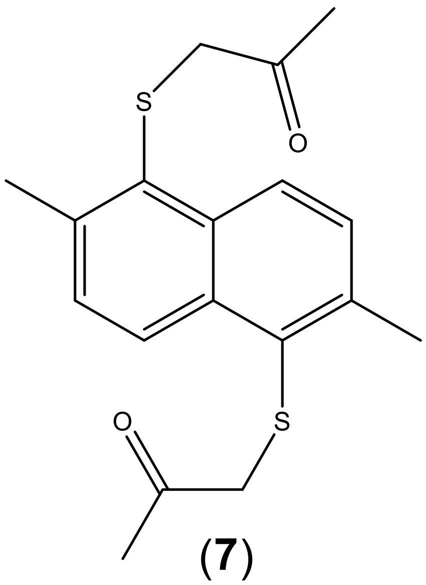

(7)

^{1}H-NMR

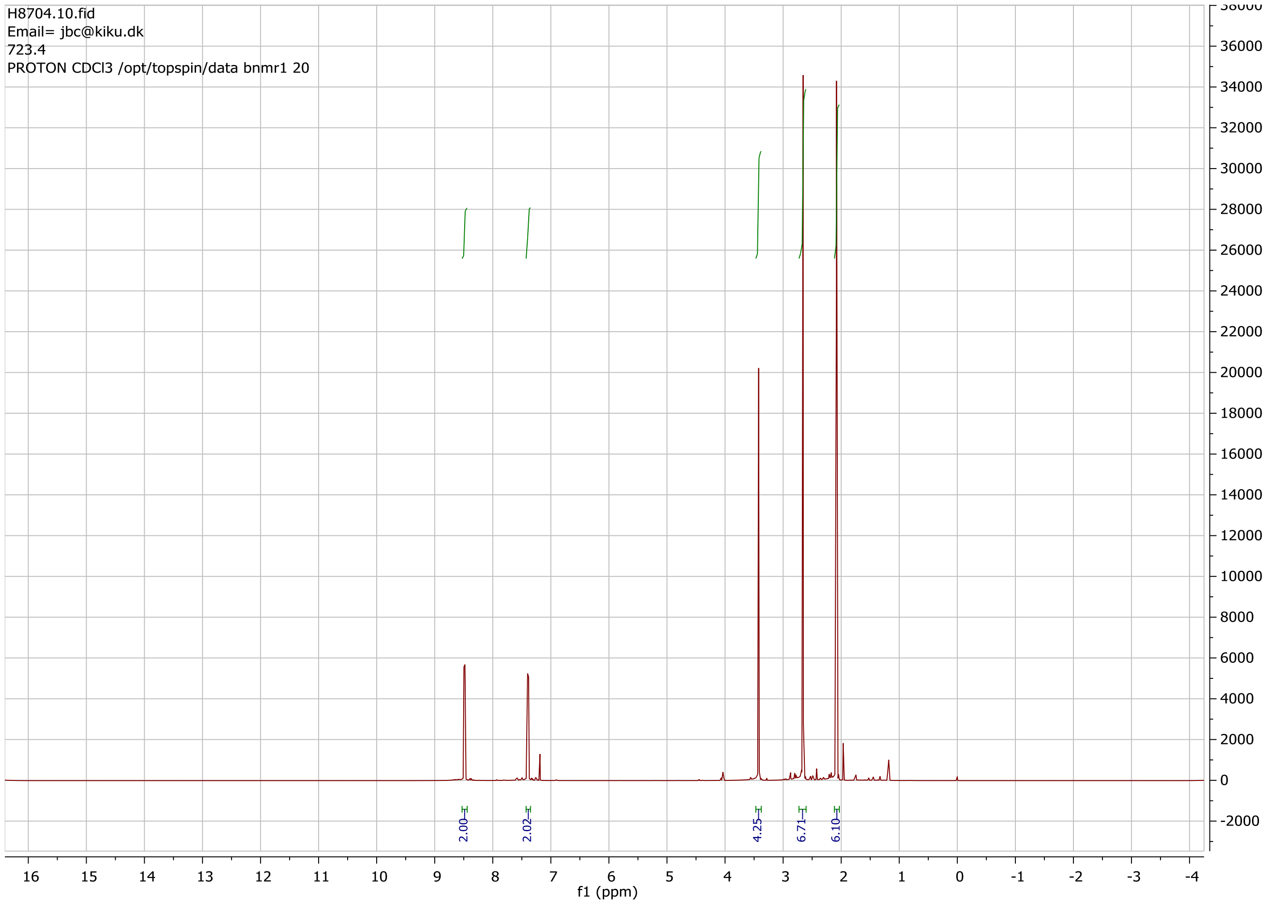

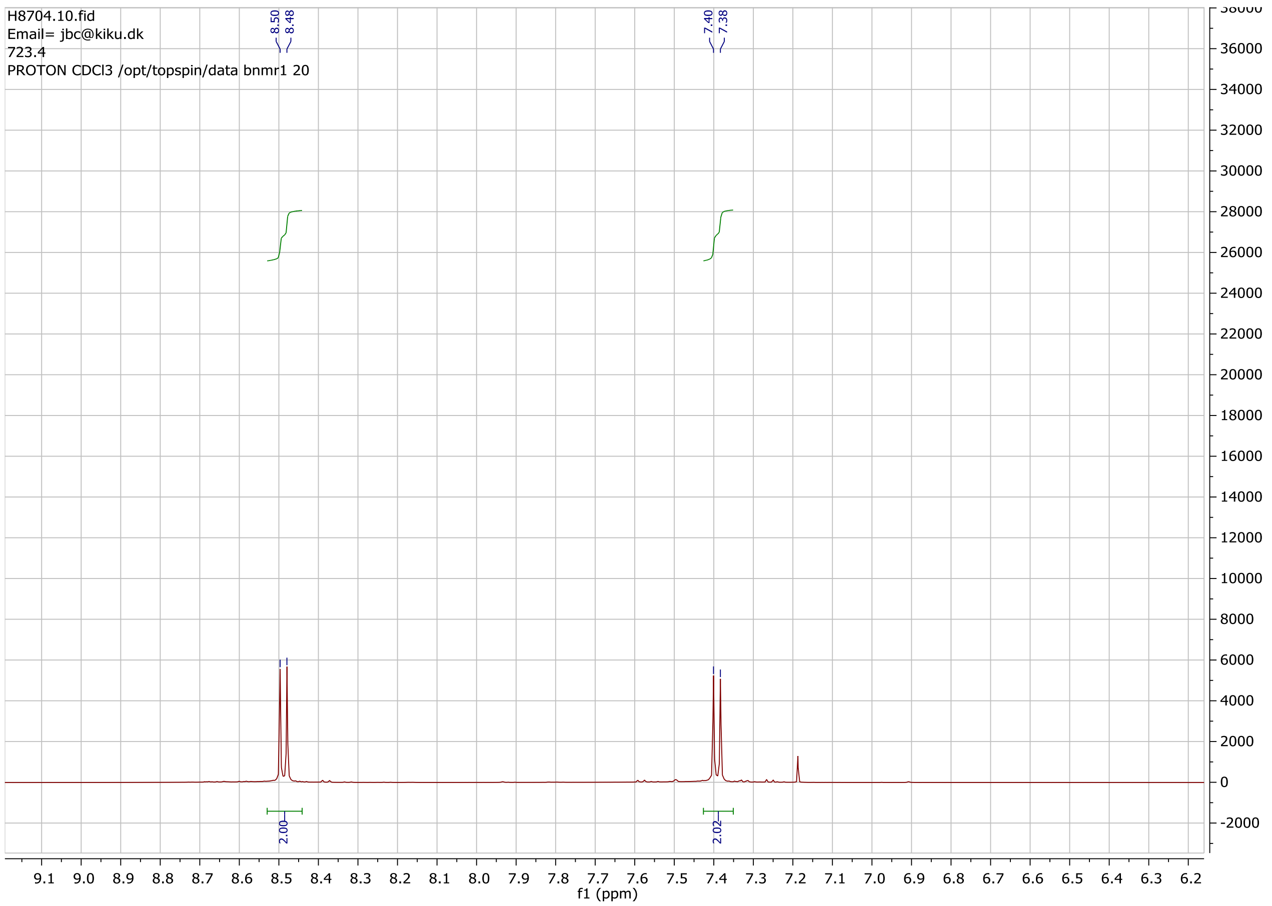
H8704.10.fid
Email= jbc@kiku.dk
723.4
PROTON CDCl3 /opt/topspin/data bnmr1 20
8.50
8.48
7.40
7.38
2.00
2.02
f1 (ppm)

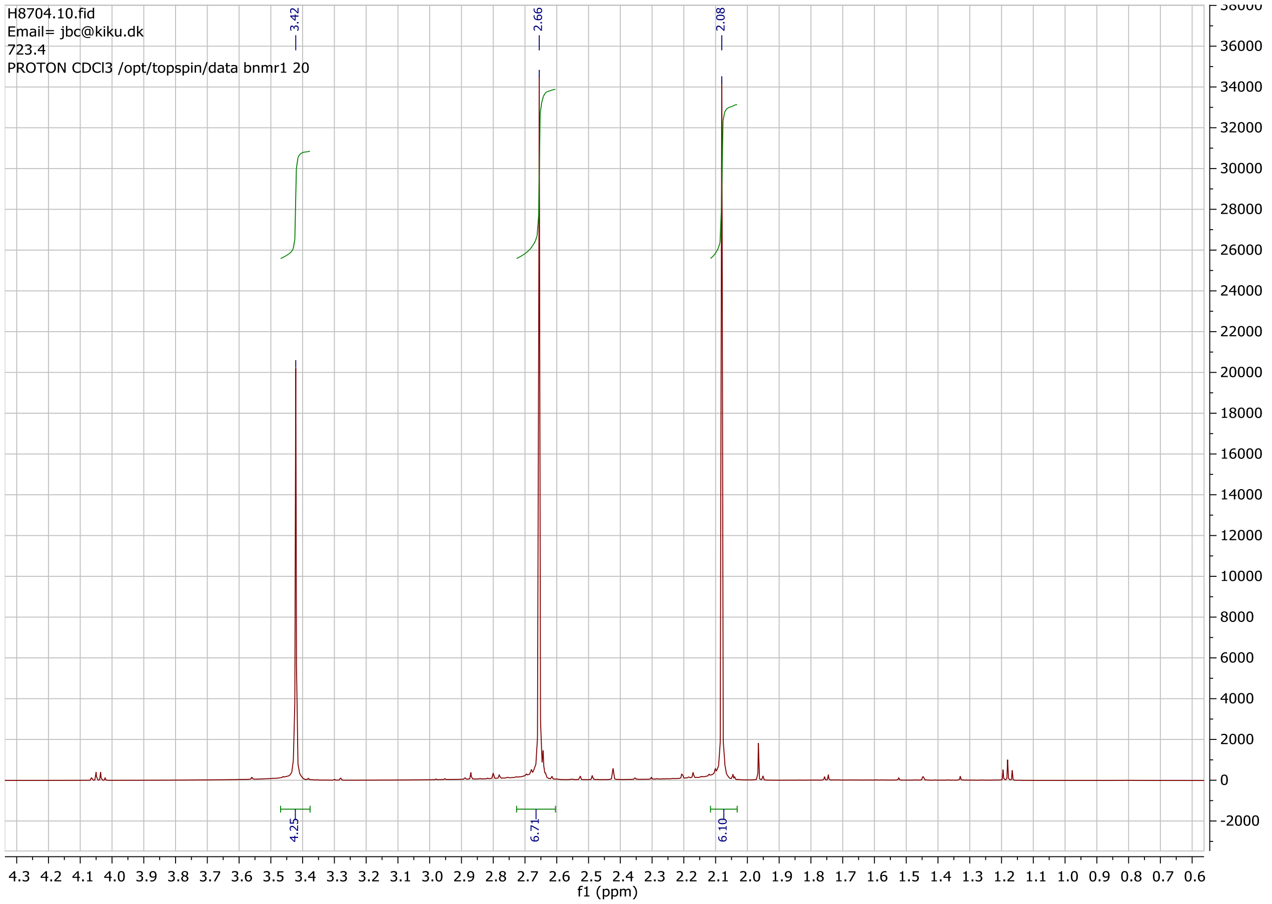
H8704.10.fid
Email= jbc@kiku.dk
723.4
PROTON CDCl3 /opt/topspin/data bnmr1 20
3.42
2.66
2.08
4.25
6.71
6.10
f1 (ppm)

COSY-NMR

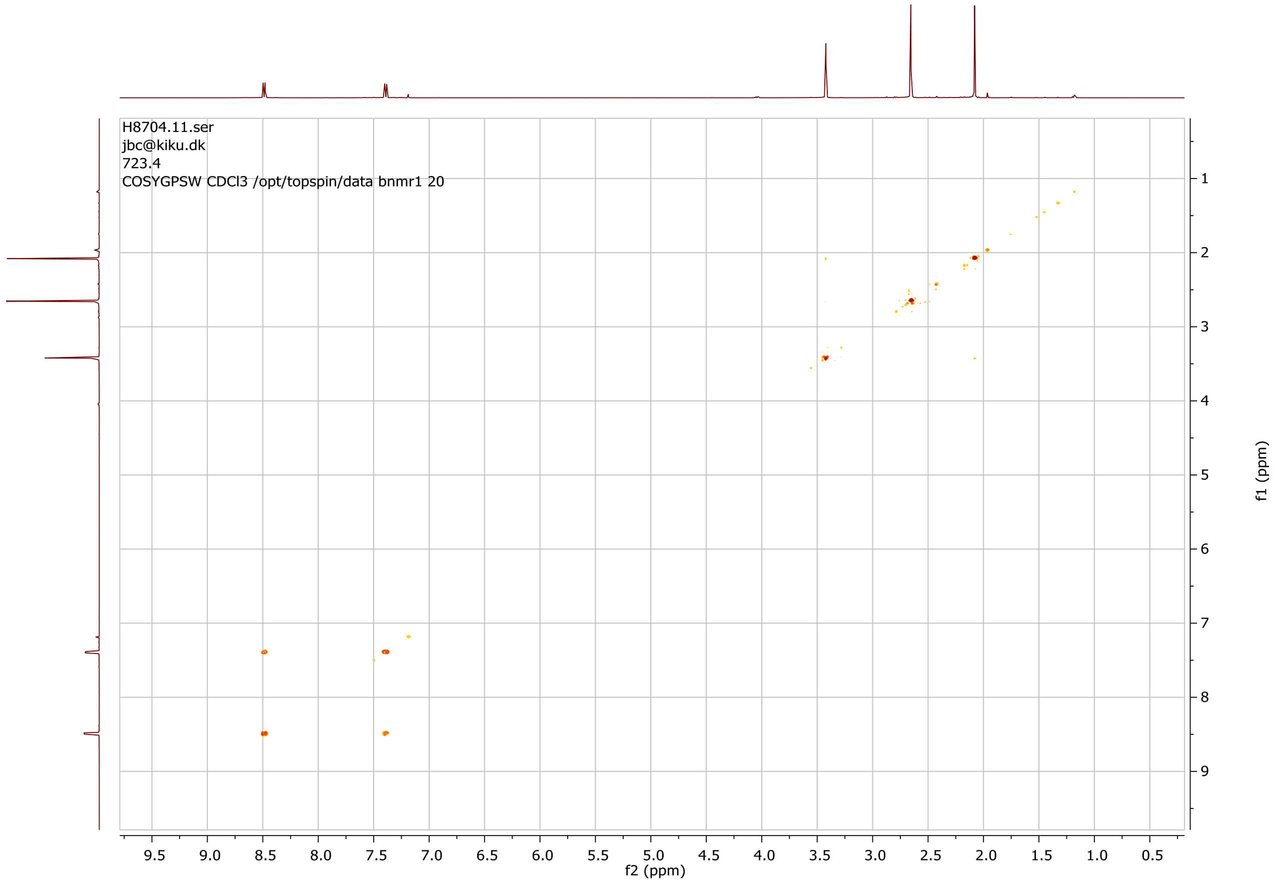
H8704.11.ser
jbc@kiku.dk
723.4
COSYGPSW CDCl3 /opt/topspin/data bnmr1 20
9.5
9.0
8.5
8.0
7.5
7.0
6.5
6.0
5.5
5.0
4.5
4.0
3.5
3.0
2.5
2.0
1.5
1.0
0.5
f2 (ppm)
1
2
3
4
5
6
7
8
9
f1 (ppm)

APT-^{13}C-NMR

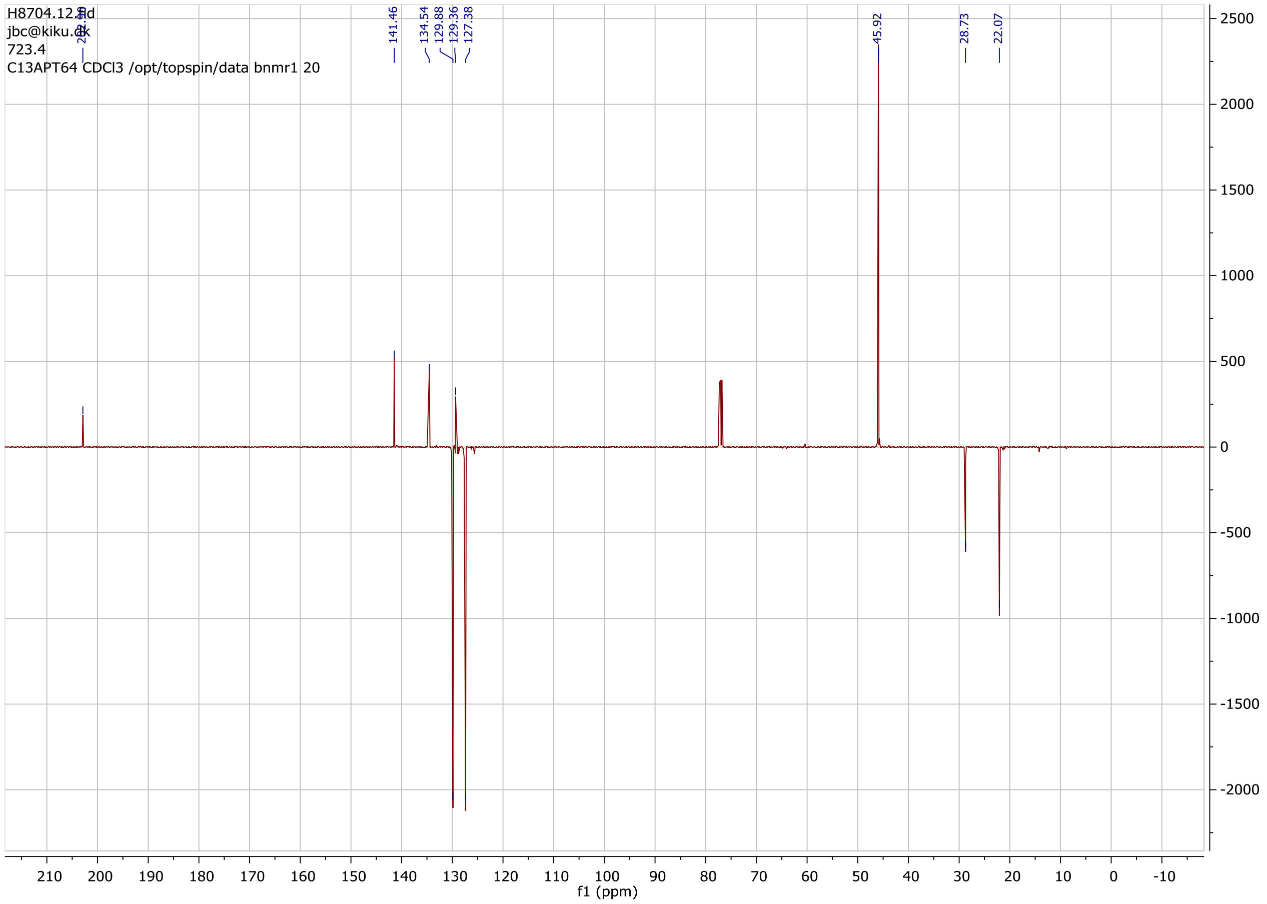
H8704.12.fid
jbc@kiku.dk
723.4
C13APT64 CDCl3 /opt/topspin/data bnmr1 20
202.90
141.46
134.54
129.88
129.36
127.38
45.92
28.73
22.07
2500
2000
1500
1000
500
0
-500
-1000
-1500
-2000
210
200
190
180
170
160
150
140
130
120
110
100
90
80
70
60
50
40
30
20
10
0
-10
f1 (ppm)

HSQC-NMR

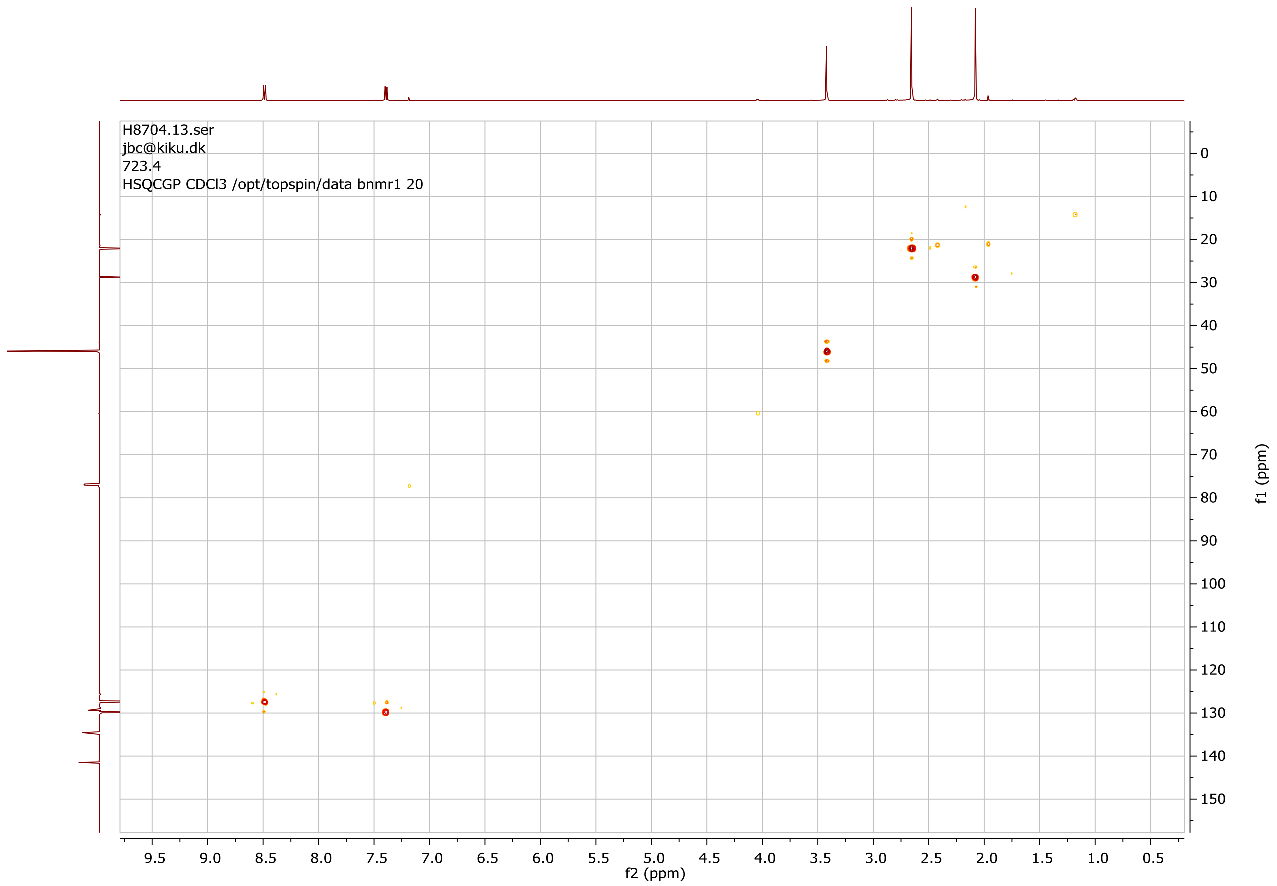
H8704.13.ser
jbc@kiku.dk
723.4
HSQCGP CDCl3 /opt/topspin/data bnmr1 20
f2 (ppm)
f1 (ppm)

NMR-data on compound **2** in CS_2 with a $CDCl_3$-lock tube

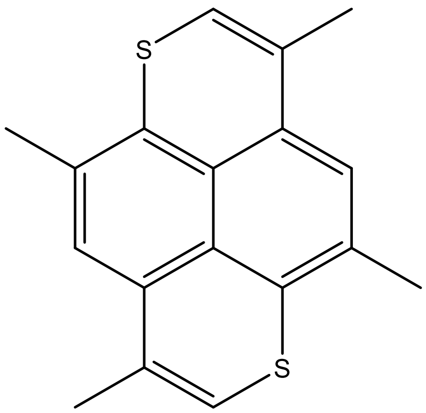

(**2**)

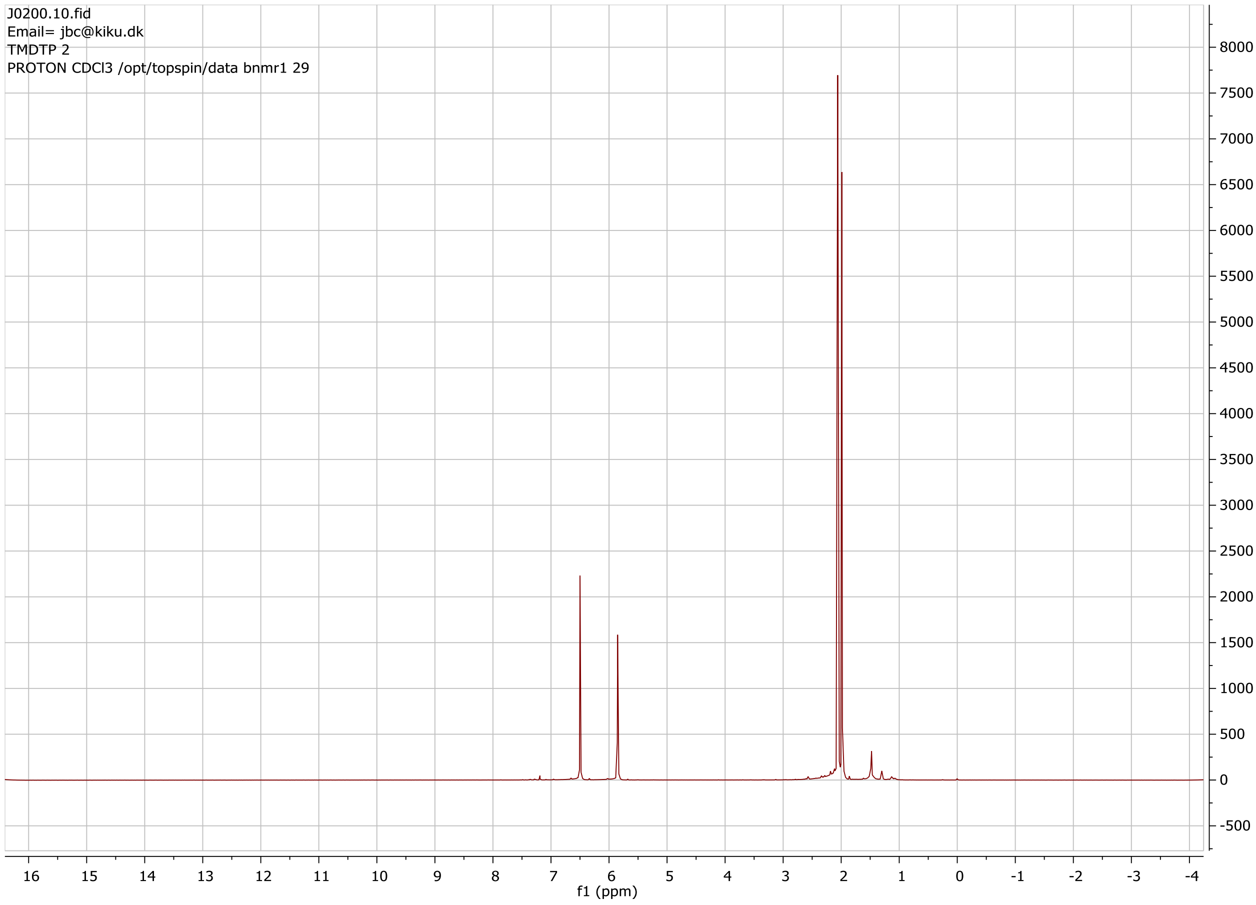

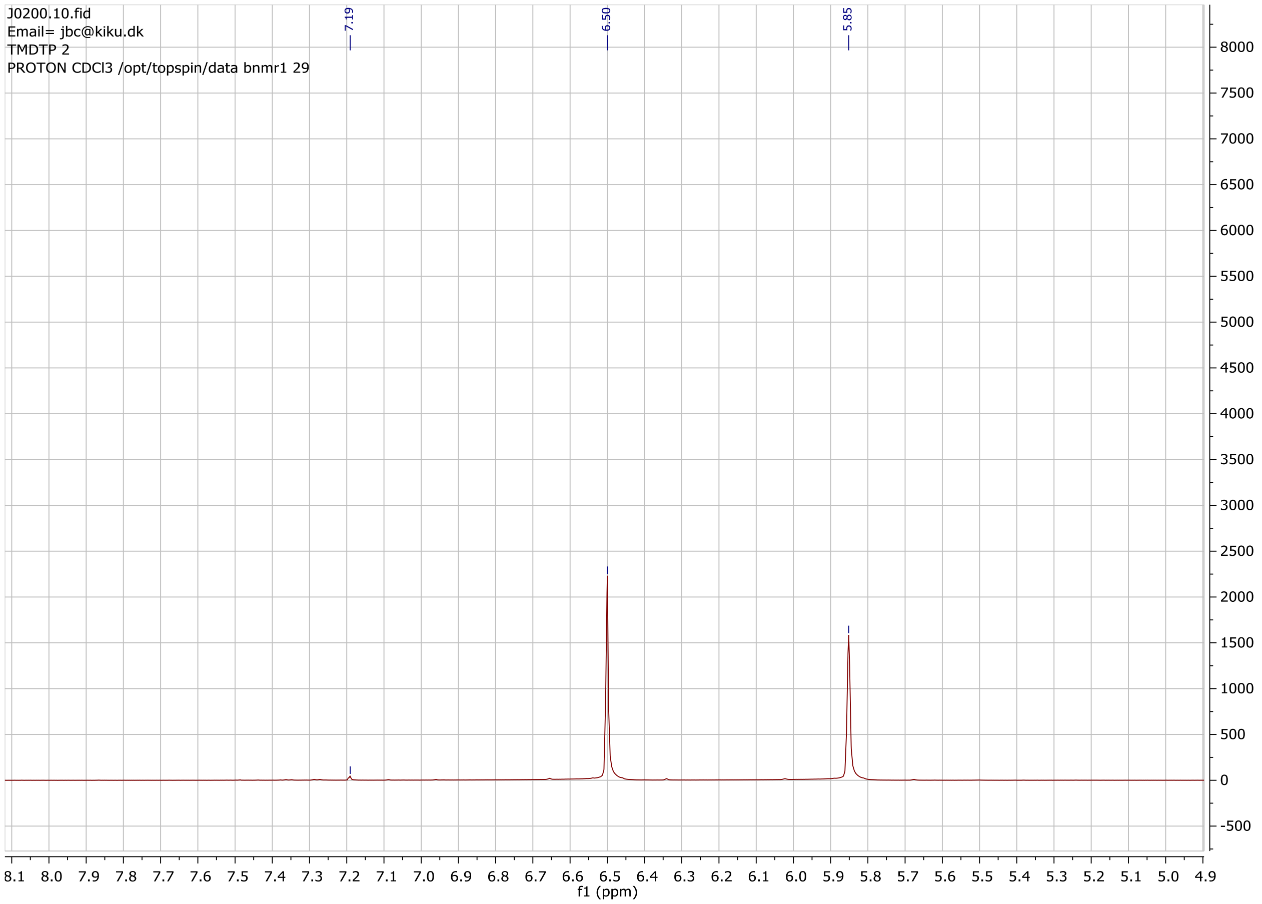
J0200.10.fid
Email= jbc@kiku.dk
TMDTP 2
PROTON CDCl3 /opt/topspin/data bnmr1 29
7.19
6.50
5.85
f1 (ppm)

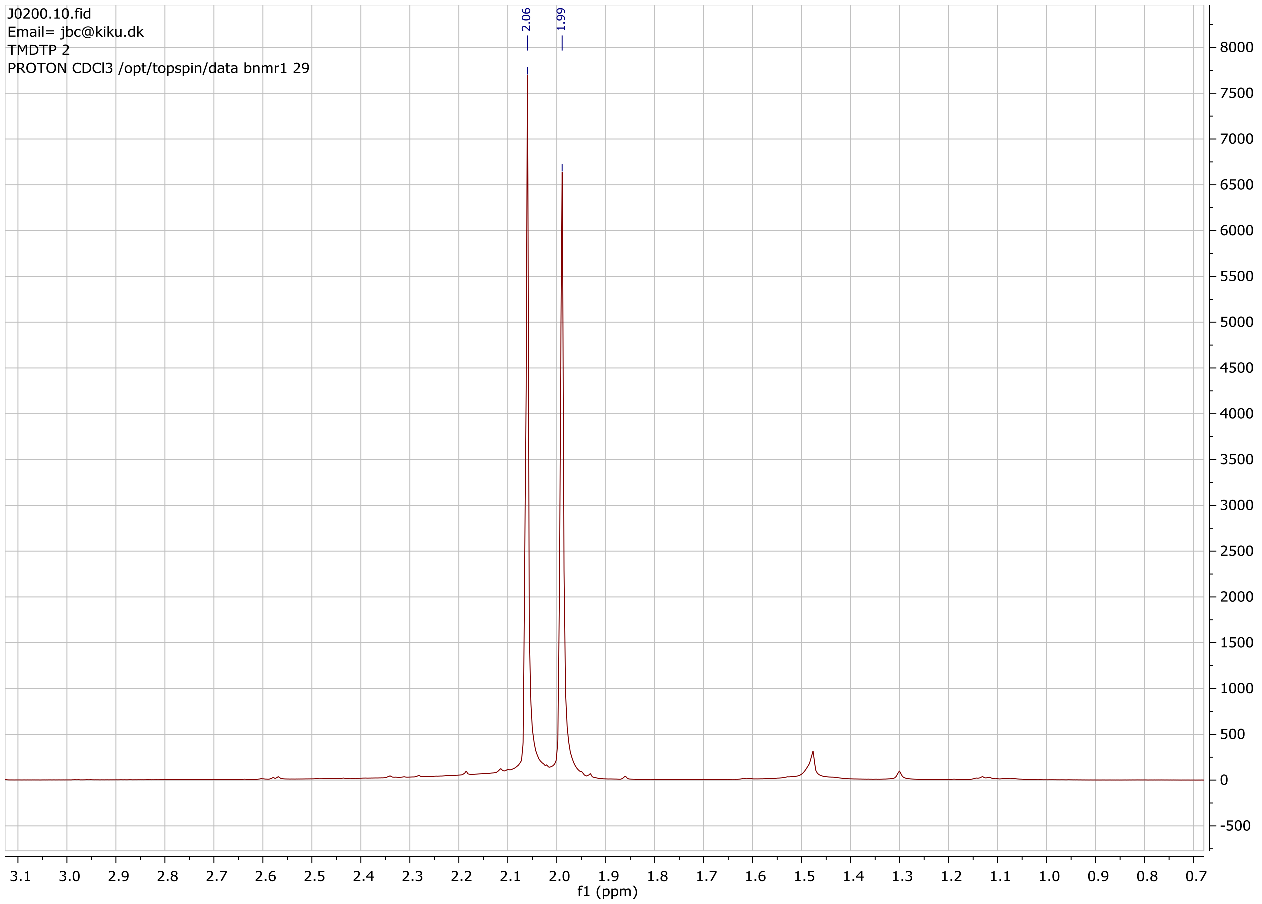
J0200.10.fid
Email= jbc@kiku.dk
TMDTP 2
PROTON CDCl3 /opt/topspin/data bnmr1 29
2.06
1.99
f1 (ppm)

COSY-NMR

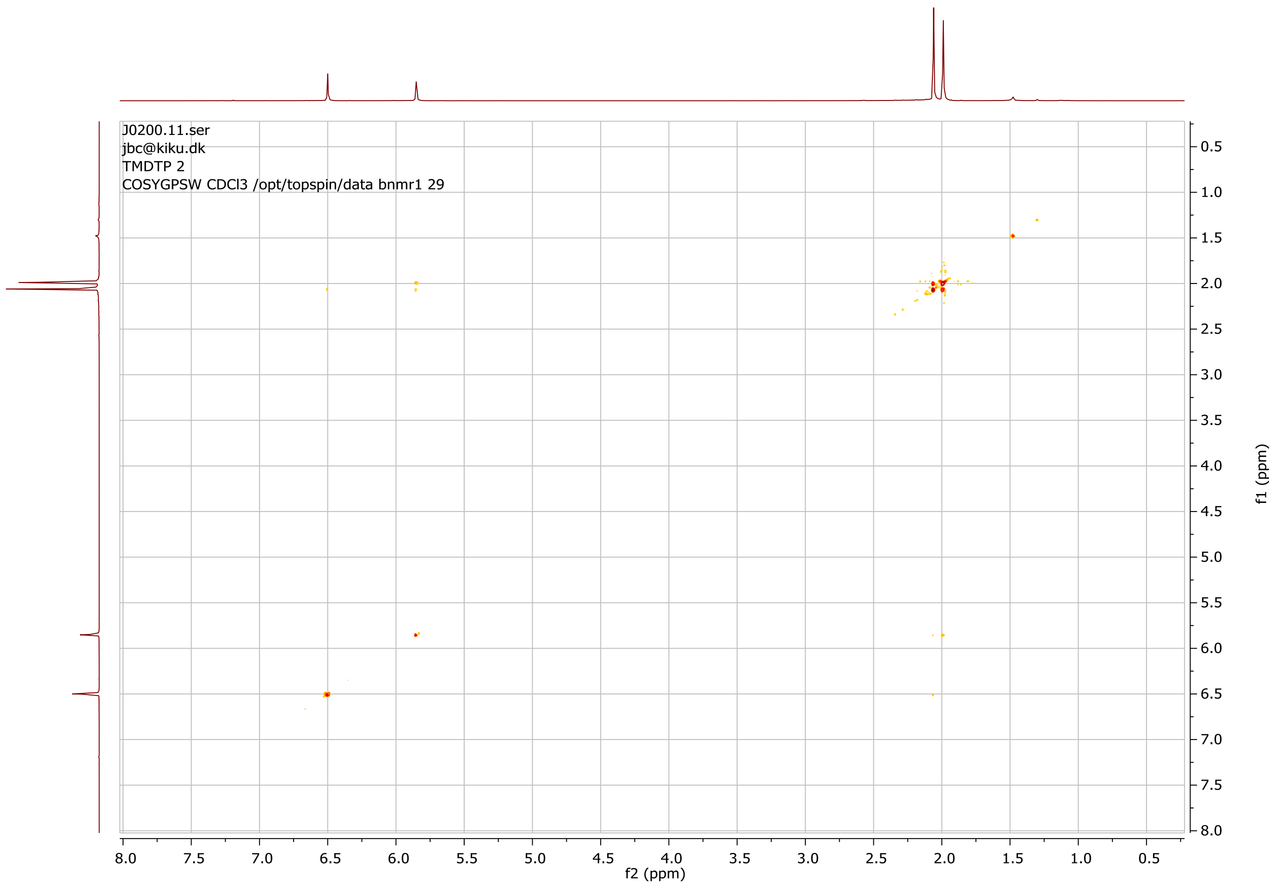

J0200.11.ser
jbc@kiku.dk
TMDTP 2
COSYGPSW CDCl3 /opt/topspin/data bnmr1 29
8.0 7.5 7.0 6.5 6.0 5.5 5.0 4.5 4.0 3.5 3.0 2.5 2.0 1.5 1.0 0.5
f2 (ppm)
0.5 1.0 1.5 2.0 2.5 3.0 3.5 4.0 4.5 5.0 5.5 6.0 6.5 7.0 7.5 8.0
f1 (ppm)

APT-^{13}C-NMR

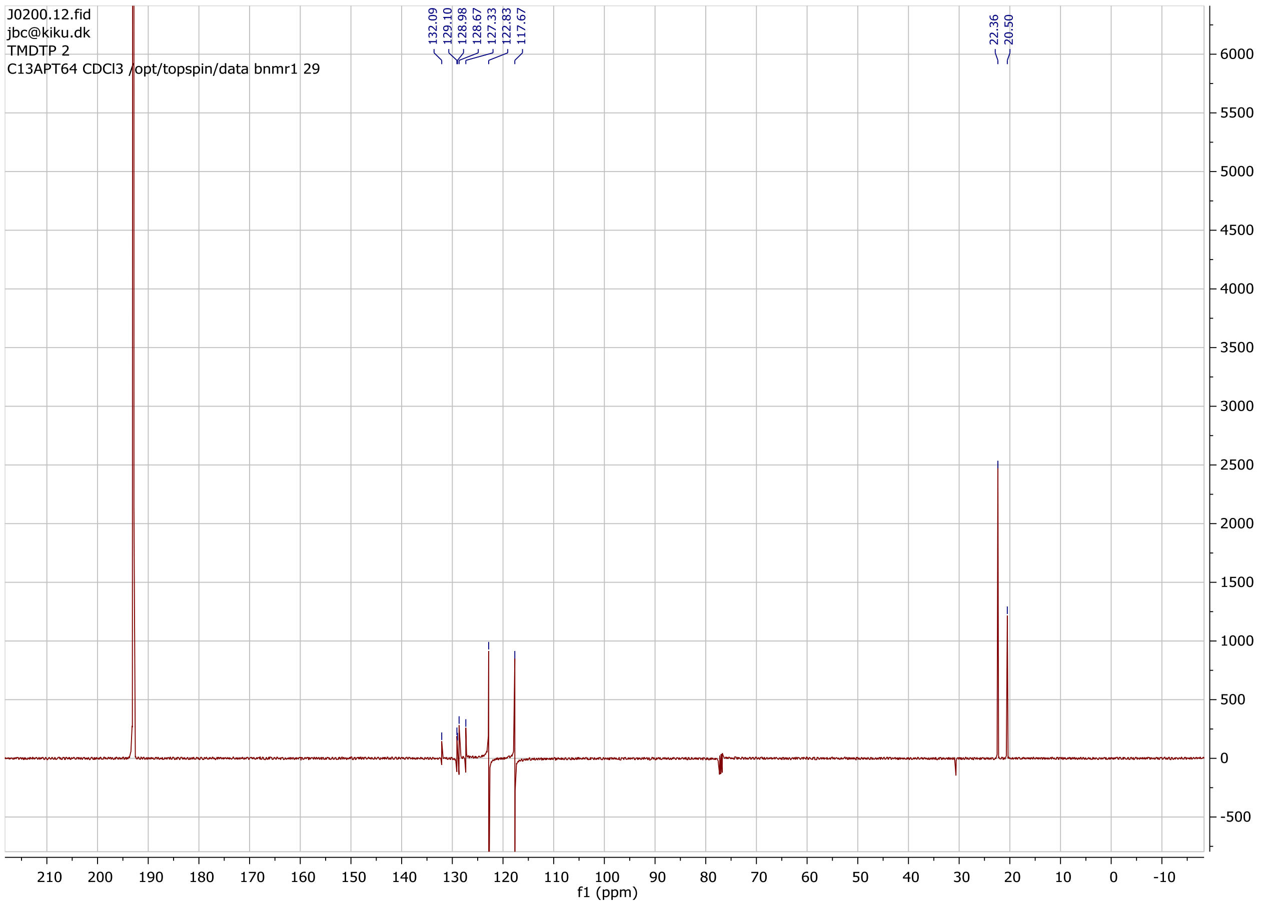

J0200.12.fid
jbc@kiku.dk
TMDTP 2
C13APT64 CDCl3 /opt/topspin/data bnmr1 29
132.09
129.10
128.98
128.67
127.33
122.83
117.67
22.36
20.50
f1 (ppm)

HSQC-NMR

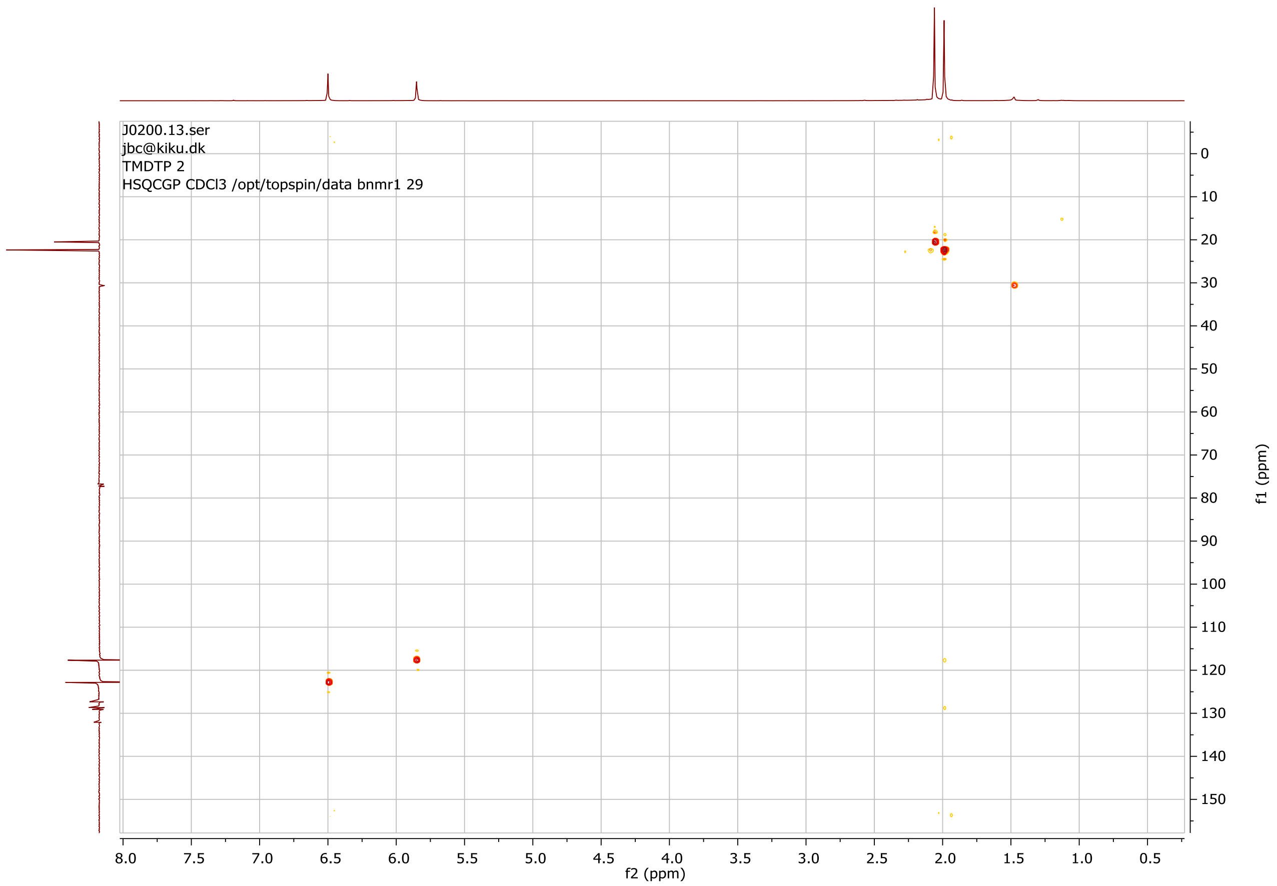
J0200.13.ser
jbc@kiku.dk
TMDTP 2
HSQCGP CDCl3 /opt/topspin/data bnmr1 29
f2 (ppm)
f1 (ppm)
